# Increasing school attendance and retention in refugee settings: Participatory development of the *SchoolLinks* intervention

**DOI:** 10.1371/journal.pone.0357215

**Published:** 2026-08-31

**Authors:** Jasmine S. Turner, Victo Kyobutungi, Hellen Ayozu, Nikhit D’Sa, Mark J. D. Jordans

**Affiliations:** 1 Department of Research & Development, War Child Alliance, Kampala, Central Region, Uganda; 2 Amsterdam Institute for Social Science Research, University of Amsterdam, Amsterdam, North Holland, the Netherlands; 3 Global Center for the Development of the Whole Child, University of Notre Dame, Notre Dame, Indiana, United States of America; 4 Department of Research & Development, War Child Alliance, Amsterdam, North Holland, the Netherlands; Washington University in St. Louis, UNITED STATES OF AMERICA

## Abstract

This study aimed to develop an intervention to improve consistent school attendance in participation with caregivers, teachers, and other stakeholders, in refugee settings in Uganda. In Phase 1, we organized four group model building workshops with caregivers and teachers in four refugee settlements to identify factors influencing attendance and caregiver engagement in education, and generate proposed actions to address them. We synthesized the outputs into a causal map and set of intervention strategies. These were reviewed through four respondent validation workshops with caregivers, teachers, school management, and representatives from refugee settlement authorities, international organisations, and local government. The resultant intervention – *SchoolLinks* – was field tested in one school with students and teachers of Primary 3 and Accelerated Education Programme 1 (government-run non-formal education supporting refugee and out-of-school children to complete primary school). Feedback was systematically collected from school staff, caregivers, children and community members to inform adaptations to improve the SchoolLinks design. Group model building revealed that attendance and retention were influenced by caregivers’ attitudes toward education and encouragement towards their children, which affected children’s motivation to attend school. Relationships between caregivers and teachers, mediated by their communication, mutual knowledge, and constrained by language barriers, influenced caregivers’ attitudes and levels of encouragement. Broader contextual factors, including poverty and lack of safety, undermined attendance and retention. SchoolLinks comprises six complementary strategies: (1) caregiver–teacher meetings, (2) meeting translation, (3) enrolment week with digital data capture, (4) attendance monitoring and incentives, (5) homework clubs, and (6) children walking in groups to school. Field testing supported the intervention’s acceptability, appropriateness, and feasibility. This multi-phase participatory approach provides insights into intervention development in refugee contexts. Future research should aim to identify the intervention’s mechanism(s) of action, analyse cost-effectiveness and cost-efficiency, engage children in further development, and assess the transferability of SchoolLinks to other settings.

## Introduction

### Education in crisis

Global education and learning are in crisis. In low- and middle-income countries (LMICs), 70% of children are experiencing ‘learning poverty’, defined as being unable to read a simple text by the age of 10 or the end of primary school [1]. Of the 234 million children and adolescents worldwide living in fragile, conflict-affected, and violent settings, only 17% are achieving minimum academic proficiency levels, more than a third (37%) are out of school completely (52% girls), and the remaining 110 million are in school but not learning sufficiently to reach minimum proficiency targets [2]. Beyond academic learning, being in school has intrinsic benefits for children’s social and emotional wellbeing, as well as access to health and other interventions utilising schools as a delivery platform – a commonality in conflict settings [3–6].

However, only 58% of children in low-income countries (LICs) complete primary school, 35% complete lower secondary, and just 22% complete upper secondary education [7]. Compounding the issue of dropout is low and irregular school attendance, which is considered an indicator or “early warning signal” of wider system instability at the student, family, school and community levels [8]. An estimated 11% of in-school adolescents globally are chronically absent [9], defined as missing 10% or more of a school year. School attendance and retention are key to both cost-efficiency and addressing learning poverty; investment in school reform and school-based interventions is only worthwhile if children are consistently in school. Meeting minimum thresholds for attendance and retention is critical to ensuring intervention quality, impact, cost-effectiveness, and scalability [10].

### Risk and protective factors for attendance and dropout in LMICs and conflict-affected settings

In LMICs and conflict-affected settings, the factors influencing school attendance and absenteeism are numerous [11]. Here we highlight the most prevalent at different levels of the socioecological system around children: the individual, the household, and the school.

At the individual level, school attendance and dropout are influenced by the child’s gender, age, disability status, mental health, and peer relationships. Boys are at higher risk of dropout in many contexts (e.g., over double the risk of girls in Pakistan, Afghanistan and Bangladesh [12]), mainly due to the desire or pressure to transfer into the workforce; however, these rates are calculated among enrolled students who are more likely to be boys to begin with [12]. Girls face numerous barriers to staying in school, including household obligations, discriminatory cultural beliefs, menstruation-related factors (e.g., insufficient hygiene materials and facilities), and early marriage and pregnancy [11,13]. Additionally, national policies in 2% of countries limit married, pregnant and parenting girls and women’s right to education, and one country (Afghanistan) bans girls’ access to secondary and higher education completely [14]. Older age and larger discrepancy between the student’s grade and age increase the risk of dropout, notably common in refugee contexts due to years out of school or delays in registration into host country’s education system [11]. Children with disabilities face infrastructural barriers and negative societal beliefs about their academic potential [11]. Experiencing stress and anxiety increases the likelihood of absenteeism by up to 60% [15], while being bullied results in 1.29 to 1.53 higher odds of being absent from school in low-middle income countries [9]. Conversely, peer support and friendships are protective against absenteeism, associated with 17% to 37% lower risk of missing school [9].

At the household level, poverty is one of the strongest drivers of school absenteeism and dropout [9,11,16]. Globally, students living in food insecure households are 43% more likely to be absent from school [17], increasing to 47% in low-middle income countries in Africa [9], and rising to 120% in Pakistan, Bangladesh, and Afghanistan, i.e., more than double the likelihood compared to children living with food security [12]. Family composition also matters; more siblings, later birth order, being an orphan or non-biological child, and living in a single parent household increase the risk of school dropout [11]. On the other hand, higher levels of parental education and employment are protective against school dropout [11]. Caregiver engagement, defined as the support of the parent or adult responsible for the child’s development towards their academic progression, is found to positively influence attendance and retention in school [18–20]. Higher caregiver support was associated with significantly lower odds of absenteeism (relative risk ratio = 0.6, p < 0.01) in Pakistan, Bangladesh and Afghanistan, and in African low-middle income countries by between 21 and 33% lower odds [9,12]. In Kenya, higher caregiver engagement was associated with student retention (r = 0.811, p < 0.01; [21]). In displacement settings, caregivers’ beliefs about the value of education drive their decision-making on their child’s schooling [19].

At the school level, physical violence on the way to or at school increases risk of absenteeism by 33–64% in African and Asian low-middle income countries [9]. Large class sizes, lacking infrastructure (e.g., desks, roofs), teacher absenteeism, and corporal punishment further undermine participation, as does sedentary learning with little interaction (e.g., OR 2.48, p < 0.001) [9,11,22]. Long distances to school risk dropout; Kremer et al. [23] found that girls’ enrolment fell by 19% for each additional mile to school, while boys’ fell by 14%. In refugee contexts, language barriers and unfamiliarity with the host country’s education system significantly deter school attendance [24–26].

Deeper understanding of the issues of chronic absenteeism and low attendance faces important limitations. First, attendance data in LMICs is sorely lacking and inconsistently measured; in an analysis of 27 national Education Management Information Systems, only eight recorded attendance [4]. Second, most literature focuses on adolescents, leaving the risk and protective factors for primary school students often assumed to be the same. Third, studies often focus on other educational outcomes, such as enrolment, grade progression, and learning, which are frequently used as proxies for attendance and retention. Fourth, there is a dearth of quantitative evidence from conflict-affected settings.

### Interventions to increase school attendance in low-resource and conflict-affected settings

In a systematic review of interventions in LMICs aiming to increase school enrolment and participation, the mean effect across intervention types on attendance was *d =* 0.15 (95% CI 0.10–0.20) and on dropout was *d* = 0.05 (95% CI 0.02–0.-09) [27]. These are considered medium and small effect sizes, respectively, within the context of education interventions [28]. Another review, exclusively of randomised controlled trials, found a median effect of 0.07 [29]. Interventions to increase school attendance and retention in LMICs and conflict-affected settings fall broadly into four categories.

First is health and hygiene programmes. In low-income settings, one review found that school feeding had a small, positive effect on attendance--a projected increase of 4–6 days per year [30]--while another found mixed evidence from studies in the Philippines, Burkina Faso, Bangladesh, Ethiopia, and Peru [31]. Emergency school feeding in refugee contexts in Lebanon resulted in up to 14% (p < 0.001) lower school dropout and 5 additional days of attendance [32], while in Mali it increased enrolment by 10% (p < 0.001) but had no effect on attendance [33]. A systematic review of menstrual hygiene interventions in LMICs found a non-significant impact on attendance [34]; in some cases they have a negative impact [31]. Qualitative evidence from displacement settings points to the benefits of Water, Sanitation, and Hygiene (WASH) interventions on educational outcomes, a conclusion supported by reviews in LMIC, though inconsistent outcome definitions prevent clear estimates of their impact [19,31,35].

Second is economic and in-kind interventions, such as school fee waivers, cash transfers or provision of school uniforms, which constitute the majority of evaluations [31]. Fee waivers alone, without accounting for other costs (e.g., books, uniforms, lost opportunity), are rarely effective [31]. A meta-analysis of conditional cash transfer programmes in LMICs demonstrates significant positive effects on school attendance (3% increase for primary and 6% for secondary) and dropout (1% lower in primary and 3% lower in secondary) [36]. Unconditional cash transfers have been shown to have no effect on school attendance in some settings (e.g., Gambia and Mexico; [37,38]), and positive but small effects in others (e.g., Syria; [39]). Though many promote the use of cash transfer programming to improve educational outcomes (e.g., [23]), the Global Education Advisory Panel [40] consider them a ‘bad buy’ in their review of cost-effective approaches to improve global learning. Disbursement of school uniforms has been shown to temporarily reduce absenteeism (e.g., in Kenya by 37%), however this effect disappeared with time [41]. In Ecuador, distribution of uniforms was found to significantly, negatively affect attendance [42].

Third are caregiver- and community- focussed interventions, such as information campaigns on the importance of education or promoting caregiver engagement. While Petrosino et al. [27] found a small, non-significant effect (d = 0.06, 95% CI −0.09–0.05) of informational campaigns, the Global Education Advisory Panel [40] classify them as a ‘great buy’, likely driven by their low cost. There is some evidence for SMS reminders as effective nudges to increase attendance; for example, SMS messages in Chile resulted in 5% more children achieving attendance requirements for grade progression [43]. Spier et al. [20] found mixed evidence in a meta-analysis of the effects of caregiver and community engagement on children’s literacy outcomes but did not examine effects on attendance and dropout. In India, a three-armed trial compared the effects of sharing information on local services with school and community members, training sessions on caregiver assessment of a child’s learning, and teacher training; absenteeism increased in all groups [44]. In Mexico, providing information to caregivers increased their engagement and improved student behaviour, and in Malawi, a family-school-community intervention improved caregiver engagement for children with disabilities, but neither study recorded effects on attendance [37,45].

Fourth is remedial and supplementary education, such as accelerated education programmes, community-based education, and interventions aimed at improving teaching quality and learning. Considering their wide use in displacement contexts, evidence for effects on attendance and dropout is sparse. In a review of what works in displacement settings, Burde et al. [19] show that only one of five remedial education programmes had a positive effect on attendance; none measured dropout. Similarly, of the 20 evaluations of socio-emotional learning, education technology, teacher training, and school management programmes, only one included attendance as an outcome (and did not show an effect) [19,46]. However, evidence from emergency settings points to positive effects of technology on attendance and retention through motivating children to stay in school (e.g., [47,48]).

We observe several limitations and gaps in interventions promoted to improve school attendance and retention in LMICs and conflict settings. First, the majority do not primarily target attendance and dropout; these are often assumed to be collateral benefits. Second, quantitative evidence for such collateral benefit is limited and inconsistent. Third, the interventions often have large administrative burden and implementation costs, e.g., school feeding and cash transfer, which likely prohibit large-scale implementation. Fourth, there is a scarcity of evidence from conflict-affected settings. These support the need for low-cost interventions developed specifically to target attendance and retention in low-resource and conflict-affected settings.

### Rationale and aim of currently study

As of July 2026, Uganda hosts over two million refugees and asylum-seekers, making it the largest refugee-hosting country in Africa [49]. Most refugees reside in 30 settlements in the North, West and Southwest of the country, bordering South Sudan, DR Congo, and Rwanda. Country-wide, 4% of children are out-of-school and the learning poverty rate is 82% [50]. Within the settlements, at least 49% of school-aged children are estimated to be out-of-school [51].

War Child (WCA) is an international non-governmental organisation which aims to improve the wellbeing and strengthen the resilience of conflict-affected children and their communities. WCA follows a systemic approach, consisting of a combination of interventions which are multisectoral (mental health and psychosocial support, protection, education), multi-level (population-wide and specialised care), and socioecological (targeting communities, schools, education staff, families, caregivers, and children, adolescents, and youth) [52]. The present study describes the collaborative design process of ‘SchoolLinks’, which is part of that systemic approach, aiming to increase students’ school attendance and retention in refugee settlements and conflict-affected communities. This research seeks to answer, “What complementary strategies, co-designed with caregivers and teachers, can positively influence children’s school attendance and retention in refugee settlements?” and “To what extent is SchoolLinks culturally and contextually acceptable, appropriate and feasible to implement?”

## Methods

Participatory action research “seeks to understand and improve the world by changing it” by engaging the very people who will enact said change, in recognition of the value of their experiential knowledge and agency in determining the appropriate actions to take [53]. It is increasingly recommended for programme development as it fosters ownership by involving participants as researchers rather than subjects [54]. In parallel, the application of systems thinking has been advocated for community-based research, intervention design, and system-strengthening efforts, particularly in low-resource and conflict-affected settings [54–57]. Under systems thinking, a system is comprised of interdependent elements that interact to form a complex whole. System dynamics is one methodology for mapping these elements and their interrelationships, drawing on feedback systems and control theory borrowed from the field of engineering [58].

Community-based system dynamics combines participatory action research with system dynamics, utilising Group Model Building (GMB) as the method to map systems [55]. GMB engages stakeholders through facilitated workshops which include 1) the use of sequential, structured activities (‘scripts’), 2) participatory dialogue to create shared understanding, 3) iterative model development, 4) model quantification, and 5) analysis for opportunities for action [59,60]. Though initially designed in high-resource settings, GMB has successfully been used in low-resource and fragile settings (e.g., Afghanistan, Pakistan, and Tanzania; [61–63].

SchoolLinks was developed in three phases: (1) GMB workshops (November 2021), (2) respondent validation and consultation (February-May 2022), and (3) field-testing (August-December 2022) in Uganda. For clarity, the design, participants, settings, procedures, and analyses for each phase are described separately in this section, and the ethical considerations, practices, and approval for all phases are described at the end.

### Phase 1: Group model building

#### Design & objectives.

We conducted four GMB workshops, with the aim of generating a comprehensive understanding of the factors affecting school attendance and retention in refugee settings in Uganda, and eliciting ideas for the SchoolLinks’ design. Each workshop was completed in one day, lasting approximately six hours.

#### Setting.

The workshops were conducted in primary schools in four refugee settlements in Uganda in November 2021. Refugees in the West Nile region (Bidibidi settlement and Rhino Camp) were predominantly South Sudanese, and the settlements in the South West region (Kyaka II and Nakivale settlements) were mainly home to refugees from DR Congo, Burundi, and Rwanda.

#### Participants & recruitment.

In each of the four schools, teachers for Primary Three (P3) and/or Accelerated Education Program 1 (AEP 1; a refugee education response to accommodate over-age learners) and 10 caregivers were invited to participate in the research (see Table 1). Fewer teachers than caregivers were selected to lower the influence of teachers on the model that was being built and increase the variation in caregiver responses. The headteacher of each school was involved in the GMB preparations but not the workshops; this was done to mitigate power imbalances.

**Table 1:** Participants in the three phases of intervention development.

	Group Model Building				Respondent validation				Field testing
Settlement	R^1^	B^2^	K^3^	N^4^	R^1^	B^2^	K^3^	N^4^	B^2^
**Total (n)** ***% female***	**14** ***71%***	**13** ***62%***	**12** ***50%***	**14** ***50%***	**24** ***45%***	**21** ***24%***	**23** ***61%***	**24** ***42%***	**320** ***61%***
Caregivers (n) *% female*	10 *80%*	9 *78%*	8 *63%*	10 *50%*	5 *80%*	5 *20%*	5 *80%*	5 *80%*	143 *78%*
Teachers (n) *% female*	4 *50%*	4 *25%*	4 *25%*	4 *50%*	4 *50%*	4 *25%*	4 *25%*	4 *50%*	3 *67%*
School management^5^ *% female*					1 *0%*	1 *100%*	1 *100%*	4 *0%*	2 *50%*
Local government & leadership *% female*					3 *33%*	4 *0%*	7 *86%*	3 *33%*	1 *0%*
Refugee management and leadership5 *% female*					5 *20%*	3 *33%*	3 *33%*	3 *33%*	1 *0%*
iNGOs^7^ *% female*					6 *50%*	4 *25%*	3 *33%*	5 *40%*	
Students *% female*									170 *48%*

Note: Rhino Camp, ^2^ Bidibidi, ^3^ Kyaka II, ^4^ Nakivale, ^5^ Including headteachers (mainstream and AEP) school management committee and parent-teacher association members. ^6^ Including UNHCR, Office of the Prime Minister, Refugee Welfare Counsellor. ^7^ international Non-Governmental Organisations

Recruitment started on 1^st^ November 2021 and completed on 18^th^ November 2021. Caregiver selection was based on previously collected data of 50 randomly selected students from P1 to P7 and AEP 1 on children’s home learning environment, which measured caregiver and community support towards children’s reading and numeracy skills inside and outside the home. Students were ranked from the lowest to highest home learning environment score, and the lowest, the highest, and eight interval positions in between, were selected to achieve a sample representing a spectrum of home learning environments. The caregivers of these children were invited to participate in the GMB workshops, totalling 10 per workshop. Their characteristics were checked against those of the rest of the P3 class to ensure approximate representativeness by gender, age, nationality, and relationship to child. At the start of each workshop, the research and the details of the consent form were presented and any questions answered, after which written consent was obtained from participants. In cases where a participant was illiterate, verbal consent was obtained in the presence of a witness known to the participant.

#### Procedure: Structured activities and data collection.

*Workshop facilitation roles:* WCA engaged Dynamundo, a consultancy organisation specialising in GMB and community-based system dynamics, to co-facilitate the workshops with WCA researchers. Two teams (one for West Nile, one for Southwest) of five research assistants, recruited from the refugee and nearby host communities, were given a one-day induction into GMB. In the workshops, participants were grouped according to language preference and supported by the research assistant fluent in that language. The school headteacher opened the workshop in each setting, encouraged participation and openness, and then left.

The workshops consisted of four structured activities: 1) variable elicitation, 2) categorisation, 3) connection circles, and 4) action ideas.

*Activity 1: Variable elicitation:* Variable elicitation aimed to generate a list of the factors affecting school attendance, retention and caregiver engagement from participants, guided by the questions: “What affects how much a caregiver/parent can be involved in their child’s education? What (else) affects children’s school attendance and retention?”

*Activity 2: Categorisation:* The second activity aimed to categorise the variables into themes. In the first workshop, this was attempted through group discussion but consecutive translation into multiple languages made this slow and wearisome. In subsequent workshops, the facilitators opted instead to propose categories based on those of the first workshop: 1) knowledge and attitudes, 2) communication, 3) caregiver and household context, 4) the school context, and 5) the child’s context – which participants then assigned variables to during their discussion.

*Activity 3: Connection circles*: Connection circles aim to depict the direction (causal relationship) and type (or polarity) of relationships between elicited variables. Positive polarity is when a change in one variable causes the same directional change in another (e.g., higher school meeting attendance leads to more teacher feedback to caregivers; lower attendance leads to less feedback). Negative polarity means a change in one variable causes an opposite change in another (e.g., increased language barrier leads to lower communication between caregivers and teachers). Participants were divided into subgroups, and each subgroup assigned a category. The categories’ variables were written around the edge of a circle. Participants identified the direction of relationships and polarity between variables through discussions facilitated by the research assistants. These relationships were depicted through arrows and + or – symbols on the connection circles.

*Activity 4: Action ideas:* The last activity aimed to generate actions that could positively influence the system. Participants used the connection circles to identify leverage points for intervention, such as variables having negative, systemic impacts (e.g., language barrier) or positive feedback loops (e.g., caregivers’ meeting attendance increases home-school communication, which positively influences caregiver-teacher relationship, which motivates caregiver meeting attendance). Participants volunteered ideas for intervention, and the intention was to classify these on a grid showing perceived impact (high vs. low) and implementation feasibility (easy vs. difficult), however there was not always time for this final step.

#### Data and analysis.

We documented the participants’ stories through field notes and the connection circles through photographs, and extracted the variables and causal links (see S1 Table for all variables and S2 Table for the causal links). We produced causal loop diagrams (n = 20) – visual representations of the connection circles – for the five categories of each workshop (see S1 Fig for the causal loop diagrams). Causal loop diagrams include reinforcing and balancing feedback loops. For example, increased communication between caregivers and teachers leading to more positive relationships between them, which in turn leads to increased communication, is an example of a reinforcing loop. Increased household wealth leading to increased payment of school fees, which leads to reduced household wealth, is an example of a balancing loop. We compared the causal loop diagrams across the four settings and synthesized common or salient factors into an overall ‘causal map’. Next, we collated action ideas (see S1 Text for the full list); ideas that were outside of WCA’s remit, e.g., construction of schools or teacher training, were excluded, as were those that would require high, sustained financial input, such as provision of school fees and uniforms. Ideas which targeted variables that had negative influence on the system or those linked to many other variables (and therefore could generate ripple effects) were prioritised or combined to form strategies, the basis of the intervention design.

### Phase 2: respondent validation & consultation

#### Design & objectives.

Respondent validation, also known as member checking, is a process where researchers share findings or interpretations with participants to confirm their accuracy and enhance the credibility of qualitative research [64]. This, along with additional stakeholder consultation, was done through workshops (n = 4) in Uganda, which aimed to: (1) validate the causal map, and (2) gather feedback on strategies. A fifth workshop with WCA staff aimed to generate agreement on the strategies that would be field tested, within the remit of WCA’s expertise and mandate.

#### Setting & participants.

Four respondent validation workshops were held in February 2022 in the same refugee settlements as the previous phase. In each workshop, participants included the same four teachers and a selection of five caregivers who had participated in the GMB workshops, and the head or deputy headteacher. Additional representatives joined from UNHCR, the Office of the Prime Minister (the government agency for refugee management, disaster response, and policy implementation), non-governmental organisations, and the District Local Government (see Table 1). Recruitment started on 9^th^ February 2022 and concluded on 5^th^ March 2022. Following a presentation of the research and the details of the consent form, written consent was obtained from all participants in writing. In cases where a participant was illiterate, verbal consent was obtained in the presence of a witness known to the participant. A fifth workshop was held online in May 2022 with eight participants including WCA education advisors, programme managers, and researchers; as this was considered work as usual within the process of intervention development, consent was not obtained but participants were informed how their collective input would be used.

#### Procedure & analysis.

In the workshops in Uganda, the causal map was reviewed with participants, who were then divided into groups (caregivers & community leaders, school staff, and partner representatives) to discuss and rate the strategies resulting from the GMBs in relation to their potential feasibility, perceived impact, sustainability, and overall design. Notes and debriefs after each workshop compiled participants’ input and were used to articulate and refine the design of the strategies.

The online consultation with WCA staff lasted approximately one hour, and included a presentation of the research to date, the causal map and the strategies, and feedback from the respondent validation workshops. Decisions were taken on which of the eight strategies to field test, and their implementation protocols were articulated.

### Phase 3: field-testing

#### Design & objectives.

The selected six strategies, now collectively known as ‘SchoolLinks’, were field tested between August and December 2022 using a prototyping approach: iterative cycles of implementation, feedback and adaptation. This aimed to (1) assess the acceptability, appropriateness, and feasibility of implementation, and (2) to document the intervention protocol.

#### Setting & participants.

The field-test was conducted in Bidibidi refugee settlement, West Nile, over one school term in the same school which had participated in Phases 1 & 2. This was to maintain continuity with participants and for convenience, as other education research was ongoing in schools in South West which could have confounded the results of the field test, and Rhino Camp was too far from the WCA office for the research team to be able to reach with the frequency needed for the field testing. SchoolLinks was implemented with teachers, caregivers, and students enrolled in P3 and AEP 1. The headteacher, Parent-Teacher Association and School Management Committee, and community leaders were also involved. The headteacher and majority of school staff were Ugandan nationals, while most of the caregivers, students, and community members were South Sudanese.

Following the consecutive implementation of each strategy, caregivers, students, and teachers were asked for feedback and suggestions for improvement. Participant selection was done pragmatically, meaning that there were no specific criteria but varied perspectives were sought. For example, we sought out participants who expressed concerns, dissatisfaction or challenges, as well as those who had the opposite experience. Additionally, many participants actively sought out the research team to give their feedback, likely as a result of explaining the research during the first meeting with caregivers and teachers. Two community leaders – one from the refugee settlement and one from the host community – frequently sought out research team members to discuss progress and give feedback. Consent was not obtained from participants in the field-test as it was part of routine monitoring of WCA’s programming, and because individual’s opinions and feedback were collated and collectively reflected in field notes and names were never recorded. Participants were consistently made aware of their right to not partake in the intervention and requests for feedback.

#### Procedure, data, and analysis.

Meticulous field notes recorded the implementation protocol of each strategy, success and challenges, and participants’ feedback. These were used to inform strategy adaptations, based on discussion and consensus amongst the research team. The adapted strategies were then implemented and the cycle repeated, except the Enrolment strategy as enrolment only occurs once a term.

### Ethics

Ethical review and approval for Phases 1 and 2 was received from Makerere University’s School of Social Sciences’ Research Ethics Committee (03.20.394) and Uganda’s National Council of Science and Technology (SS451ES). Prior to each activity, information sessions were held in different languages to explain the aims, methods, risks, and requirements of the research, data management and confidentiality measures, and gather written or witnessed consent (the latter for non-literate participants only). Contact details of WCA staff and representatives of the ethical review boards were shared so that participants could directly communicate concerns and questions.

Ethical review and approval for Phase 3 was conducted by The AIDS Support Organisation (TASO-2022–136) and Uganda’s National Council of Science and Technology (SS1378S). Additionally, the research and SchoolLinks were presented to the refugee settlement and school management bodies to gather their approval before commencing the field test, including Office of the Prime Minister, community leaders, the School Management Committee, and Parent-Teacher Association. In the first meetings with school staff and caregivers, attendees were given the option to opt their children (and themselves) out of the intervention and the research. Contact details of WCA staff, field office, and representatives of the ethical review boards were also shared. Additional information regarding the ethical, cultural, and scientific considerations specific to inclusivity in global research is included in the Supporting Information (S1 File).

## Results

### GMBs: Casual map and action ideas

**Fig 1:**
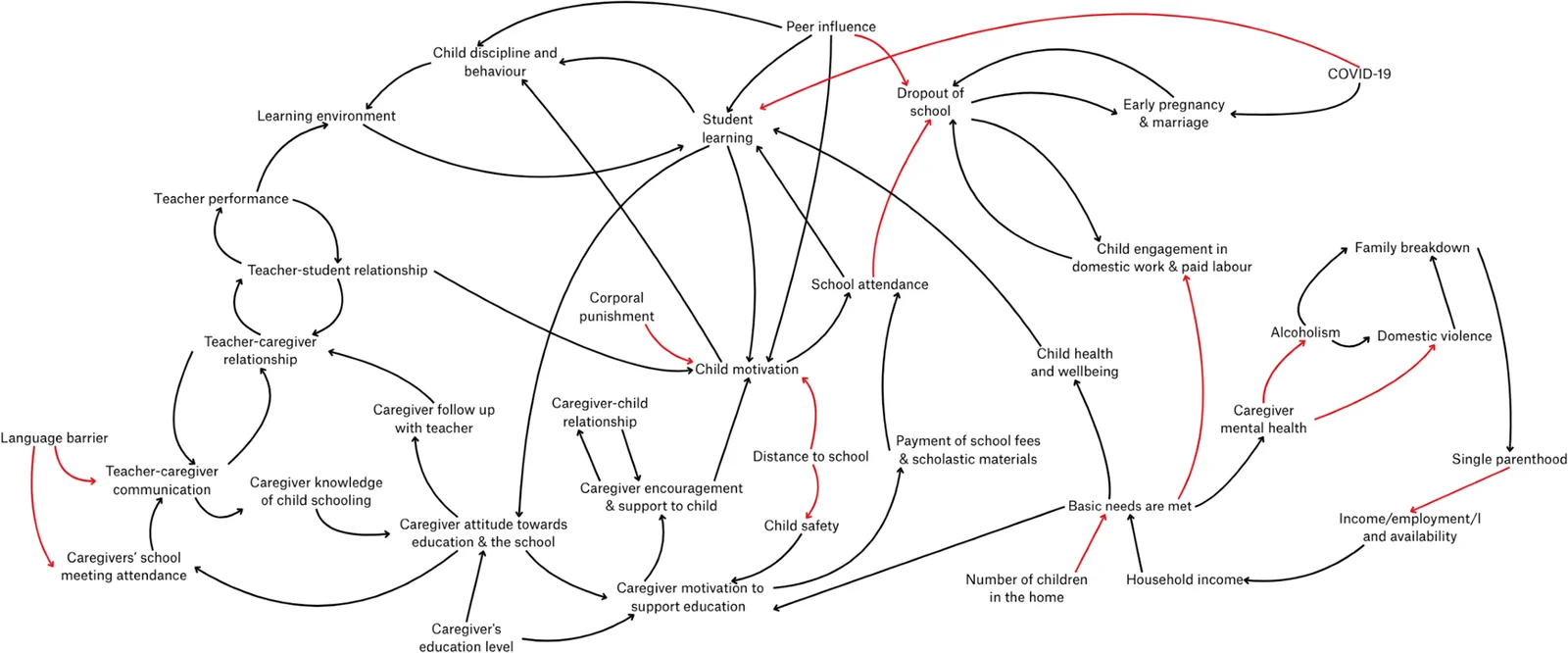
Causal map. Caregivers’ and teachers’ depiction of factors influencing school attendance, dropout, and caregiver engagement. Black arrows depict positive polarity, red arrows depict negative polarity.

Participants were highly engaged in the GMB workshops and the rich discussions contributed to detailed causal map for school attendance, retention, and caregiver engagement in refugee settlements in Uganda (see Fig 1). Time constraints meant that not all activities were systematically completed for each workshop; we prioritised gathering variables, developing the connection circles, and compiling action ideas over participant-led categorisation of variables and rating the actions ideas, which we did in the respondent validation workshops instead. Many examples and stories were shared (see Table 2 for example stories and the extracted variables, S1 Table for all variables generated, and S2 Table for the causal links) to provide nuance to the descriptions that participants provided. On average, each workshop produced 70 variables with 142 causal links. Where appropriate, variables were recoded; for example, the availability of food, soap, kerosene, and menstrual hygiene materials were recoded as ‘basic needs are met’, leaving 78 unique variables. Of these, only 13 were mentioned in a single location. To determine the variables included in the map, we identified those that were mentioned in three or more locations, resulting in a causal map consisting of 38 variables (see Fig 1).

**Table 2:** Example stories and variables from GMB workshops.

Story	Variables
*“Good performance leads to motivation of the parent because they know that their child is interested in education.”*	student learning; caregiver motivation to support education; child motivation.
*“Lack of support from the husband towards education, like funds for uniform or shoes, makes it difficult for her to support her children at school. Even moral support – many women are here at the settlement alone, husbands are in South Sudan, and do not even ask their wives how the children are doing, at school. Raising this issue opens a can of worms, especially when he is drunk. When the husband cannot support, he quarrels in order to shut down the wife.”*	basic needs & scholastic materials; caregiver encouragement and support to child; single parenthood; caregiver knowledge of schooling; over-drinking/alcoholism; family breakdown.
*“When the distance is far to school, through bushes, there have been cases of rape and kidnap– parents fear to send the child to school and fear to escort the child to school, and so tell the child not to go to school”*	distance to school; child safety; caregiver motivation to support education; caregiver encouragement to child.
*“Without soap at home, we cannot clean uniforms which get dirty on the walk to and from school. If children go with dirty clothes, they will be bullied at school and family will be embarrassed.”*	availability of basic necessities; availability of scholastic necessities; bullying; family reputation.

There were differences between the four locations’ categorisation of variables and causal loop diagrams (see S1 Fig for all causal loop diagrams), but several common themes emerged. First, the household financial status was a key determinant of caregivers’ support to their children’s education. Caregivers are constantly faced with decisions on how to allocate limited financial resources, balancing between school-related expenses (e.g., school fees, scholastic materials) and other basic needs. This strain is exacerbated in single-headed households, and linked to drug and alcohol usage, domestic violence, child labour and school dropout.

Second, caregivers’ attitudes and knowledge of their children’s education directly influence their prioritisation of spending money on school fees. Positive attitudes and increased engagement can lead to higher child motivation and attendance, whereas negative attitudes risk the child being kept at home to support chores or not encouraged to attend school.

Third, caregivers’ attitudes and knowledge are influenced by their communication and relationships with teachers, which in turn affect the teacher’s relationship with the student. However, language barriers between school staff and refugees (and between refugees) significantly challenge this communication. Teachers face this challenge in the classroom with their students, as well as large class sizes and limited resources (e.g., desks, teaching materials). The relationship between teachers and students affects children’s attendance and behaviour in class, influenced by teachers’ use of corporal punishment.

Fourth, low school attendance is a predictor of dropout, which in turn increases risk of early pregnancy, child labour, and future unemployment. Children’s attendance, motivation and academic progression have a cyclical reinforcing relationship, where the causal relationship between motivation and achievement is mediated by attendance. Children’s motivation and academic achievement influence caregivers’ attitudes and behaviour.

Many action ideas were shared (see S1 Text for full list). Those that aimed to increase communication between teachers and caregivers, teachers’ knowledge of children’s home context and individual needs, and caregivers’ knowledge of their child’s education were prioritised, as were those that addressed unsafety on the way to school. Some action ideas were combined to form strategies, for instance regular attendance monitoring by the teacher and gifting children with basic or scholastic necessities were combined into a conditional reward strategy. Eight strategies were identified for the next phase of development, including: 1) organising meetings between school staff and caregivers, 2) translation at these meetings, 3) digitised enrolment, attended by caregivers, 4) attendance monitoring and conditional rewards, 5) homework groups supervised by caregivers, 6) caregivers accompanying children to school, 7) caregiver volunteers in the classroom, and 8) support for a community income-generating activity. Other suggested action ideas, such as parental education on discussing sexual and reproductive health, formed a suggested agenda of topics for discussion in the meetings between caregivers and school staff.

### Respondent validation & consultation

In the validation workshops, participants confirmed the validity of the variables included in the causal map as those affecting school attendance, as well as the relationships depicted between the variables (see S2 Text for the compiled workshop notes). Some suggested additional variables for inclusion, such as distance to school, cultural beliefs related to underage marriage, and disability. Where these were repeated across workshops, or reflected input from the GMB workshops, the additional variables were incorporated into the causal map.

The meetings strategy was considered the most feasible and impactful, rated ‘high’ on both criteria across participant groups (caregiver, teachers, partners) and locations. Translation was rated high impact by all, but some respondents questioned the feasibility of relying on volunteers. The strategy of caregivers enrolling their children was considered high impact by all except one group in one location, who believed it would have low impact on attendance. Most respondents rated it highly feasible, however teachers flagged that it would not be possible to do before the start of term, as teachers return home during school holidays. Attendance monitoring and rewards were universally rated as high impact and participants suggested soap, exercise books and pens, and menstrual hygiene products as rewards that would motivate attendance and have broader benefits. However, there was mixed feedback on its feasibility; many participants questioned how rewards would be decided, cautioned against the sustainability of the strategy, and proposed alternative systems for awarding rewards (e.g., needs-based or achievement-based).

Homework groups supervised by caregivers were rated by most participants as high impact, believing they would enable peer-to-peer learnings and foster friendships between children, but moderate or low feasibility due to reliance on volunteers, differing school policies on homework, and questions about appropriate locations. Similarly, accompanying children to school was rated as high impact but low feasibility by most participants, attributed to timing issues as many caregivers tend to their vegetable gardens in the early morning. Children walking to school together in groups or accompanied by older siblings was proposed as a more viable alternative. Volunteer support in classrooms by caregivers was unanimously voted high impact in the belief that it would support teachers’ workload and positively influence children’s behaviour and was rated feasible by teachers but unfeasible by most partners representatives and caregivers. Feedback stressed that caregivers would need to be paid and that they would face language barriers with students. The final strategy – an income-generating activity – was rated high impact but low feasibility by most participants. Concerns were raised about its financial sustainability, how many caregivers would be able to benefit through it, and the training it would require.

**Fig 2:**
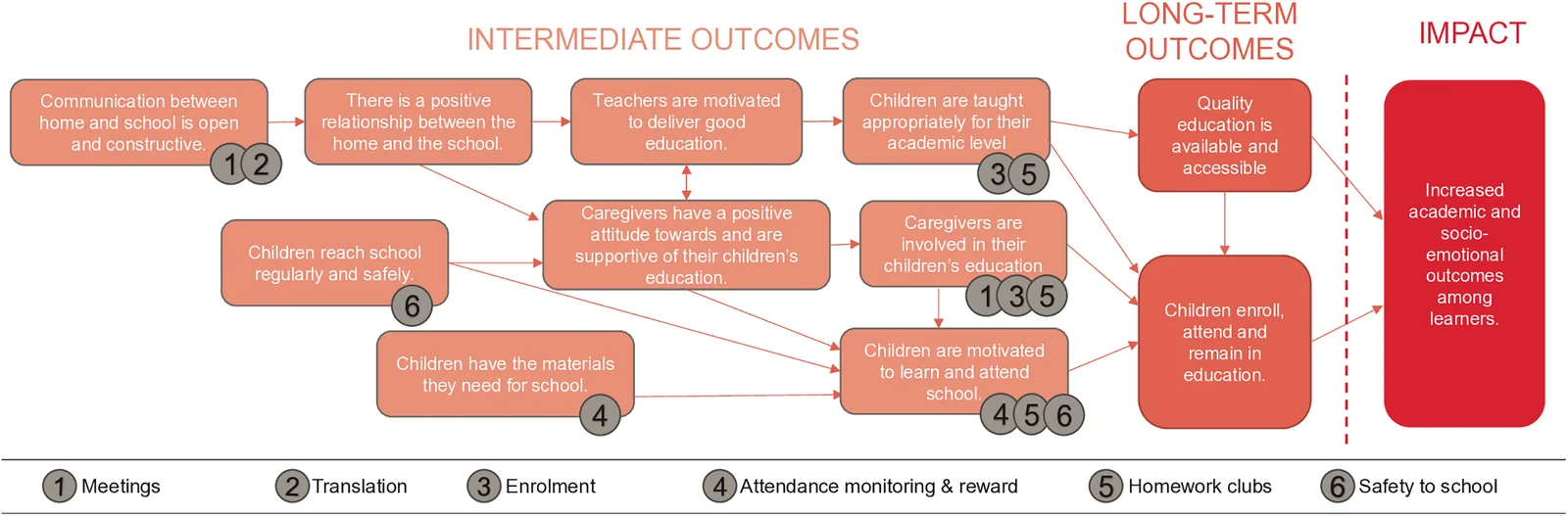
Theory of change of schoollinks.

In the WCA workshop, two strategies were considered unfeasible from an organisational perspective: volunteer classroom assistants would need to undergo a recruitment process to ensure adherence to child safeguarding principles, which would substantially increase the intensity of the strategy. Similarly, support for an income-generating activity was considered high intensity and outside of WCA’s technical expertise. Concerning rewards for attendance, the group cautioned that providing menstrual hygiene products without accompanying education, community sensitisation, and facilities, created a risk of harm. The remaining six strategies were implemented for field-testing and reflected in a Theory of Change which was developed and refined over the course of the GMBs and respondent validation process (see Fig 2).

### Field-test

#### Intervention.

**Fig 3:**
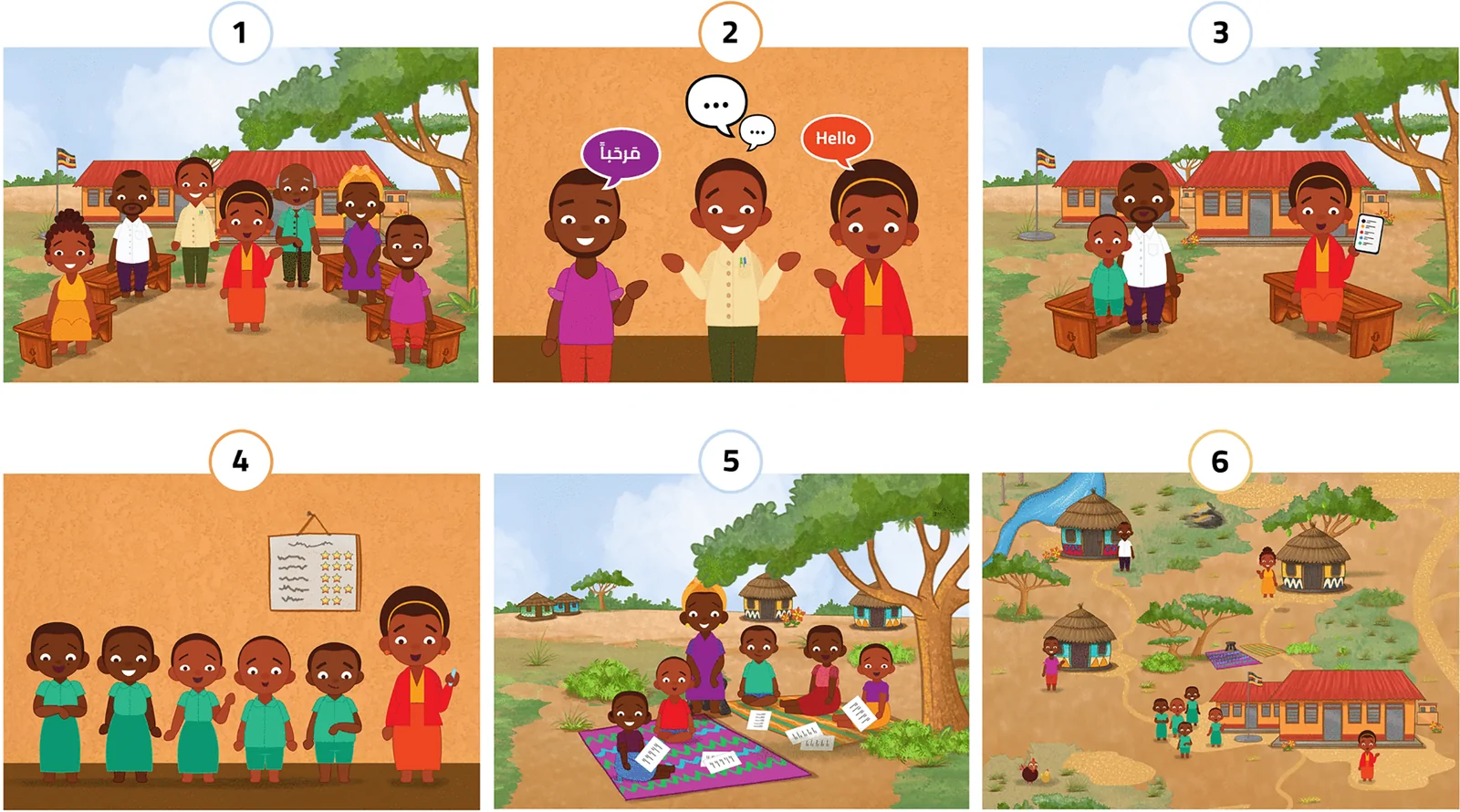
Visual of the SchoolLinks strategies. (1) Meetings, (2) Translation, (3) Enrolment week, (4) Star charts and rewards, (5) Homework Clubs, (6) Safety to School.

Following a series of preparatory meetings with the school headteacher, SMC, PTA, community leadership, and translators, the first Meeting (Picture 1 in Fig 3) was held at the end of Term 2 to introduce SchoolLinks and discuss the importance of education and caregivers’ engagement. Translation (Picture 2 in Fig 3) was done by two volunteers in Juba Arabic and Bari, though additional translation needs were checked at the start of each meeting. Posters of the six strategies supported the discussion (see Fig 3).

Six teachers were given a half-day training on using a tablet for enrolment data collection a week before Enrolment week (Picture 3 in Fig 3), which was held in the first week of the school term, during which enrolment is normally done. Caregivers who arrived alone or had asked neighbours/friends to register their child were asked to return with their child. Teachers entered children’s registration data on tablets, including their name, date of birth, home address, caregivers’ contact details, the Washington Group Short Set on Functioning [65], and two questions on the home context and specific needs of the child. Most students (61%) were enrolled in the first 3 days, and then a tablet was left at the school for additional enrolments.

Students’ names were extracted from the enrolment data to place on the charts for monitoring attendance – called ‘star charts’ – which were made from Velcro and laminated sheets so that they were reusable. Teachers collected attendance every morning and afternoon, and on Fridays monitored weekly attendance; students who attended four days in a week were given one star to place by their name on the star chart, those who attended five days were given two stars (Picture 4 in Fig 3). After 4 weeks, students with seven or eight stars – equivalent to 95–100% attendance – were given a bar of soap. Two cycles of attendance monitoring were completed per term, in the second cycle children were rewarded with a pen and exercise books.

A second Meeting was held near the start of the third term to remind caregivers about attendance and rewards, homework clubs, and safety to school. Teachers used the enrolment data and their knowledge of their students to group children into Homework Clubs (Picture 5 in Fig 3), which met during the weekends and were supervised by volunteer caregivers (who volunteered themselves in the second Meeting). These same groups of children agreed on a time and place to meet each day and walk to school together every day to increase their Safety to School (Picture 6 in Fig 3). Table 3 depicts the implementation details of each strategy as well as the specific challenges the strategies are designed to address and their intended outcomes.

**Table 3:** SchoolLinks strategy description.

Strategy	Intended outcomes	Risk factors and issues addressed	Who is targeted?	When?	Where?	Implementation support by WCA	Materials
1. Meetings	Increase communication between home and school Mobilise participation for subsequent strategies.	Caregivers and teachers did not know each other. Communication between them was minimal; when it occurred, it focused mainly on school fees.	Caregivers and teachers.	Twice per term.	School.	Coordinate meeting logistics, procure materials, facilitate first Meeting.	Printed posters of visual depiction of SchoolLinks. Refreshments (drinks, biscuits).
2. Translation	Increase communication between home and school.	Language barrier between teachers and caregivers. Low attendance of parent-teacher meetings.	Volunteer community members.	At Meetings and Enrolment.	School.	Engagement of translator, briefing on Meeting agenda.	None.
3. Enrolment Week	Establish caregiver-teacher relationship. Increase teacher knowledge of child. Ensure child is enrolled in appropriate class with correct name. Increase caregiver knowledge of child’s school environment. Capture data for strategies 4–6.	Caregivers often did not know their child’s teacher, classmates, or which class the child was in. Children usually enrolled themselves, often fluctuating between AEP and mainstream school. Teachers lacked information on children’s needs and home contexts. Enrolment data, e.g., child’s name and date of birth, were often incorrect.	Caregivers, teachers, children.	First week of term.	School.	Creation of digital enrolment form, provision of half-day training to teachers. Printing data for school and district local government records.	Tablets (lent). Digital enrolment form (accessed through Kobo Toolbox).
4. Attendance monitoring and reward	Increase child motivation. Alleviate financial stress through (small) in-kind support.	Low school attendance. Low motivation of children and caregivers. Prohibitive cost of scholastic materials and soap.	Students and teachers.	Weekly monitoring, 4-week cycles for rewards, 2 cycles per term.	School.	Procurement of rewards.	Rewards (soap, exercise book & pen). Star charts (laminated, Velcro – reusable).
5. Homework Clubs	Increase caregiver knowledge. Student peer support and learning.	Homework not regularly completed. Lack of awareness about children’s schoolwork. Reluctance to go to school due to incomplete homework. Low academic improvement and consolidation of learning.	Students and volunteer caregivers.	Weekly, at the weekend.	Caregiver’s compound/ community space.	Assistance with grouping.	None.
6. Safety to school	Increase student safety on the way to school.	Absenteeism and truancy. Caregivers’ and children’s fear of harm on the route to school.	Students.	Weekday mornings.	On the route to school.	None.	None.

#### Acceptability.

Findings in this and the two subsequent sections quote the field notes taken during field-testing (see S3 Text for the compiled notes). Feedback from caregivers, school staff, and students indicated that the six strategies were well accepted, and the community-informed design was “*exciting*” and “*appreciated*”. Community leaders felt it was a “*wonderful programme*”. Aside from discussing SchoolLinks and the importance of caregivers’ engagement, caregivers valued the Meetings as a platform to discuss issues they felt pertinent. They provided a forum for ongoing consultation, mutual appreciation, and power-sharing between caregivers and school staff. For example, in the second Meeting caregivers presented the SchoolLinks strategies themselves and led the closing prayer. Translation was highly appreciated; one caregiver said that “*for once, she has attended a meeting where she has heard what [the school staff] are communicating and they are also able to hear her views*”. Initial concerns about finding volunteer translators proved unfounded, as the two identified considered it a “*honour*.”

Caregivers turned up for Enrolment week in “*surprisingly high*” numbers and demonstrated their commitment by patiently waiting for their turn with the teacher; “*even when they were asked to go home at lunch and break and return in the afternoon, they insisted on staying […] and getting the exercise concluded. Their resilience was humbling to the research team and teachers*”.

Attendance monitoring and rewards effectively motivated students, and caregivers deeply appreciated the soap; “*the whole community washed*.” The first time the rewards were distributed was a “*beautiful day*”, as “*[the children] who were to receive could not hold back their joy*” and even the teachers “*joined in the jubilations”*. After one cycle, 74% of children were rewarded; after the second cycle, this rose to 93%.

Caregivers appreciated the Homework Clubs as they “*helped to settle down children to open books over the weekend, something that used not to happen*” and “*children are getting to learn the importance of reading from home*”. Caregivers willingly volunteered as supervisors; when more were requested, 25 caregivers volunteered. PTA and SMC members offered support without prompting by visiting the Clubs at the weekend. Students also “*did not want to miss [them]*”.

Safety to School reassured caregivers; “*this is comforting to us as parents knowing that our children will reach school safely and return safely*”. Teachers also noted that children in P3 and AEP1 were the first to arrive at school, would clean the classrooms and schoolyard, and then sit waiting for the teacher.

#### Appropriateness & demand.

In a preparatory meeting, a community leader testified to his child dropping out of school and running away, and “*urged the leaders to work hand in hand with [WCA] to support these kinds of children to return to school*”, underscoring the demand for interventions like SchoolLinks. Caregiver engagement during Meetings, including asking questions and sharing opinions, indicated the strategy’s value in strengthening home–school communication. A teacher noted that *“[SchoolLinks] has bridged the communication gap that existed between the caregivers, community, and the school*” and that Translation supported “*mutual understanding*”. In one Meeting, a caregiver indicated that she only spoke Aringa, so one-to-one Translation was arranged to meet this demand.

Caregivers acknowledged the importance of attending Enrolment week with their children to ensure accurate names, date of birth, and grade placement, as well as show support to the school; to “*join hands with the teachers and school administrators and show support and love for education of our children*.” Both teachers and caregivers also acknowledged the importance of discussing specific needs and disabilities so that the *“the child will be helped”*. Caregivers who did not attend Enrolment week later asked to enrol their children so that their children would be included in the other SchoolLinks strategies. The choice of attendance rewards was considered appropriate as they were low-cost, locally sourced and alleviated (small) financial burden, as confirmed by a caregiver: “*the reason as to why children don’t attend regularly is because of dirty uniforms and parents can’t afford washing soap*”. The conditionality of rewards was also supported, as a teacher shared that some students register at multiple schools in the hope of receiving “*an immediate benefit*”.

Homework Clubs “*received tremendous support*” from caregivers, teachers, and children, increasing academic support at home and improving caregiver awareness of their children’s learning. One caregiver noted that they “*create unity among children and parents […] and grow stronger bonds*”. Demand was demonstrated by caregivers’ and children’s requests to teachers to give more homework, which the teachers obliged. Increased unity among children was also attributed to group modalities of Safety to School and the Homework Clubs; one teacher noted the case of a child who uses a wheelchair who managed to consistently come to school on time “*with the support of his friends in his Homework Club*”. Safety to School was well-received as a practical response to road safety and truancy concerns as well as improving students’ punctuality. Suggestions of ‘downstream’ positive effects were noted, such as children learning to read their names because of the star charts, and school staff using the Meetings to communicate changes to refugee education policy, which supports the appropriateness of designing an intervention from a systems perspective.

#### Feasibility and practicality.

The field test helped identify challenges and inform direct adaptations to the intervention design (see Table 4). A key learning was the importance of adapting to caregivers’ schedules, for example, avoiding activities during gardening times or market days. Adjusting the Meeting time from morning to afternoon increased caregivers’ attendance from 70% to 90%. To account for Ugandan teachers travelling home during holidays, Enrolment Week was moved to the first week of term. Bad weather and low digital literacy slowed enrolment, but it sped up as familiarity with the process increased. Similarly, Attendance Monitoring was initially time-consuming as teachers did not know all children by name or sight, and children took time to find their names on the star charts. It was more difficult for children who lived further away to participate in Homework Clubs and Safety to School, though most persevered. Another learning was the need for implementation flexibility, for example, allowing caregivers to coordinate Homework Club meeting times and locations between themselves, and for children to do the same for Safety to School.

**Table 4:** Field-test challenges and adaptations.

Strategy	Challenges	Adaptations
1. Meetings	Low initial caregiver attendance due to meeting time clashing with morning gardening schedules. Late arrival of participants delayed meetings.	Changed meeting time from morning to afternoon. Held additional meetings to increase reach and flexibility.
2. Translation	Initial difficulty identifying appropriate translators.	Gathered recommendations for translators from school administration and community leadership. Developed guidelines for translators.
3. Enrolment Week	Some caregivers and children arrived separately, or neighbours/friends were asked to register children. Teachers were initially slow to use tablets. Rain disrupted the process. School was unprepared for PTA fee collection. Some caregivers did not turn up.	Required both caregiver and child to proceed with registration. Enrolment extended by one week to capture those who did not show up in the first week. Manual records used as a backup and then digitised afterwards.
4. Attendance monitoring and rewards	Some students’ names were misspelled. Some students’ names were missing as they had not been enrolled.	Developed a data validation process to ensure correct spellings and any other data entry errors. Extended enrolment (as above). Updated star charts after second week of enrolment to include all names.
5. Homework Clubs	Some caregivers were concerned about how they would support learning due to their own lack of education. Teachers were concerned that group formation by caregivers would be impractical. Weather disruptions (rain). Bad roads affected access to some homes.	PTA/SMC members voluntarily supervised groups and provided support to caregivers. Teachers formed groups instead of caregivers. Caregivers were given autonomy to arrange the clubs as they wished. Children created group names.
6. Safety to School	Caregivers left early for gardening, so did not organize or accompany children. Some children feared crossing roads or lacked group support.	Children self-organized meeting points and peer support groups. Strategy proceeded without requiring caregiver accompaniment.

Teachers took on multiple roles which could risk overburdening them over time or at a larger scale. Support from the WCA implementers, especially at the start, was necessary and valuable but it will be important to determine the precise role and responsibilities of such external implementers in the future. Small indications of task-sharing, such as school management taking over facilitation of the Meetings and supporting caregivers in the Homework Clubs, suggest that the implementer role could be withdrawn over time to increase programme sustainability.

## Discussion

This paper describes the participatory and iterative development process of SchoolLinks, a multi-strategy intervention designed to increase school attendance and retention amongst primary school children in refugee settings. The research consisted of three phases (i) Group Model Building, (ii) respondent validation & consultation, and (iii) field-testing – in four refugee settlements across Uganda over 13 months. The final set of intervention strategies (1. Meetings, 2. Translation, 3. Enrolment week, 4. Attendance monitoring and reward, 5. Homework Clubs, and 6. Safety to School) were designed to have multiple positive, systemic effects on caregiver engagement, the home-school relationship, and children’s motivation. Participants expressed enthusiasm for SchoolLinks and considered it relevant and appropriate to the context, but gave substantial feedback to improve the feasibility of its implementation during respondent validation and field-testing.

The multiphase design process integrated participants’ input at multiple timepoints, thus grounding the design in cultural and contextual relevance, acceptability, and feasibility. This study adds to the small but growing evidence base for participatory intervention development in conflict-affected settings. The GMB structured workshop design facilitated participants’ understanding and contributions by sequentially introducing a new perspective to the data and discussion [62]. There was high value in engaging research assistants from the research contexts to put participants at ease, translate, and contribute their own insights [66]. Using storytelling for variable elicitation, and translation, increased the inclusivity of the workshops however the combination of both was time-consuming. More time and further adaptation of structured activities to low-literate and multi-lingual populations, for example, using pictures or symbols, would be beneficial in future use of this method.

### Comparison to risk and protective factors

This research demonstrates the value of using systems modelling to inform intervention development. The group model building and resultant causal map reflect many risk and protective factors identified in the literature. At the child level, the positive influence of friendships and peer support was highlighted and incorporated into the intervention design, specifically Homework Clubs and Safety to School. Children’s mental health was not discussed in detail but their motivation to attend school emerged in the causal map as a key influencing factor on attendance. This reflects findings on the importance of children’s intrinsic motivation, for example in Mozambique [67] and South Africa [68]. Motivation is implicit in much literature, e.g., on safety or bullying, but the association between children’s motivation and school attendance in refugee settings would benefit from deeper investigation.

At the household level, poverty was discussed as a strong risk factor for absenteeism and drop-out, aligning closely with findings from research in low-income and conflict settings [9,12,17]. The choice of soap, exercise books, and pens as Rewards was guided by these discussions, and their conditional nature aligns more consistent evidence for conditional over unconditional in-kind incentives to influence attendance [31]. Caregivers’ knowledge of children’s schooling, attitudes towards education, and degree of encouragement were identified as important protective factors if strengthened. These were influenced by perceptions of quality and safety at the school, the child’s learning outcomes, and their relationship with teachers and school staff. This supports evidence from low-resource contexts on the protective role of caregiver engagement [9,12,20], and raises the importance of further investigation of this within refugee contexts.

At the school level, the language barrier and safety to school were consistently mentioned in the GMBs as challenges to attendance, aligning with findings of research investigating caregiver engagement, school attendance, and children’s schooling experience in refugee contexts [24,26,69]. The relationship between teachers and caregivers emerged as a central factor in the causal map, as it was closely linked to the relationship between teachers and students and their motivation to come to school, and influenced by communication between teachers and caregivers (or lack thereof). The relationship and communication between caregivers and teachers were considered key leverage points as, when positive, they boost caregivers’ knowledge of and encouragement towards the child, as well as teachers’ motivation, knowledge of and empathy for their students. The importance of this relationship is recognised through many global initiatives promoting home-school partnerships, such as Epstein’s School-Family-Community Partnership Model [70], though investigation of its influence on attendance in refugee contexts remains underexplored.

### Design rationale

The SchoolLinks design draws on elements of other attendance interventions. First, the use of conditional incentives was noted to be effective by teachers. Second, increasing caregivers’ knowledge of their child’s schooling, with an emphasis on information exchange between the home and school rather than ‘sensitisation’ of caregivers, was appreciated by caregivers. Third, is the use of multiple strategies rather than a single intervention to address a complex issue, such as attendance [3,31,71]. Fourth, targeted intervention on safety and language barriers were demanded by participants, as observed in other refugee contexts [25,26,72,73]. Fifth, though the strategies are too overt to be considered ‘nudges’, the design draws on the premise of using multiple prompts or incentives to influence greater behaviour change [74].

Much research and programming on caregiver engagement and home-school partnerships is centred on Epstein’s [75] model, which describes six types of parental involvement in education: (1) parenting (e.g., waking and feeding the child before school), (2) home-school communication, (3) parental volunteering, (4) home learning support, (5) parent involvement in decision-making, and (6) community collaboration. We observe several similarities between Epstein’s model and the SchoolLinks design. For instance, encouraging caregivers to enrol their child in school rather than letting them enrol themselves promotes parenting. Additionally, increasing home-school communication is central to the SchoolLinks design, promoted through Meetings, Translation, and Enrolment Week. Parent volunteering and home learning support featured in the Homework Clubs as well as Meetings, where caregivers chose to prioritise their attendance of the meetings over other obligations and engaged in discussions with teachers on ways to support learning at home. Caregivers were involved in decision-making about the implementation of Homework Clubs and Safety to School, in terms of when, where, and who to conduct them, however their increased involvement in decision-making about school governance is not an explicit part of the SchoolLinks design. Lastly, community involvement was demonstrated through community leaders’ vocal support for SchoolLinks and importance of education, faith leaders making places of worship available for Homework Clubs, and caregivers’ willingness to host Homework Clubs. Similarly to other evidence generated on caregiver engagement amongst refugees [26], our research highlights the additional provisions required for effective implementation of Epstein’s model, including translation for multilingual communities, flexibility in implementation schedules, and socioeconomic support.

### Gaps and further development

Gender- and disability-targeted strategies were not incorporated into the design, however we included the questions about disability and functioning in the enrolment form so that the teacher and school had increased awareness of children’s disability status, and two questions for the caregiver to be able to highlight additional needs of their children. Caregiver mental health, family wellbeing, income generation, and poverty were also beyond the scope of this intervention, underscoring the need for holistic, multisectoral intervention to have positive, systemic effect in conflict and low-resource settings [56]. While volunteer roles were accepted, and additional support voluntarily given by the PTA and SMC is promising, longer implementation would reveal if this will be sustainable. Implementation materials, including rewards and meeting refreshments, are small unit costs but may be prohibitive at scale and may lose their motivating power if given repeatedly. It is also important to recognise that the selection of strategies was shaped in part by WCA’s organisational mandate. While this was appropriate for the context of this intervention development, S1 Text (full list of action ideas) demonstrates the much broader scope of initiatives proposed by participants to promote school attendance and caregiver engagement.

The promising findings of this study warrant further research to validate the mechanisms of action and evaluate the effectiveness of SchoolLinks [10]. It would also be beneficial to assess SchoolLinks’ applicability in a different refugee context and potentially non-refugee contexts too. Considering the stagnation in government funding and reduction humanitarian funding for education [76,77], evaluation of the effects of SchoolLinks on learning outcomes would shed light on its potential to increase the cost-efficiency of standard education.

### Limitations

This study had several limitations. First, omitting children and adolescents from the GMBs precluded their perspectives, which to some extent limits the validity of the causal map [78]. This was due to a combination of budgetary constraints, child protection considerations, and logistical parameters; however, we validated the model in subsequent work with adolescents. Second, it would have been beneficial to purposively include caregivers of children facing additional barriers to attendance (e.g., disability, longest distances). Third, power dynamics and social desirability between caregivers, teachers, partners, and WCA staff could have influenced input and feedback. We tried to limit social desirability by emphasising to enumerators and participants our need for genuine input and feedback, and that there were no right or wrong answers. We also encouraged participants to consider others’ perspectives (e.g., “what might your friends or neighbours suggest to improve this strategy?”) to lessen the perceived risk of giving ‘negative’ feedback. Fourth, the implementation was too short to measure student retention or dropout. We also acknowledge that the positive influence reported on attendance may be the effect of novelty and not sustained during longer implementation. Fifth, there was a risk of an “implementer effect”, where the thorough follow-up of the implementing staff member may have positively influenced (but therefore confounded) the uptake of the intervention.

## Conclusion

SchoolLinks is a promising intervention to increase students’ school attendance and retention in refugee settings in Uganda; the mechanism of change for SchoolLinks runs primarily through strengthening caregiver’s engagement in children’s education and relationship with the school. The participatory design methods were key to understanding the contextual challenges and opportunities, leading to acceptable and appropriate intervention strategies. SchoolLinks responds to a gap in initiatives aimed at increasing school attendance, key to increasing children’s learning outcomes and with the potential to contribute to improving the cost-effectiveness of learning programmes and other school-based interventions. The intervention will next undergo a proof-of-concept study, to evaluate the effect on attendance, retention and caregiver engagement, and value for money.

## Supporting information

S1 Table Variables and categorisations. All variables generated in the group model building workshops with their categorisations.
(PDF)

S2 Table Causal links. Causal links extracted from the connection circles, which were developed in the group model building workshops.
(PDF)

S1 Fig Causal loop diagrams. Causal loop diagrams extracted from the connection circles, per category and location. Blue arrows indicate positive polarity, and red arrows indicate negative polarity.
(PDF)

S1 Text Action ideas. All action ideas generated from the group model building workshops, per location.
(PDF)

S2 Text Validation and consultation workshop notes. Combined notes from the four validation and consultation workshops.
(PDF)

S3 Text Field-test notes. Compiled notes from the field-testing of SchoolLinks.
(PDF)

S1 File Inclusivity in global research questionnaire. (PDF)
