# Supplementary material for: Increasing school attendance and retention in refugee settings: Participatory development of the SchoolLinks intervention
Source: PLoS One. 2026 Aug 31;21(8):e0357215. doi: 10.1371/journal.pone.0357215 (PMC13528952; doi:10.1371/journal.pone.0357215)
Supplement: Supplementary file 1 [file pone.0357215.s001.pdf]

S1 Table. Variables and their categorisations from the group model building workshops

Settlement	Category	Variable
Settlement 1	Caregiver	Ability of movement outside home
Settlement 1	Caregiver	Ability to work (non-farming)
Settlement 1	Caregiver	Access to jobs
Settlement 1	Caregiver	Availability of land for production
Settlement 1	Caregiver	Availability of scholastic materials
Settlement 1	Caregiver	Caregiver alcoholism
Settlement 1	Caregiver	Caregiver illiteracy
Settlement 1	Caregiver	Caregiver motivation towards child's education
Settlement 1	Caregiver	Caregiver trauma
Settlement 1	Caregiver	Domestic violence
Settlement 1	Caregiver	Family breakdown
Settlement 1	Caregiver	Family prioritisation of farming
Settlement 1	Caregiver	Hopelessness
Settlement 1	Caregiver	Household Financial Spending
Settlement 1	Caregiver	Number of children in a home
Settlement 1	Caregiver	Parent health and wellbeing
Settlement 1	Caregiver	Possibility of repatriation
Settlement 1	Caregiver	Poverty
Settlement 1	Caregiver	Provision of basic needs (Soap and Vaseline)
Settlement 1	Caregiver	Provision of food at home
Settlement 1	Caregiver	Provision of School fees
Settlement 1	Caregiver	Single parenthood
Settlement 1	Child	Age of the child
Settlement 1	Child	Child Attending School
Settlement 1	Child	Child education performance
Settlement 1	Child	Child engagement in household activities
Settlement 1	Child	Child headed families
Settlement 1	Child	Child health and wellbeing
Settlement 1	Child	Child motivation
Settlement 1	Child	Corporal punishment
Settlement 1	Child	Distance to school
Settlement 1	Child	Early marriage
Settlement 1	Child	Early pregnancies
Settlement 1	Child	Inferiority complex
Settlement 1	Child	Lockdown
Settlement 1	Child	Recognition of qualifications across different countries
Settlement 1	Child	School dropout
Settlement 1	Child	Selective provision of scholastic materials
Settlement 1	Communication	Adherence to time during meetings

Settlement 1	Communication	Caregiver-teacher relationships
Settlement 1	Communication	Communication between school and caregivers
Settlement 1	Communication	Language barrier
Settlement 1	Communication	Parent being praised for valuing their children's education
Settlement 1	Communication	Teacher feedback to caregivers on child performance
Settlement 1	Communication	Teacher guidance to the caregivers
Settlement 1	Knowledge and Attitudes	Attitudes of caregivers towards education
Settlement 1	Knowledge and Attitudes	Caregivers' education level
Settlement 1	Knowledge and Attitudes	Caregivers' value towards education
Settlement 1	Knowledge and Attitudes	Hope for further studies
Settlement 1	Knowledge and Attitudes	Inspiration of role models
Settlement 1	School Support	Facilitating caregivers during career days
Settlement 1	School Support	Home visits by the teachers
Settlement 1	School Support	Motivation of caregivers
Settlement 1	School Support	Provision of lunch and drinking water at school
Settlement 1	School Support	Teacher engagement
Settlement 2	Caregivers	Ability to borrow money
Settlement 2	Caregivers	Ability to buy lamps and kerosene
Settlement 2	Caregivers	Ability to pack lunch
Settlement 2	Caregivers	Accompanying a child to school
Settlement 2	Caregivers	Age of the caregiver
Settlement 2	Caregivers	Alcoholism
Settlement 2	Caregivers	Availability of food at home
Settlement 2	Caregivers	Availability of land for production
Settlement 2	Caregivers	Availability of time for children
Settlement 2	Caregivers	Bureaucracy of handling domestic violence
Settlement 2	Caregivers	Caregiver homework support
Settlement 2	Caregivers	Caregiver prioritisation to children in upper classes
Settlement 2	Caregivers	Cash relief
Settlement 2	Caregivers	Child taken to grandparents' home
Settlement 2	Caregivers	Child-caregiver relationships
Settlement 2	Caregivers	Child-headed families
Settlement 2	Caregivers	Domestic violence
Settlement 2	Caregivers	Employment opportunities
Settlement 2	Caregivers	Family breakdown
Settlement 2	Caregivers	Family size
Settlement 2	Caregivers	Financial burden
Settlement 2	Caregivers	Ghost recipient
Settlement 2	Caregivers	Household financial spending
Settlement 2	Caregivers	Job search
Settlement 2	Caregivers	NGO support
Settlement 2	Caregivers	Number of children in a home
Settlement 2	Caregivers	Overwhelmed foster caregivers

Settlement 2	Caregivers	Physical disability
Settlement 2	Caregivers	Police corruption
Settlement 2	Caregivers	Poverty
Settlement 2	Caregivers	Provision of basic needs
Settlement 2	Caregivers	Provision of scholastic materials
Settlement 2	Caregivers	Provision of school fees
Settlement 2	Caregivers	Psychological trauma of family members
Settlement 2	Caregivers	Self-neglect
Settlement 2	Caregivers	Sickness of caregiver
Settlement 2	Caregivers	Single parenthood
Settlement 2	Caregivers	Village savings and loan associations
Settlement 2	Children	Ability to pay for transport
Settlement 2	Children	Aesthetic bleaching by a child
Settlement 2	Children	Boys working in mines quarry and herding
Settlement 2	Children	Child academic performance
Settlement 2	Children	Child attending School
Settlement 2	Children	Child discipline
Settlement 2	Children	Child engaged in domestic work
Settlement 2	Children	Child literacy
Settlement 2	Children	Child motivation to learn
Settlement 2	Children	Concentration in class
Settlement 2	Children	COVID-19 lockdown
Settlement 2	Children	Distance to School
Settlement 2	Children	Distraction of boys
Settlement 2	Children	Dropout of School
Settlement 2	Children	Early marriages
Settlement 2	Children	Early pregnancies
Settlement 2	Children	Female teachers' dress code perceived as inappropriate
Settlement 2	Children	Hunger
Settlement 2	Children	Peer pressure
Settlement 2	Children	Sickness of a child
Settlement 2	Children	Stealing of foodstuff
Settlement 2	Children	Teenager demands
Settlement 2	Communication	Caregiver engagement in school activities and meetings
Settlement 2	Communication	Caregivers' value for education.
Settlement 2	Communication	Child-teacher relationships
Settlement 2	Communication	Invitation to school meetings
Settlement 2	Communication	Language barrier between caregiver and teacher
Settlement 2	Communication	Teacher guidance to the child
Settlement 2	Communication	Teacher personal support to vulnerable children
Settlement 2	Communication	Teacher-caregiver communication
Settlement 2	Communication	Teacher-caregiver relationships
Settlement 2	Knowledge and Attitudes	Caregivers' attitude towards education

Settlement 2	Knowledge and Attitudes	Caregivers' level of education
Settlement 2	Knowledge and Attitudes	Caregivers' motivation to support child
Settlement 2	Knowledge and Attitudes	Caregivers' value for education
Settlement 2	Knowledge and Attitudes	Follow-up of child's progress
Settlement 2	School Support	Accountability from School
Settlement 2	School Support	Attitude of caregivers towards School
Settlement 2	School Support	Class size
Settlement 2	School Support	Quality of teaching
Settlement 2	School Support	Regular attendance
Settlement 2	School Support	School performance
Settlement 2	School Support	Security at School
Settlement 2	School Support	Termly School fees
Settlement 3	Caregiver	Alcoholism of Caregiver
Settlement 3	Caregiver	Availability of food in the home
Settlement 3	Caregiver	Availability of jobs
Settlement 3	Caregiver	Caregiver attitude towards the child
Settlement 3	Caregiver	Caregiver availability of time with children
Settlement 3	Caregiver	Caregiver motivation to support a child
Settlement 3	Caregiver	Caregiver pressure to adhere to cultural norms
Settlement 3	Caregiver	Caregiver supporting child with homework
Settlement 3	Caregiver	Caregiver’s will to accompany a child to school
Settlement 3	Caregiver	Child respect for caregivers
Settlement 3	Caregiver	Child-caregiver relationship
Settlement 3	Caregiver	COVID-19 pandemic lockdown
Settlement 3	Caregiver	Family instability
Settlement 3	Caregiver	Happiness of caregivers
Settlement 3	Caregiver	Household financial spending
Settlement 3	Caregiver	Illiteracy of caregivers
Settlement 3	Caregiver	Negative peer influence between caregivers
Settlement 3	Caregiver	Number of children
Settlement 3	Caregiver	Payment of school fees
Settlement 3	Caregiver	Possibility of repatriation
Settlement 3	Caregiver	Poverty
Settlement 3	Caregiver	Provision of scholastic material
Settlement 3	Caregiver	Relationship between husband and wife
Settlement 3	Caregiver	Sale of agricultural produce goats and chicken
Settlement 3	Caregiver	Support from husband
Settlement 3	Caregiver	Traffic on the road
Settlement 3	Children	Access to sanitary pads
Settlement 3	Children	Bad weather
Settlement 3	Children	Behaviour of siblings
Settlement 3	Children	Child academic performance
Settlement 3	Children	Child Attending School

Settlement 3	Children	Child having good friends
Settlement 3	Children	Child motivation to learn
Settlement 3	Children	Child's disability
Settlement 3	Children	Child's discipline
Settlement 3	Children	Child's health and wellbeing
Settlement 3	Children	Concentration in class
Settlement 3	Children	Corporal punishments
Settlement 3	Children	COVID-19 Lockdown
Settlement 3	Children	Distance to school
Settlement 3	Children	Early marriage
Settlement 3	Children	Early pregnancy
Settlement 3	Children	Engagement in house chores
Settlement 3	Children	Engaging in agricultural activities
Settlement 3	Children	Fear of kidnap and rape
Settlement 3	Children	Hunger
Settlement 3	Children	Loss of parents
Settlement 3	Children	Morale of caregivers
Settlement 3	Children	Peer pressure
Settlement 3	Children	Repeating class
Settlement 3	Children	School dropout
Settlement 3	Children	War
Settlement 3	Communication	Attendance of class day meetings
Settlement 3	Communication	Caregivers follow up with the teacher about child performance.
Settlement 3	Communication	Language barrier
Settlement 3	Communication	Parents speaking the same language
Settlement 3	Communication	Teacher feedback to caregivers about child performance
Settlement 3	Communication	Teacher-Caregiver relationship
Settlement 3	Communication	Teachers and caregivers give consistent support to the child
Settlement 3	Knowledge and Attitudes	Caregivers' attitude towards education
Settlement 3	Knowledge and Attitudes	Caregivers follow up with the teacher about child performance
Settlement 3	Knowledge and Attitudes	Caregivers' value of education
Settlement 3	Knowledge and Attitudes	Inspiration of role models e.g. church leaders
Settlement 3	School Support	Competent teachers
Settlement 3	School Support	Learning environment
Settlement 3	School Support	Motivation to teach
Settlement 3	School Support	Teacher attitude towards learners
Settlement 4	Caregivers	Alcoholism
Settlement 4	Caregivers	Availability of food
Settlement 4	Caregivers	Availability of time with children
Settlement 4	Caregivers	Bad weather
Settlement 4	Caregivers	Brewing alcohol
Settlement 4	Caregivers	Business opportunities for caregivers
Settlement 4	Caregivers	Caregivers separated across countries

Settlement 4	Caregivers	Child-parent relationships
Settlement 4	Caregivers	COVID-19 lockdown
Settlement 4	Caregivers	Disability of caregivers
Settlement 4	Caregivers	Domestic violence
Settlement 4	Caregivers	Engagement in Agriculture
Settlement 4	Caregivers	Family breakdown
Settlement 4	Caregivers	Food SACCO
Settlement 4	Caregivers	Homework support
Settlement 4	Caregivers	Household financial spending
Settlement 4	Caregivers	Number of children in a home
Settlement 4	Caregivers	Poverty
Settlement 4	Caregivers	Provision of basic needs like soap etc
Settlement 4	Caregivers	Provision of scholastic materials
Settlement 4	Caregivers	Provision of school and exam fees
Settlement 4	Caregivers	Responsible caregiver
Settlement 4	Caregivers	Single parenthood
Settlement 4	Children	Ability to learn
Settlement 4	Children	AEP students return to mainstream
Settlement 4	Children	Bad weather.
Settlement 4	Children	Child academic performance
Settlement 4	Children	Child attending school
Settlement 4	Children	Child discipline
Settlement 4	Children	Child engagement in domestic work
Settlement 4	Children	Child headed family
Settlement 4	Children	Child health and wellbeing
Settlement 4	Children	Child motivation to learn
Settlement 4	Children	Child usefulness to family and community
Settlement 4	Children	Children enrol in AEP
Settlement 4	Children	Child's fear of going to school
Settlement 4	Children	Child's fear of rape and kidnapping
Settlement 4	Children	Child's hope to further studies
Settlement 4	Children	Child's inferiority complex
Settlement 4	Children	Child's urge to be accompanied
Settlement 4	Children	Concentration in class
Settlement 4	Children	Corporal punishment
Settlement 4	Children	Distance to School
Settlement 4	Children	Dropout of School
Settlement 4	Children	Drug abuse
Settlement 4	Children	Early marriage
Settlement 4	Children	Early pregnancy
Settlement 4	Children	Having school and basic needs
Settlement 4	Children	Hunger
Settlement 4	Children	Orphans unable to support themselves

Settlement 4	Children	Peer pressure and bullying
Settlement 4	Children	Support to teenage mothers
Settlement 4	Communication	Caregiver - teacher communication
Settlement 4	Communication	Caregiver desire to engage in school activities
Settlement 4	Communication	Caregiver visiting school on their own initiative
Settlement 4	Communication	Caregiver-teacher relationships
Settlement 4	Communication	Invitation of caregivers to School
Settlement 4	Communication	Language barrier
Settlement 4	Communication	Regular meetings between caregiver and teachers
Settlement 4	Communication	Teacher feedback to caregivers
Settlement 4	Knowledge and Attitudes	Caregiver motivation to support children's learning
Settlement 4	Knowledge and Attitudes	Caregivers' attitude towards education
Settlement 4	Knowledge and Attitudes	Caregivers follow up with the teacher about child performance
Settlement 4	Knowledge and Attitudes	Caregiver's level of education
Settlement 4	Knowledge and Attitudes	Caregiver's value of education
Settlement 4	Knowledge and Attitudes	Child motivation to learn.
Settlement 4	Knowledge and Attitudes	Inspiration from other children as role models
Settlement 4	School Support	Attitude of caregivers towards the school administration
Settlement 4	School Support	Class size
Settlement 4	School Support	Learning environment
Settlement 4	School Support	Number of teachers
Settlement 4	School Support	Performance of teacher on duty
Settlement 4	School Support	Quality of facilities at school
Settlement 4	School Support	Quality of teaching
Settlement 4	School Support	School population
Settlement 4	School Support	Security at School
