# Supplementary material for: Increasing school attendance and retention in refugee settings: Participatory development of the SchoolLinks intervention
Source: PLoS One. 2026 Aug 31;21(8):e0357215. doi: 10.1371/journal.pone.0357215 (PMC13528952; doi:10.1371/journal.pone.0357215)
Supplement: Supplementary file 2 [file pone.0357215.s002.pdf]

S2 Table. Causal links extracted from connection circles in the group model building workshops.

Contents

Settlement 1 1
Settlement 2 4
Settlement 3 9
Settlement 4 13

Settlement 1

Variable 1	Polarity	Variable 2	Theme
Availability of land for production	Increases	Provision of food at home	Caregiver
Household Financial Spending	Increases	Provision of food at home	Caregiver
Provision of food at home	Increases	Household Financial Spending	Caregiver
Household Financial Spending	Increases	Provision of School fees	Caregiver
Access to jobs	Increases	Household Financial Spending	Caregiver
Provision of School fees	Decreases	Household Financial Spending	Caregiver
Access to jobs	Increases	Ability to work (non-farming)	Caregiver
Ability to work (non-farming)	Decreases	Family prioritisation of farming	Caregiver
Availability of scholastic materials	Decreases	Household Financial Spending	Caregiver
Household Financial Spending	Increases	Provision of basic needs (Soap and Vaseline)	Caregiver
Provision of basic needs (Soap and Vaseline)	Decreases	Household Financial Spending	Caregiver
Parent health and wellbeing	Increases	Household Financial Spending	Caregiver
Number of children in a home	Decreases	Provision of food at home	Caregiver
Family breakdown	Increases	Caregiver trauma	Caregiver
Caregiver trauma	Increases	Caregiver alcoholism	Caregiver
Family breakdown	Increases	Single parenthood	Caregiver
Access to jobs	Decreases	Hopelessness	Caregiver
Possibility of repatriation	Increases	Hopelessness	Caregiver
Ability to work (non-farming)	Decreases	Hopelessness	Caregiver
Ability of movement outside home	Increases	Access to jobs	Caregiver
Caregiver illiteracy	Increases	Hopelessness	Caregiver
Caregiver illiteracy	Decreases	Access to jobs	Caregiver
Caregiver illiteracy	Increases	Family prioritisation of farming	Caregiver
Household Financial Spending	Increases	Caregiver alcoholism	Caregiver
Caregiver alcoholism	Decreases	Household Financial Spending	Caregiver
Caregiver motivation towards child's education	Decreases	Family prioritisation of farming	Caregiver

Caregiver alcoholism	Increases	Poverty	Caregiver
Access to jobs	Decreases	Poverty	Caregiver
Single parenthood	Decreases	Ability to work (non-farming)	Caregiver
Caregiver alcoholism	Increases	Domestic violence	Caregiver
Domestic violence	Increases	Family breakdown	Caregiver
Household Financial Spending	Decreases	Poverty	Caregiver
Parent health and wellbeing	Increases	Access to jobs	Caregiver
Ability of movement outside home	Decreases	Ability to work (non-farming)	Caregiver
Parent health and wellbeing	Decreases	Ability of movement outside home	Caregiver
Single parenthood	Decreases	Ability of movement outside home	Caregiver
Number of children in a home	Decreases	Household Financial Spending	Caregiver
Number of children in a home	Decreases	Ability of movement outside home	Caregiver
Number of children in a home	Decreases	Availability of scholastic materials	Caregiver
Number of children in a home	Decreases	Provision of School fees	Caregiver
Number of children in a home	Increases	Family prioritisation of farming	Caregiver
Possibility of repatriation	Decreases	Caregiver motivation towards child's education	Caregiver
Poverty	Increases	Domestic violence	Caregiver
Family prioritisation of farming	Increases	Provision of food at home	Caregiver
Access to jobs	Decreases	Family prioritisation of farming	Caregiver
Single parenthood	Decreases	Household Financial Spending	Caregiver
Household Financial Spending	Increases	Availability of scholastic materials	Caregiver
Number of children in a home	Decreases	Provision of basic needs (Soap and Vaseline)	Caregiver
Caregiver illiteracy	Decreases	Caregiver motivation towards child's education	Caregiver
Child motivation	Increases	Child education performance	Children
Child education performance	Increases	Child motivation	Children
Child motivation	Decreases	Early pregnancies	Children
Early pregnancies	Decreases	Child motivation	Children
Early marriage	Decreases	Child motivation	Children
Child motivation	Decreases	Early marriage	Children
Child health and wellbeing	Increases	Child education performance	Children
Child health and wellbeing	Increases	Child motivation	Children
Early pregnancies	Increases	Inferiority complex	Children
Lockdown	Increases	Early pregnancies	Children
Age of the child	Increases	Inferiority complex	Children
Age of the child	Increases	Early marriage	Children
Recognition of qualifications across different countries	Increases	Child motivation	Children
Child engagement in household activities	Decreases	Child education performance	Children
Child headed families	Increases	Child engagement in household activities	Children
Distance to school	Decreases	Child motivation	Children
Child education performance	Decreases	Inferiority complex	Children

Inferiority complex	Decreases	Child education performance	Children
Early marriage	Increases	Inferiority complex	Children
Selective provision of scholastic materials	Increases	Child motivation	Children
Early pregnancies	Increases	Early marriage	Children
Corporal punishment	Decreases	Child Attending School	Children
Child Attending School	Decreases	Early pregnancies	Children
Corporal punishment	Increases	School dropout	Children
School dropout	Increases	Child engagement in household activities	Children
Early pregnancies	Decreases	Child Attending School	Children
Early marriage	Increases	School dropout	Children
Child Attending School	Decreases	Child engagement in household activities	Children
Lockdown	Increases	Child engagement in household activities	Children
School dropout	Increases	Early marriage	Children
School dropout	Increases	Early pregnancies	Children
Early pregnancies	Increases	School dropout	Children
Inferiority complex	Decreases	Child motivation	Children
Adherence to time during meetings	Increases	Communication between school and caregivers	Communication
Communication between school and caregivers	Increases	Adherence to time during meetings	Communication
Parent being praised for valuing their children's education	Increases	Caregiver-teacher relationships	Communication
Caregiver-teacher relationships	Increases	Parent being praised for valuing their children's education	Communication
Language barrier	Decreases	Parent being praised for valuing their children's education	Communication
Language barrier	Decreases	Teacher feedback to caregivers on child performance	Communication
Language barrier	Decreases	Teacher guidance to the caregivers	Communication
Language barrier	Decreases	Communication between school and caregivers	Communication
Caregiver-teacher relationships	Increases	Teacher feedback to caregivers on child performance	Communication
Teacher feedback to caregivers on child performance	Increases	Caregiver-teacher relationships	Communication
Teacher feedback to caregivers on child performance	Increases	Communication between school and caregivers	Communication
Communication between school and caregivers	Increases	Teacher feedback to caregivers on child performance	Communication
Teacher feedback to caregivers on child performance	Increases	Teacher guidance to the caregivers	Communication
Teacher guidance to the caregivers	Increases	Caregiver-teacher relationships	Communication
Caregiver-teacher relationships	Increases	Teacher guidance to the caregivers	Communication
Caregiver-teacher relationships	Increases	Communication between school and caregivers	Communication
Communication between school and caregivers	Increases	Caregiver-teacher relationships	Communication
Language barrier	Decreases	Caregiver-teacher relationships	Communication

Teacher guidance to the caregivers	Increases	Communication between school and caregivers	Communication
Adherence to time during meetings	Increases	Teacher feedback to caregivers on child performance	Communication
Adherence to time during meetings	Increases	Caregiver-teacher relationships	Communication
Caregivers' education level	Increases	Caregivers' value towards education	Knowledge and Attitudes
Caregivers' education level	Increases	Hope for further studies	Knowledge and Attitudes
Caregivers' value towards education	Increases	Hope for further studies	Knowledge and Attitudes
Caregivers' education level	Increases	Attitudes of caregivers towards education	Knowledge and Attitudes
Hope for further studies	Increases	Attitudes of caregivers towards education	Knowledge and Attitudes
Attitudes of caregivers towards education	Increases	Hope for further studies	Knowledge and Attitudes
Inspiration of role models	Increases	Hope for further studies	Knowledge and Attitudes
Inspiration of role models	Increases	Attitudes of caregivers towards education	Knowledge and Attitudes
Caregivers' value towards education	Increases	Attitudes of caregivers towards education	Knowledge and Attitudes
Teacher engagement	Increases	Home visits by the teachers	School Support
Provision of lunch and drinking water at school	Increases	Facilitating caregivers during career days	School Support
Home visits by the teachers	Increases	Motivation of caregivers	School Support
Teacher engagement	Increases	Motivation of caregivers	School Support
Motivation of caregivers	Increases	Teacher engagement	School Support
Facilitating caregivers during career days	Increases	Motivation of caregivers	School Support
Provision of lunch and drinking water at school	Increases	Motivation of caregivers	School Support

Settlement 2

**Variable 1**	**Polarity**	**Variable 2**	**Theme**
Distance to School	Decreases	Child attending School	Children
Early marriages	Decreases	Child attending School	Children
Early pregnancies	Decreases	Child attending School	Children
Child motivation to learn	Increases	Child attending School	Children
Boys working in mines quarry and herding	Decreases	Child attending School	Children
Dropout of School	Increases	Early marriages	Children
Early marriages	Increases	Dropout of School	Children
Peer pressure	Increases	Early marriages	Children
Early pregnancies	Increases	Early marriages	Children
Child academic performance	Increases	Child motivation to learn	Children
Child motivation to learn	Increases	Child academic performance	Children
Child discipline	Increases	Child academic performance	Children
Child academic performance	Increases	Child discipline	Children

Distraction of boys	Decreases	Child motivation to learn	Children
Boys working in mines quarry and herding	Decreases	Child motivation to learn	Children
Dropout of School	Increases	Early pregnancies	Children
Early pregnancies	Increases	Dropout of School	Children
Peer pressure	Increases	Stealing of foodstuff	Children
Child engaged in domestic work	Decreases	Child academic performance	Children
Child discipline	Decreases	Stealing of foodstuff	Children
Peer pressure	Increases	Aesthetic bleaching by a child	Children
Hunger	Decreases	Concentration in class	Children
Hunger	Increases	Stealing of foodstuff	Children
Ability to pay for transport	Increases	Child attending School	Children
Female teachers' dress code perceived as inappropriate	Increases	Distraction of boys	Children
Peer pressure	Increases	Early pregnancies	Children
Hunger	Decreases	Child motivation to learn	Children
Teenager demands	Increases	Early pregnancies	Children
Child literacy	Increases	Child motivation to learn	Children
Child literacy	Increases	Child academic performance	Children
Child academic performance	Increases	Child literacy	Children
Child discipline	Decreases	Aesthetic bleaching by a child	Children
Child motivation to learn	Decreases	Dropout of School	Children
Early pregnancies	Decreases	Child motivation to learn	Children
Sickness of a child	Decreases	Child motivation to learn	Children
COVID-19 lockdown	Increases	Early pregnancies	Children
COVID-19 lockdown	Decreases	Child academic performance	Children
Hunger	Decreases	Child attending School	Children
Child attending School	Increases	Child academic performance	Children
Concentration in class	Increases	Child attending School	Children
Teenager demands	Increases	Peer pressure	Children
Distance to School	Decreases	Child motivation to learn	Children
Distance to School	Decreases	Ability to pay for transport	Children
Sickness of a child	Decreases	Child attending School	Children
Child engaged in domestic work	Decreases	Child attending School	Children
Language barrier between caregiver and teacher	Decreases	Teacher-caregiver relationships	Communication
Language barrier between caregiver and teacher	Decreases	Teacher-caregiver communication	Communication
Language barrier between caregiver and teacher	Decreases	Caregiver engagement in school activities and meetings	Communication
Teacher-caregiver relationships	Increases	Caregiver engagement in school activities and meetings	Communication
Caregiver engagement in school activities and meetings	Increases	Teacher-caregiver relationships	Communication
Teacher-caregiver communication	Increases	Caregiver engagement in school activities and meetings	Communication

Caregiver engagement in school activities and meetings	Increases	Teacher-caregiver communication	Communication
Caregivers' value for education.	Increases	Teacher-caregiver relationships	Communication
Caregivers' value for education.	Increases	Caregiver engagement in school activities and meetings	Communication
Teacher personal support to vulnerable children	Increases	Child-teacher relationships	Communication
Child-teacher relationships	Increases	Teacher personal support to vulnerable children	Communication
Teacher guidance to the child	Increases	Teacher personal support to vulnerable children	Communication
Teacher personal support to vulnerable children	Increases	Teacher guidance to the child	Communication
Caregiver engagement in school activities and meetings	Increases	Invitation to school meetings	Communication
Invitation to school meetings	Increases	Caregiver engagement in school activities and meetings	Communication
Teacher-caregiver communication	Increases	Invitation to school meetings	Communication
Invitation to school meetings	Increases	Teacher-caregiver communication	Communication
Teacher guidance to the child	Increases	Child-teacher relationships	Communication
Child-teacher relationships	Increases	Teacher guidance to the child	Communication
Language barrier between caregiver and teacher	Decreases	Caregivers' value for education.	Communication
Teacher guidance to the child	Increases	Teacher-caregiver relationships	Communication
Teacher-caregiver relationships	Increases	Teacher guidance to the child	Communication
Invitation to school meetings	Increases	Teacher-caregiver relationships	Communication
Sickness of caregiver	Increases	Poverty	Caregiver
Poverty	Increases	Domestic violence	Caregiver
Poverty	Increases	Job search	Caregiver
Domestic violence	Increases	Family breakdown	Caregiver
Alcoholism	Increases	Domestic violence	Caregiver
Alcoholism	Decreases	Caregiver homework support	Caregiver
Alcoholism	Decreases	Child-caregiver relationships	Caregiver
Caregiver homework support	Increases	Child-caregiver relationships	Caregiver
Alcoholism	Increases	Self-neglect	Caregiver
Self-neglect	Increases	Alcoholism	Caregiver
Child-headed families	Increases	Overwhelmed foster caregivers	Caregiver
Alcoholism	Decreases	Availability of time for children	Caregiver
Poverty	Decreases	Availability of time for children	Caregiver
Job search	Increases	Employment opportunities	Caregiver
Village savings and loan associations	Increases	Ability to borrow money	Caregiver
Number of children in a home	Increases	Family size	Caregiver
Family size	Increases	Overwhelmed foster caregivers	Caregiver
Availability of land for production	Increases	Availability of food at home	Caregiver
Availability of food at home	Increases	Ability to pack lunch	Caregiver
Ability to pack lunch	Decreases	Availability of food at home	Caregiver

Cash relief	Decreases	Financial burden	Caregiver
Overwhelmed foster caregivers	Increases	Financial burden	Caregiver
Provision of school fees	Decreases	Household financial spending	Caregiver
Household financial spending	Increases	Provision of school fees	Caregiver
Household financial spending	Increases	Provision of scholastic materials	Caregiver
Provision of scholastic materials	Decreases	Household financial spending	Caregiver
Employment opportunities	Increases	Household financial spending	Caregiver
Number of children in a home	Decreases	Provision of school fees	Caregiver
Ability to borrow money	Increases	Household financial spending	Caregiver
Sickness of caregiver	Decreases	Household financial spending	Caregiver
Household financial spending	Increases	Availability of food at home	Caregiver
Number of children in a home	Increases	Caregiver prioritisation to children in upper classes	Caregiver
Ghost recipient	Decreases	Cash relief	Caregiver
Family breakdown	Increases	Single parenthood	Caregiver
Household financial spending	Increases	Provision of basic needs	Caregiver
Provision of basic needs	Decreases	Household financial spending	Caregiver
Single parenthood	Decreases	Availability of time for children	Caregiver
Psychological trauma of family members	Increases	Alcoholism	Caregiver
Alcoholism	Increases	Psychological trauma of family members	Caregiver
Poverty	Decreases	Ability to pack lunch	Caregiver
Household financial spending	Decreases	Poverty	Caregiver
Financial burden	Decreases	Family breakdown	Caregiver
Domestic violence	Increases	Child taken to grandparents' home	Caregiver
Availability of time for children	Increases	Child-caregiver relationships	Caregiver
Child-caregiver relationships	Increases	Availability of time for children	Caregiver
Availability of time for children	Increases	Caregiver homework support	Caregiver
Physical disability	Decreases	Household financial spending	Caregiver
Ability to buy lamps and kerosene	Decreases	Household financial spending	Caregiver
Household financial spending	Increases	Ability to buy lamps and kerosene	Caregiver
Family size	Increases	Number of children in a home	Caregiver
NGO support	Increases	Cash relief	Caregiver
Bureaucracy of handling domestic violence	Increases	Police corruption	Caregiver
Police corruption	Increases	Domestic violence	Caregiver
Bureaucracy of handling domestic violence	Increases	Domestic violence	Caregiver
Number of children in a home	Decreases	Availability of food at home	Caregiver
Household financial spending	Decreases	Caregiver prioritisation to children in upper classes	Caregiver
Age of the caregiver	Increases	Availability of time for children	Caregiver
Age of the caregiver	Increases	Child-caregiver relationships	Caregiver
Age of the caregiver	Increases	Caregiver homework support	Caregiver
Family breakdown	Increases	Child-headed families	Caregiver

Poverty	Increases	Psychological trauma of family members	Caregiver
Financial burden	Increases	Psychological trauma of family members	Caregiver
Self-neglect	Increases	Family breakdown	Caregiver
Child taken to grandparents' home	Decreases	Availability of time for children	Caregiver
Cash relief	Increases	Household financial spending	Caregiver
Cash relief	Decreases	Overwhelmed foster caregivers	Caregiver
Cash relief	Increases	Ghost recipient	Caregiver
Child-caregiver relationships	Increases	Accompanying a child to school	Caregiver
Accompanying a child to school	Increases	Child-caregiver relationships	Caregiver
Family size	Decreases	Cash relief	Caregiver
School performance	Increases	Attitude of caregivers towards School	School Support
Accountability from School	Increases	Attitude of caregivers towards School	School Support
Quality of teaching	Increases	Attitude of caregivers towards School	School Support
Quality of teaching	Increases	School performance	School Support
Termly School fees	Decreases	Attitude of caregivers towards School	School Support
Class size	Decreases	Attitude of caregivers towards School	School Support
Security at School	Increases	Attitude of caregivers towards School	School Support
Regular attendance	Increases	School performance	School Support
Termly School fees	Decreases	Regular attendance	School Support
School performance	Increases	Quality of teaching	School Support
Class size	Decreases	Regular attendance	School Support
Attitude of caregivers towards School	Increases	Termly School fees	School Support
Caregivers' level of education	Increases	Follow-up of child's progress	Knowledge and Attitude
Caregivers' level of education	Increases	Caregivers' motivation to support child	Knowledge and Attitude
Caregivers' level of education	Increases	Caregivers' attitude towards education	Knowledge and Attitude
Caregivers' motivation to support child	Increases	Caregivers' attitude towards education	Knowledge and Attitude
Caregivers' attitude towards education	Increases	Caregivers' motivation to support child	Knowledge and Attitude
Caregivers' motivation to support child	Increases	Follow-up of child's progress	Knowledge and Attitude
Caregivers' value for education	Increases	Caregivers' attitude towards education	Knowledge and Attitude
Caregivers' value for education	Increases	Caregivers' motivation to support child	Knowledge and Attitude
Caregivers' value for education	Increases	Follow-up of child's progress	Knowledge and Attitude
Caregivers' level of education	Increases	Caregivers' value for education	Knowledge and Attitude

Caregivers' attitude towards education	Increases	Follow-up of child's progress	Knowledge and Attitude

Settlement 3

Variable 1	Polarity	Variable 2	Theme
Household financial spending	Increases	Provision of scholastic material	Caregiver
Provision of scholastic material	Decreases	Household financial spending	Caregiver
Household financial spending	Increases	Availability of food in the home	Caregiver
Household financial spending	Increases	Payment of school fees	Caregiver
Payment of school fees	Decreases	Household financial spending	Caregiver
Poverty	Decreases	Availability of food in the home	Caregiver
Availability of jobs	Increases	Household financial spending	Caregiver
Sale of agricultural produce goats and chicken	Increases	Household financial spending	Caregiver
Household financial spending	Increases	Caregiver motivation to support a child	Caregiver
Availability of food in the home	Increases	Caregiver motivation to support a child	Caregiver
Child-caregiver relationship	Increases	Caregiver motivation to support a child	Caregiver
Child-caregiver relationship	Increases	Caregiver supporting child on homework	Caregiver
Caregiver motivation to support a child	Increases	Caregiver supporting child on homework	Caregiver
Caregiver motivation to support a child	Increases	Child-caregiver relationship	Caregiver
Relationship between husband and wife	Increases	Caregiver supporting child on homework	Caregiver
Relationship between husband and wife	Increases	Child-caregiver relationship	Caregiver
Relationship between husband and wife	Increases	Caregiver motivation to support a child	Caregiver
Happiness of caregivers	Increases	Relationship between husband and wife	Caregiver
Happiness of caregivers	Increases	Caregiver supporting child on homework	Caregiver
Happiness of caregivers	Increases	Caregiver motivation to support a child	Caregiver
Relationship between husband and wife	Increases	Happiness of caregivers	Caregiver
Relationship between husband and wife	Increases	Caregiver’s will to accompany a child to school	Caregiver
Child-caregiver relationship	Increases	Caregiver’s will to accompany a child to school	Caregiver
Illiteracy of caregivers	Decreases	Caregiver supporting child on homework	Caregiver
Number of children	Decreases	Payment of school fees	Caregiver
Number of children	Decreases	Household financial spending	Caregiver
Possibility of repatriation	Decreases	Caregiver motivation to support a child	Caregiver
Alcoholism of Caregiver	Increases	Poverty	Caregiver
Alcoholism of Caregiver	Decreases	Household financial spending	Caregiver
Negative peer influence between caregivers	Increases	Alcoholism of Caregiver	Caregiver
Alcoholism of Caregiver	Increases	Family instability	Caregiver
Poverty	Increases	Family instability	Caregiver
Alcoholism of Caregiver	Decreases	Availability of food in the home	Caregiver
Caregiver pressure to adhere to cultural norms	Decreases	Caregiver motivation to support a child	Caregiver

Support from husband	Increases	Relationship between husband and wife	Caregiver
Support from husband	Increases	Happiness of caregivers	Caregiver
Relationship between husband and wife	Increases	Support from husband	Caregiver
Traffic on the road	Decreases	Caregiver’s will to accompany a child to school	Caregiver
Caregiver’s will to accompany a child to school	Increases	Child-caregiver relationship	Caregiver
Sale of agricultural produce goats and chicken	Increases	Caregiver motivation to support a child	Caregiver
Child respect for caregivers	Increases	Child-caregiver relationship	Caregiver
Child respect for caregivers	Increases	Caregiver motivation to support a child	Caregiver
COVID-19 pandemic lockdown	Increases	Caregiver availability of time with children	Caregiver
Caregiver availability of time with children	Increases	Child-caregiver relationship	Caregiver
Child-caregiver relationship	Increases	Caregiver availability of time with children	Caregiver
Caregiver availability of time with children	Increases	Caregiver supporting child on homework	Caregiver
Caregiver attitude towards the child	Increases	Child-caregiver relationship	Caregiver
Caregiver attitude towards the child	Increases	Caregiver’s will to accompany a child to school	Caregiver
Caregiver attitude towards the child	Increases	Caregiver supporting child on homework	Caregiver
Poverty	Increases	Negative peer influence between caregivers	Caregiver
Poverty	Decreases	Household financial spending	Caregiver
Alcoholism of Caregiver	Decreases	Support from husband	Caregiver
Early pregnancy	Increases	Early marriage	Children
Early pregnancy	Increases	School dropout	Children
School dropout	Increases	Early pregnancy	Children
Early marriage	Increases	School dropout	Children
School dropout	Increases	Early marriage	Children
Child motivation to learn	Decreases	School dropout	Children
Early pregnancy	Decreases	Child motivation to learn	Children
Child's health and wellbeing	Increases	Child motivation to learn	Children
Loss of parents	Decreases	Child motivation to learn	Children
Engaging in agricultural activities	Decreases	Child motivation to learn	Children
Engaging in agricultural activities	Increases	School dropout	Children
Child academic performance	Increases	Child motivation to learn	Children
Child motivation to learn	Increases	Child academic performance	Children
Child academic performance	Increases	Morale of caregivers	Children
Peer pressure	Decreases	Child academic performance	Children
Peer pressure	Decreases	Morale of caregivers	Children
Peer pressure	Decreases	Child's discipline	Children
Child's discipline	Decreases	Peer pressure	Children
Child's discipline	Increases	Child academic performance	Children
Child academic performance	Increases	Child's discipline	Children

Hunger	Decreases	Child motivation to learn	Children
Hunger	Decreases	Child academic performance	Children
Hunger	Decreases	Concentration in class	Children
Engagement in house chores	Decreases	Child motivation to learn	Children
Engagement in house chores	Decreases	Child academic performance	Children
Child Attending School	Increases	Child academic performance	Children
Child Attending School	Increases	Child motivation to learn	Children
Loss of parents	Decreases	Child academic performance	Children
Engaging in agricultural activities	Decreases	Child Attending School	Children
Child academic performance	Decreases	Repeating class	Children
Repeating class	Decreases	Child motivation to learn	Children
Engagement in house chores	Decreases	Child Attending School	Children
Access to sanitary pads	Decreases	Child Attending School	Children
Access to sanitary pads	Increases	Child motivation to learn	Children
Early marriage	Increases	Early pregnancy	Children
Distance to school	Decreases	Child motivation to learn	Children
Distance to school	Decreases	Child Attending School	Children
Distance to school	Increases	Fear of kidnap and rape	Children
Fear of kidnap and rape	Decreases	Child Attending School	Children
Fear of kidnap and rape	Decreases	Child motivation to learn	Children
Child's disability	Decreases	Child academic performance	Children
Child's disability	Decreases	Child Attending School	Children
Corporal punishments	Decreases	Child motivation to learn	Children
Corporal punishments	Decreases	Child Attending School	Children
Bad weather	Decreases	Child Attending School	Children
Child's health and wellbeing	Increases	Concentration in class	Children
Concentration in class	Increases	Child academic performance	Children
Behaviour of siblings	Increases	Morale of caregivers	Children
COVID-19 Lockdown	Increases	Early pregnancy	Children
COVID-19 Lockdown	Decreases	Child motivation to learn	Children
COVID-19 Lockdown	Increases	Early marriage	Children
War	Decreases	Child academic performance	Children
War	Decreases	Child motivation to learn	Children
War	Increases	Hunger	Children
Concentration in class	Increases	Morale of caregivers	Children
War	Increases	Repeating class	Children
Repeating class	Decreases	Morale of caregivers	Children
Child having good friends	Increases	Morale of caregivers	Children
Child having good friends	Increases	Child's discipline	Children
Child having good friends	Decreases	Peer pressure	Children
School dropout	Increases	Engaging in agricultural activities	Children
Child's health and wellbeing	Decreases	School dropout	Children
Morale of caregivers	Increases	Child motivation to learn	Children

Language barrier	Decreases	Teacher-Caregiver relationship	Communication
Language barrier	Decreases	Caregivers follow up with the teacher about child performance.	Communication
Language barrier	Decreases	Attendance of class day meetings	Communication
Attendance of class day meetings	Increases	Caregivers follow up with the teacher about child performance.	Communication
Attendance of class day meetings	Increases	Teacher-Caregiver relationship	Communication
Teacher-Caregiver relationship	Increases	Attendance of class day meetings	Communication
Teacher feedback to caregivers about child performance	Increases	Teacher-Caregiver relationship	Communication
Teacher feedback to caregivers about child performance	Increases	Attendance of class day meetings	Communication
Attendance of class day meetings	Increases	Teacher feedback to caregivers about child performance	Communication
Teacher-Caregiver relationship	Increases	Teacher feedback to caregivers about child performance	Communication
Caregivers follow up with the teacher about child performance.	Increases	Teacher-Caregiver relationship	Communication
Teacher-Caregiver relationship	Increases	Caregivers follow up with the teacher about child performance.	Communication
Teachers and caregivers give consistent support to the child	Increases	Teacher-Caregiver relationship	Communication
Teacher-Caregiver relationship	Increases	Teachers and caregivers give consistent support to the child	Communication
Parents speaking the same language	Decreases	Language barrier	Communication
Parents speaking the same language	Decreases	Attendance of class day meetings	Communication
Language barrier	Decreases	Teacher feedback to caregivers about child performance	Communication
Caregivers' value of education	Increases	Caregivers' attitude towards education	Knowledge and Attitudes
Caregivers' value of education	Increases	Caregivers follow up with the teacher about child performance	Knowledge and Attitudes
Caregivers follow up with the teacher about child performance	Increases	Caregivers' attitude towards education	Knowledge and Attitudes
Caregivers' attitude towards education	Increases	Caregivers follow up with the teacher about child performance	Knowledge and Attitudes
Inspiration of role models e.g. church leaders	Increases	Caregivers' attitude towards education	Knowledge and Attitudes
Inspiration of role models e.g. church leaders	Increases	Caregivers follow up with the teacher about child performance	Knowledge and Attitudes
Learning environment	Increases	Motivation to teach	School Support
Motivation to teach	Increases	Learning environment	School Support
Competent teachers	Increases	Learning environment	School Support
Learning environment	Increases	Competent teachers	School Support
Teacher attitude towards learners	Increases	Learning environment	School Support
Learning environment	Increases	Teacher attitude towards learners	School Support

Settlement 4

Variable 1	Polarity	Variable 2	Category
Language barrier	Decreases	Caregiver-teacher relationships	Communication
Language barrier	Decreases	Teacher feedback to caregivers	Communication
Language barrier	Decreases	Caregiver - teacher communication	Communication
Caregiver - teacher communication	Increases	Teacher feedback to caregivers	Communication
Invitation of caregivers to School	Increases	Caregiver - teacher communication	Communication
Caregiver desire to engage in school activities	Increases	Caregiver visiting school on their own initiative	Communication
Caregiver-teacher relationships	Increases	Caregiver visiting school on their own initiative	Communication
Teacher feedback to caregivers	Increases	Caregiver-teacher relationships	Communication
Language barrier	Decreases	Caregiver desire to engage in school activities	Communication
Language barrier	Decreases	Caregiver visiting school on their own initiative	Communication
Caregiver - teacher communication	Increases	Regular meetings between caregiver and teachers	Communication
Regular meetings between caregiver and teachers	Increases	Caregiver-teacher relationships	Communication
Caregiver visiting school on their own initiative	Increases	Regular meetings between caregiver and teachers	Communication
Regular meetings between caregiver and teachers	Increases	Caregiver desire to engage in school activities	Communication
Caregiver desire to engage in school activities	Increases	Invitation of caregivers to School	Communication
Language barrier	Decreases	Invitation of caregivers to School	Communication
Corporal punishment	Decreases	Child attending school	Children
Hunger	Decreases	Concentration in class	Children
Child motivation to learn	Increases	Child academic performance	Children
Child academic performance	Increases	Child motivation to learn	Children
Child health and wellbeing	Increases	Child's hope to further studies	Children
Child attending school	Increases	Ability to learn	Children
Ability to learn	Increases	Child academic performance	Children
Support to teenage mothers	Decreases	Early pregnancy	Children
Early pregnancy	Increases	Dropout of School	Children
Early pregnancy	Increases	Early marriage	Children
Early pregnancy	Decreases	Child motivation to learn	Children
Early marriage	Decreases	Child motivation to learn	Children
Child engagement in domestic work	Decreases	Child attending school	Children
Child's inferiority complex	Decreases	Child motivation to learn	Children
Child's inferiority complex	Increases	Dropout of School	Children
Early marriage	Increases	Dropout of School	Children
Child headed family	Decreases	Child's hope to further studies	Children
Child headed family	Decreases	Child motivation to learn	Children
Peer pressure and bullying	Increases	Drug abuse	Children

Concentration in class	Increases	Child academic performance	Children
Child academic performance	Increases	Child's hope to further studies	Children
Child motivation to learn	Increases	Child's hope to further studies	Children
Child discipline	Increases	Child academic performance	Children
Child academic performance	Increases	Child discipline	Children
Child motivation to learn	Increases	Child attending school	Children
Concentration in class	Increases	Ability to learn	Children
Child health and wellbeing	Increases	Child motivation to learn	Children
Child discipline	Increases	Child's hope to further studies	Children
Child discipline	Increases	Concentration in class	Children
Child's hope to further studies	Increases	Child academic performance	Children
Distance to School	Increases	Child's fear of rape and kidnapping	Children
Child's fear of rape and kidnapping	Increases	Child's fear of going to school	Children
Distance to School	Increases	Child's fear of going to school	Children
Distance to School	Decreases	Child motivation to learn	Children
Distance to School	Increases	Child's urge to be accompanied	Children
Child's fear of going to school	Increases	Child's urge to be accompanied	Children
Child's fear of rape and kidnapping	Increases	Child's urge to be accompanied	Children
Child health and wellbeing	Decreases	Dropout of School	Children
Hunger	Decreases	Ability to learn	Children
Child's urge to be accompanied	Decreases	Child's fear of rape and kidnapping	Children
Child's urge to be accompanied	Increases	Child motivation to learn	Children
Early pregnancy	Increases	Child's inferiority complex	Children
Corporal punishment	Decreases	Child motivation to learn	Children
Peer pressure and bullying	Decreases	Child attending school	Children
Child attending school	Decreases	Dropout of School	Children
Drug abuse	Decreases	Child attending school	Children
Children enrol in AEP	Decreases	Child academic performance	Children
Child academic performance	Decreases	AEP students return to mainstream	Children
Child motivation to learn	Decreases	Dropout of School	Children
AEP students return to mainstream	Decreases	Child motivation to learn	Children
Child academic performance	Increases	Child usefulness to family and community	Children
Having school and basic needs	Increases	Child attending school	Children
Having school and basic needs	Decreases	Child's inferiority complex	Children
Hunger	Decreases	Child attending school	Children
Orphans unable to support themselves	Decreases	Child attending school	Children
Bad weather.	Decreases	Child attending school	Children
Child usefulness to family and community	Increases	Child attending school	Children
Caregiver motivation to support children's learning	Increases	Caregivers' attitude towards education	Knowledge and Attitudes
Caregivers' attitude towards education	Increases	Caregiver motivation to support children's learning	Knowledge and Attitudes

Caregivers' attitude towards education	Increases	Caregivers follow up with the teacher about child performance	Knowledge and Attitudes
Caregivers follow up with the teacher about child performance	Increases	Caregivers' attitude towards education	Knowledge and Attitudes
Caregiver motivation to support children's learning	Increases	Caregivers follow up with the teacher about child performance	Knowledge and Attitudes
Caregivers follow up with the teacher about child performance	Increases	Caregiver motivation to support children's learning	Knowledge and Attitudes
Caregiver's value of education	Increases	Caregivers' attitude towards education	Knowledge and Attitudes
Caregiver's value of education	Increases	Caregiver motivation to support children's learning	Knowledge and Attitudes
Caregiver's value of education	Increases	Caregivers follow up with the teacher about child performance	Knowledge and Attitudes
Caregiver's level of education	Increases	Caregiver motivation to support children's learning	Knowledge and Attitudes
Caregiver's level of education	Increases	Caregivers' attitude towards education	Knowledge and Attitudes
Caregiver's level of education	Increases	Caregivers follow up with the teacher about child performance	Knowledge and Attitudes
Inspiration from other children as role models	Increases	Caregiver motivation to support children's learning	Knowledge and Attitudes
Inspiration from other children as role models	Increases	Caregivers' attitude towards education	Knowledge and Attitudes
Child motivation to learn.	Increases	Caregivers' attitude towards education	Knowledge and Attitudes
Caregivers' attitude towards education	Increases	Child motivation to learn.	Knowledge and Attitudes
COVID-19 lockdown	Increases	Availability of time with children	Caregivers
Availability of time with children	Increases	Homework support	Caregivers
Brewing alcohol	Increases	Household financial spending	Caregivers
Number of children in a home	Decreases	Availability of food	Caregivers
Food SACCO	Increases	Availability of food	Caregivers
Availability of food	Increases	Household financial spending	Caregivers
Household financial spending	Increases	Provision of scholastic materials	Caregivers
Provision of scholastic materials	Decreases	Household financial spending	Caregivers
Household financial spending	Increases	Provision of school and exam fees	Caregivers
Provision of school and exam fees	Decreases	Household financial spending	Caregivers
Household financial spending	Increases	Provision of basic needs like soap etc.	Caregivers
Provision of basic needs like soap etc.	Decreases	Household financial spending	Caregivers
Child-parent relationships	Increases	Availability of time with children	Caregivers
Homework support	Increases	Child-parent relationships	Caregivers
Domestic violence	Increases	Family breakdown	Caregivers
Family breakdown	Increases	Single parenthood	Caregivers
Single parenthood	Decreases	Availability of time with children	Caregivers
Household financial spending	Decreases	Poverty	Caregivers
Poverty	Increases	Domestic violence	Caregivers
Alcoholism	Increases	Poverty	Caregivers

Responsible caregiver	Increases	Availability of food	Caregivers
Responsible caregiver	Increases	Household financial spending	Caregivers
Responsible caregiver	Increases	Availability of time with children	Caregivers
Disability of caregivers	Decreases	Household financial spending	Caregivers
Alcoholism	Decreases	Household financial spending	Caregivers
Number of children in a home	Decreases	Household financial spending	Caregivers
Responsible caregiver	Increases	Homework support	Caregivers
Alcoholism	Decreases	Availability of time with children	Caregivers
Availability of time with children	Increases	Engagement in Agriculture	Caregivers
Business opportunities for caregivers	Increases	Household financial spending	Caregivers
Household financial spending	Increases	Availability of food	Caregivers
Engagement in Agriculture	Increases	Availability of food	Caregivers
Brewing alcohol	Increases	Alcoholism	Caregivers
Alcoholism	Increases	Domestic violence	Caregivers
Availability of time with children	Increases	Child-parent relationships	Caregivers
Homework support	Increases	Availability of time with children	Caregivers
Caregivers separated across countries	Increases	Single parenthood	Caregivers
Bad weather	Decreases	Business opportunities for caregivers	Caregivers
Class size	Decreases	Quality of facilities at school	School Support
Quality of facilities at school	Increases	Learning environment	School Support
Class size	Decreases	Learning environment	School Support
Quality of facilities at school	Increases	Attitude of caregivers towards the school administration	School Support
Class size	Increases	School population	School Support
Number of teachers	Increases	School population	School Support
Number of teachers	Increases	Performance of teacher on duty	School Support
School population	Decreases	Security at School	School Support
Number of teachers	Increases	Quality of teaching	School Support
Class size	Decreases	Quality of teaching	School Support
School population	Increases	Class size	School Support
Security at School	Increases	Attitude of caregivers towards the school administration	School Support
Quality of teaching	Increases	Attitude of caregivers towards the school administration	School Support
Number of teachers	Increases	Attitude of caregivers towards the school administration	School Support
Performance of teacher on duty	Increases	Attitude of caregivers towards the school administration	School Support
Security at School	Increases	Learning environment	School Support
Learning environment	Increases	Attitude of caregivers towards the school administration	School Support
Class size	Increases	Number of teachers	School Support
