# Supplementary material for: Increasing school attendance and retention in refugee settings: Participatory development of the SchoolLinks intervention
Source: PLoS One. 2026 Aug 31;21(8):e0357215. doi: 10.1371/journal.pone.0357215 (PMC13528952; doi:10.1371/journal.pone.0357215)

S1 Figure. Causal loop diagrams extracted from the connection circles in the group model building workshops.

Contents

Settlement 1 2

Settlement 2 4

Settlement 3 7

Settlement 4 10

Table of figures

Figure 1. Settlement 1 knowledge and attitudes 2
Figure 2. Settlement 1 communication 2
Figure 3. Settlement 1 child context 3
Figure 4. Settlement 1 caregiver context 3
Figure 5. Settlement 1 school context 4
Figure 6. Settlement 2 knowledge and attitudes 4
Figure 7. Settlement 2 communication 5
Figure 8. Settlement 2 child context 5
Figure 9. Settlement 2 caregiver context 6
Figure 10. Settlement 2 school context 6
Figure 11. Settlement 3 knowledge and attitudes 7
Figure 12. Settlement 3 communication 7
Figure 13. Settlement 3 child context 8
Figure 14. Settlement 3 caregiver 9
Figure 15. Settlement 3 knowledge and attitudes 10
Figure 16. Settlement 4 knowledge and attitudes 10
Figure 17. Settlement 4 communication 10
Figure 18. Settlement 4 child context 12
Figure 19. Settlement 4 school context 12
Figure 20. Settlement 4 caregiver context 13

Settlement 1

Figure 1. Settlement 1 knowledge and attitudes

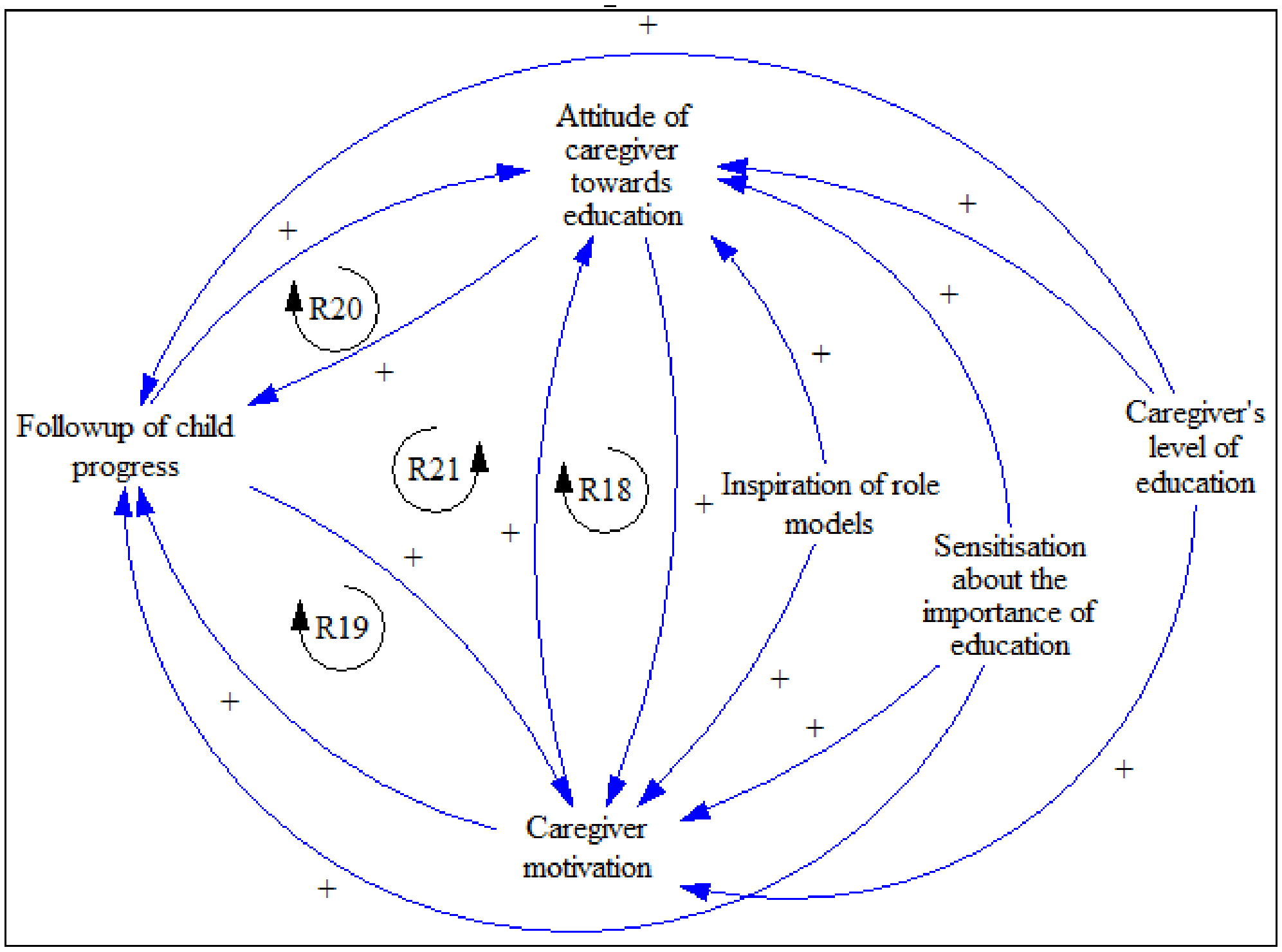

Figure 2. Settlement 1 communication

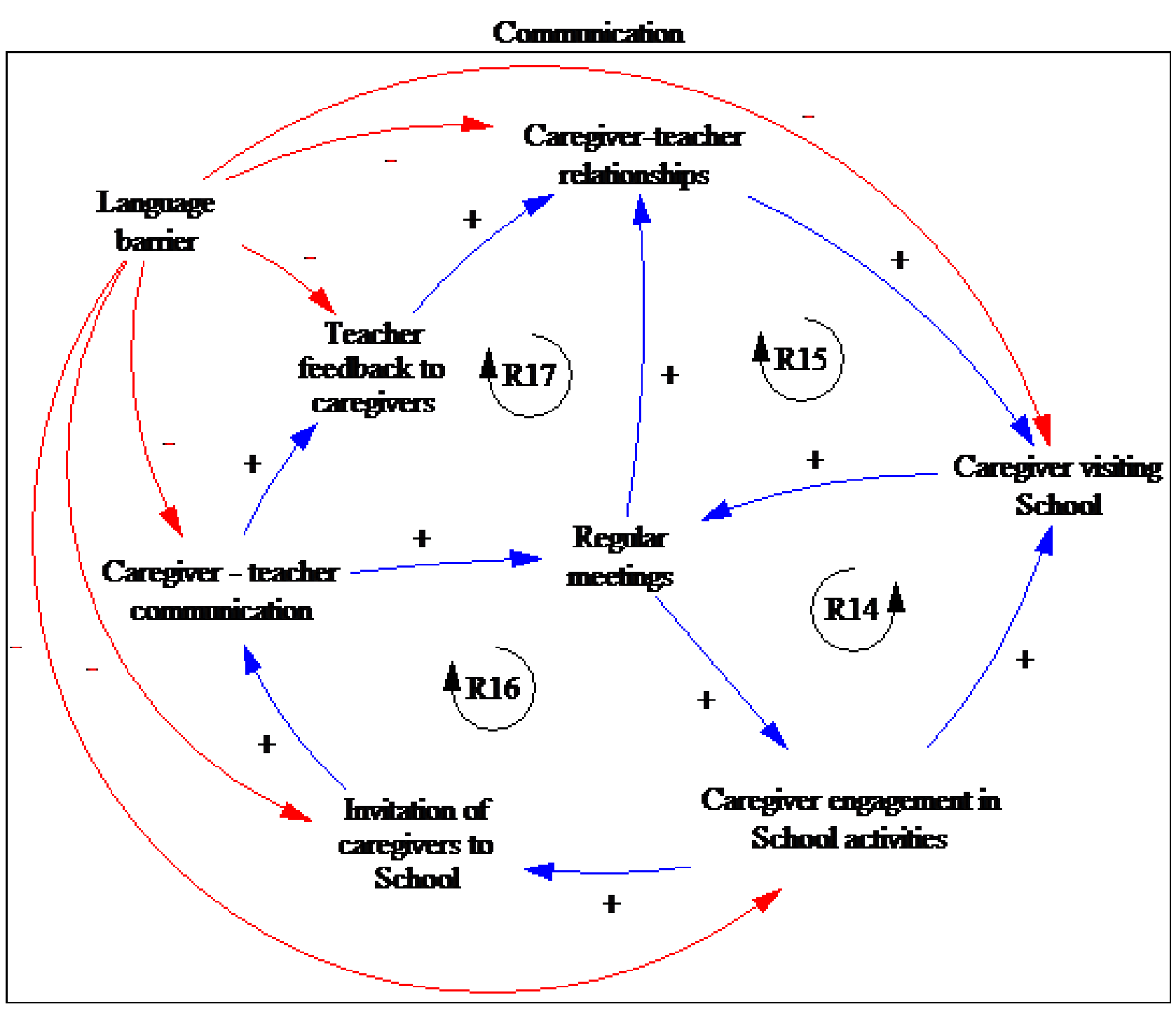

Figure 3. Settlement 1 child context

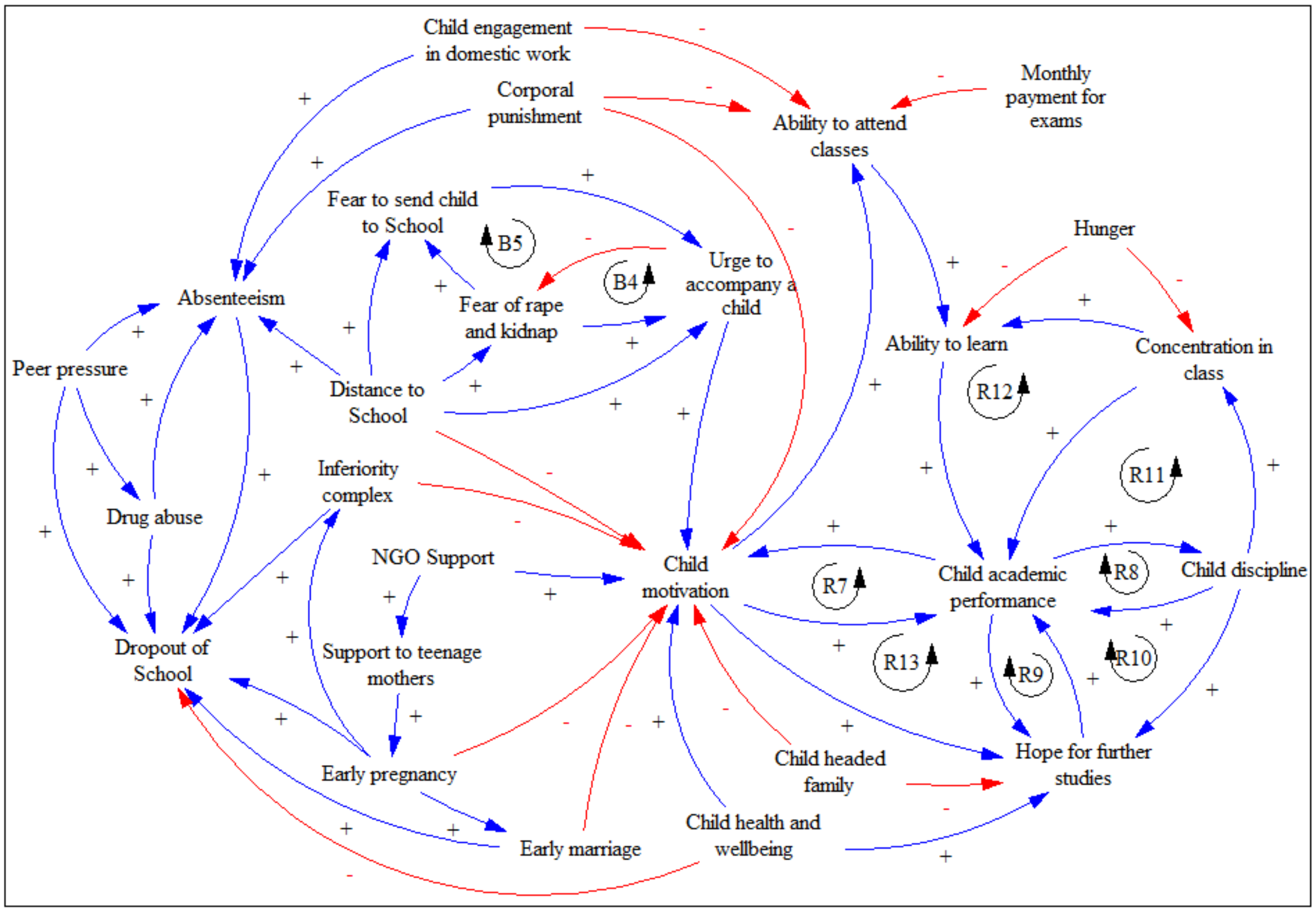

Figure 4. Settlement 1 caregiver context

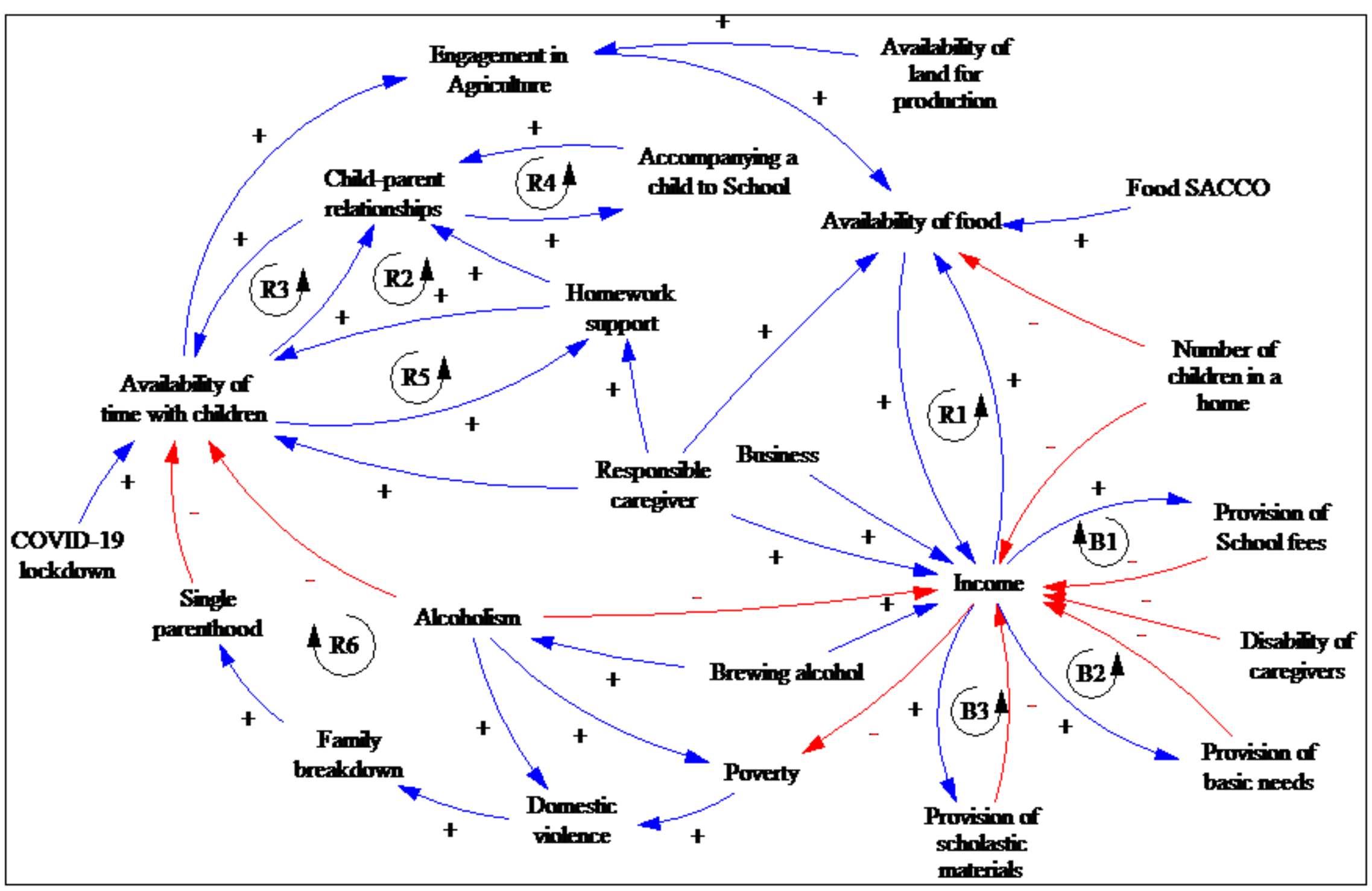

Figure 5. Settlement 1 school context

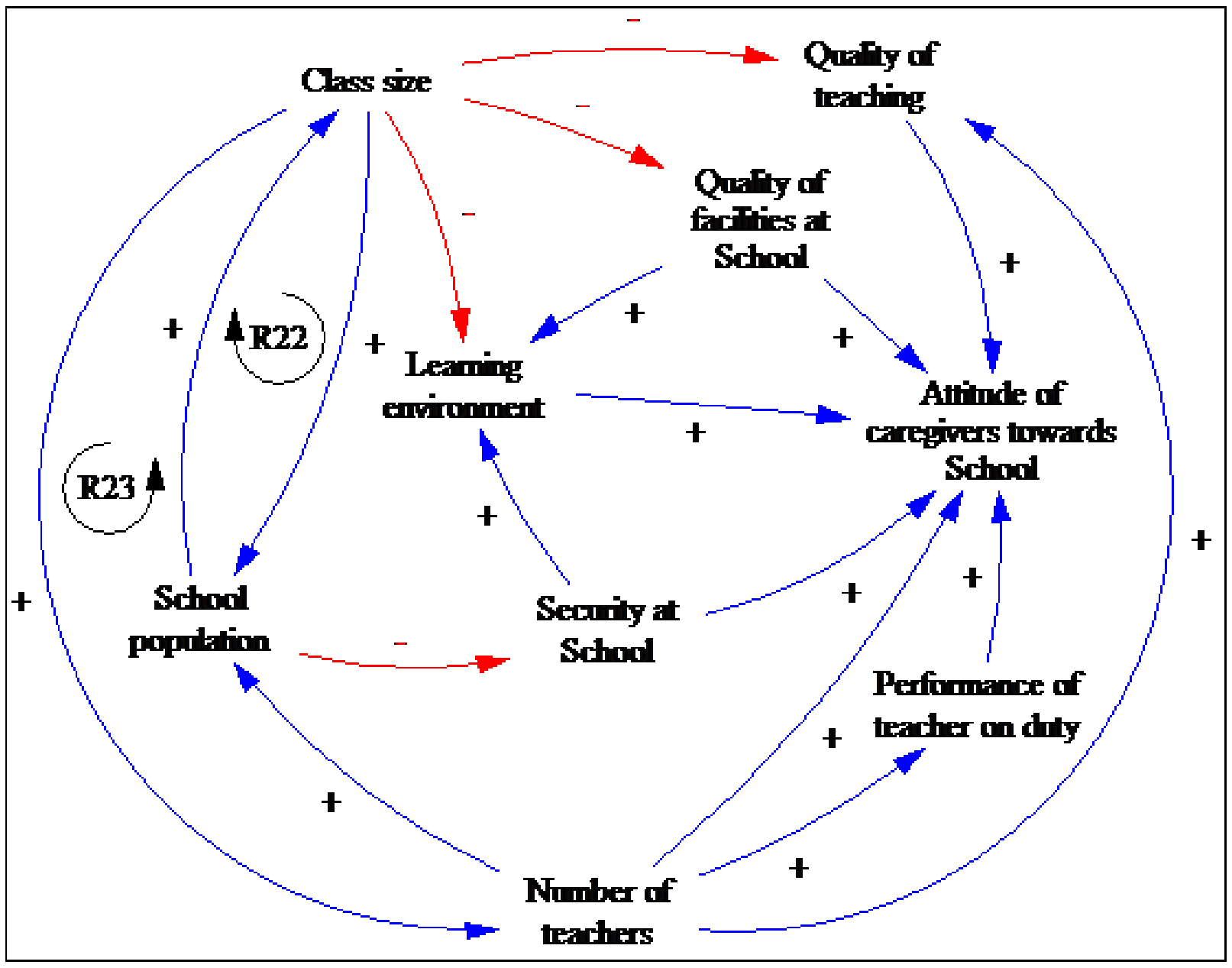

Settlement 2

Figure 6. Settlement 2 knowledge and attitudes

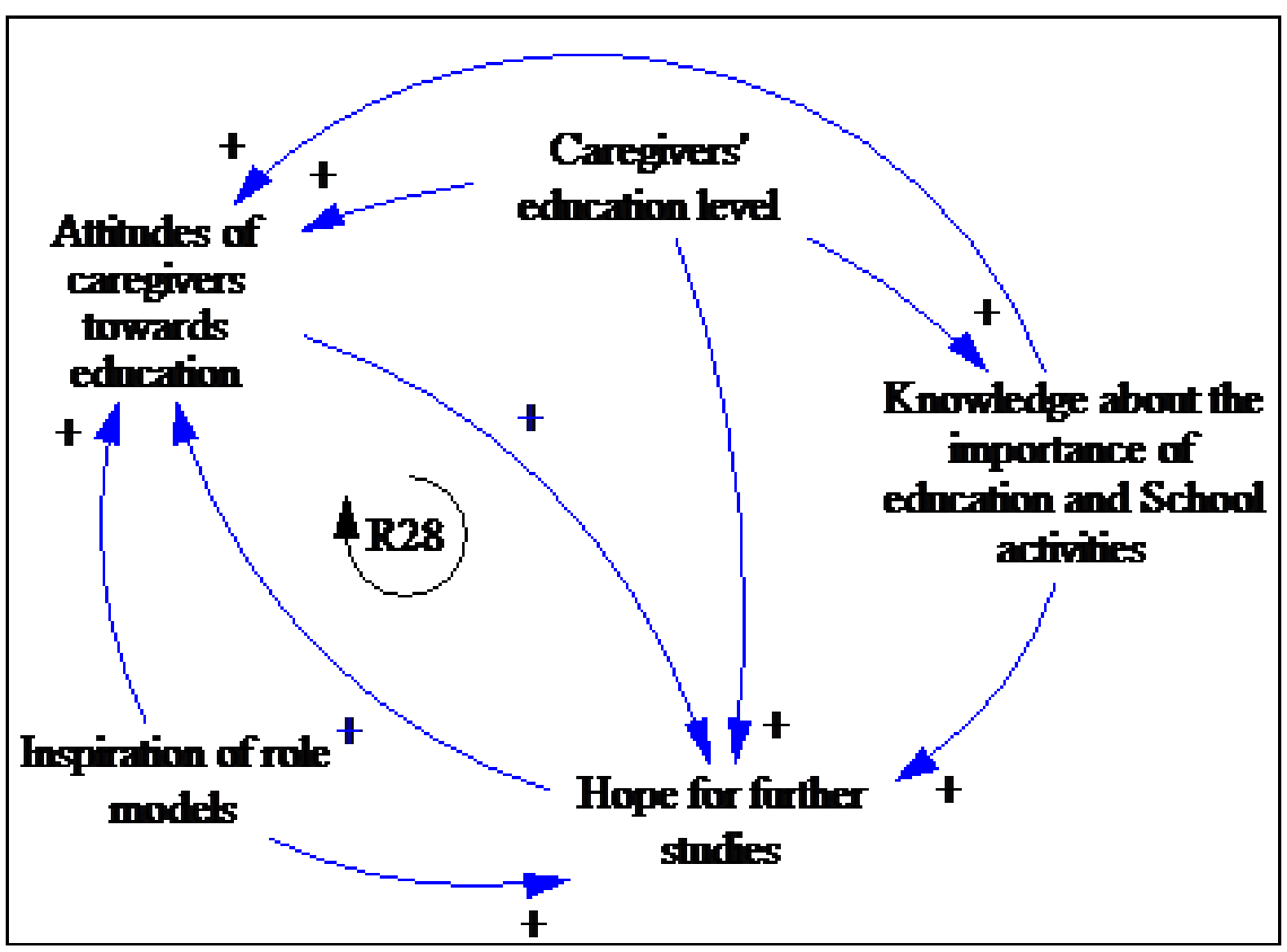

Figure 7. Settlement 2 communication

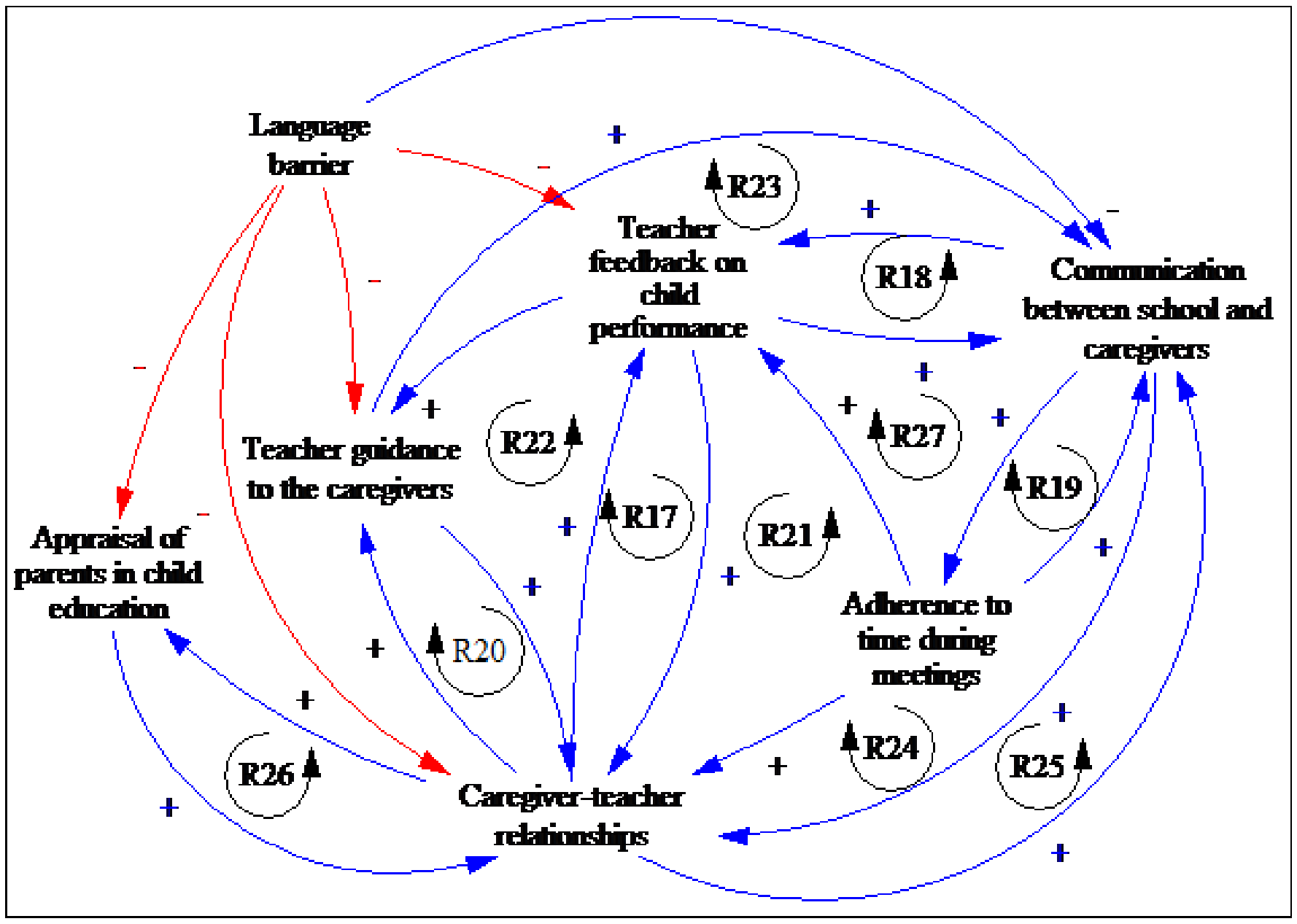

Figure 8. Settlement 2 child context

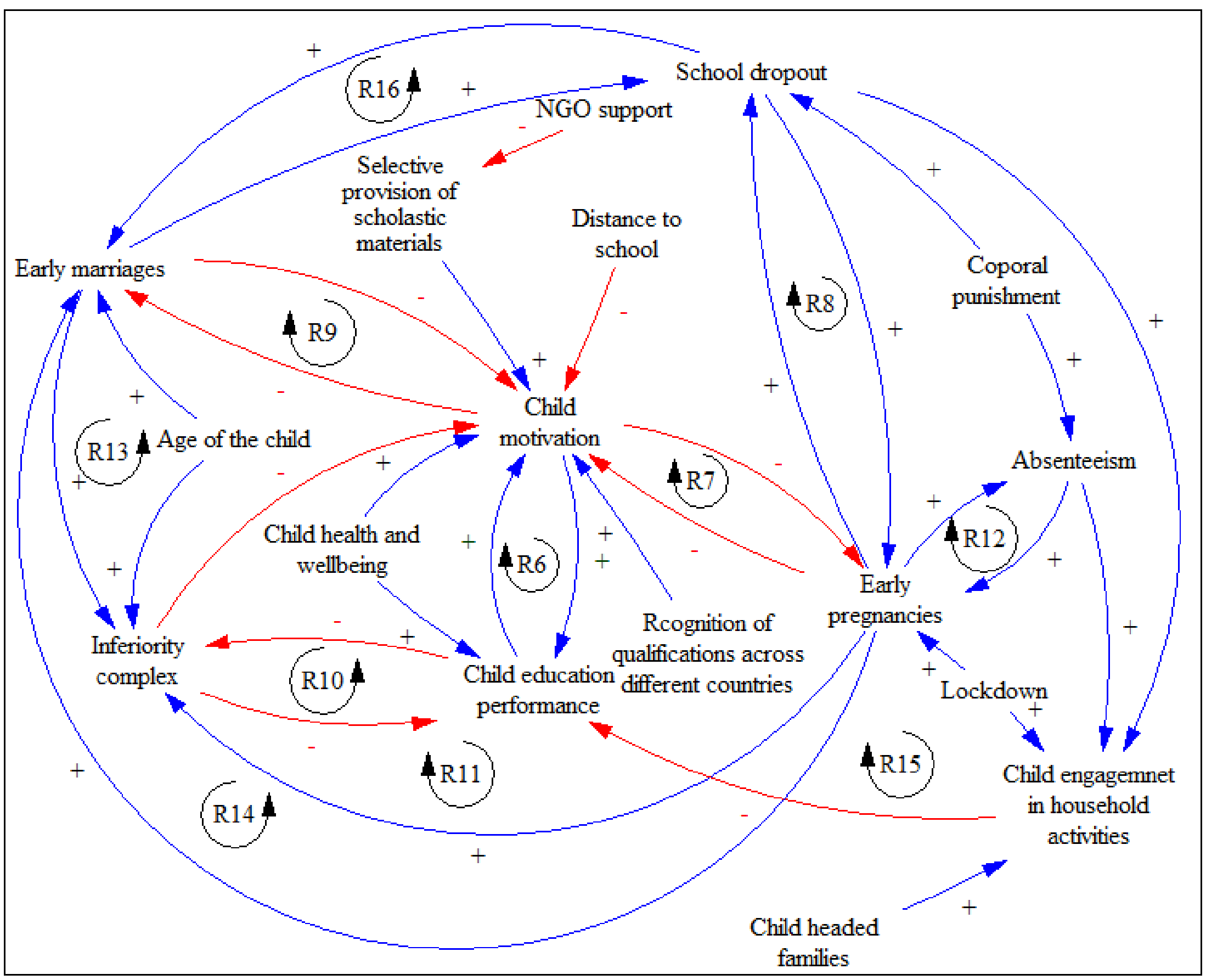

Figure 9. Settlement 2 caregiver context

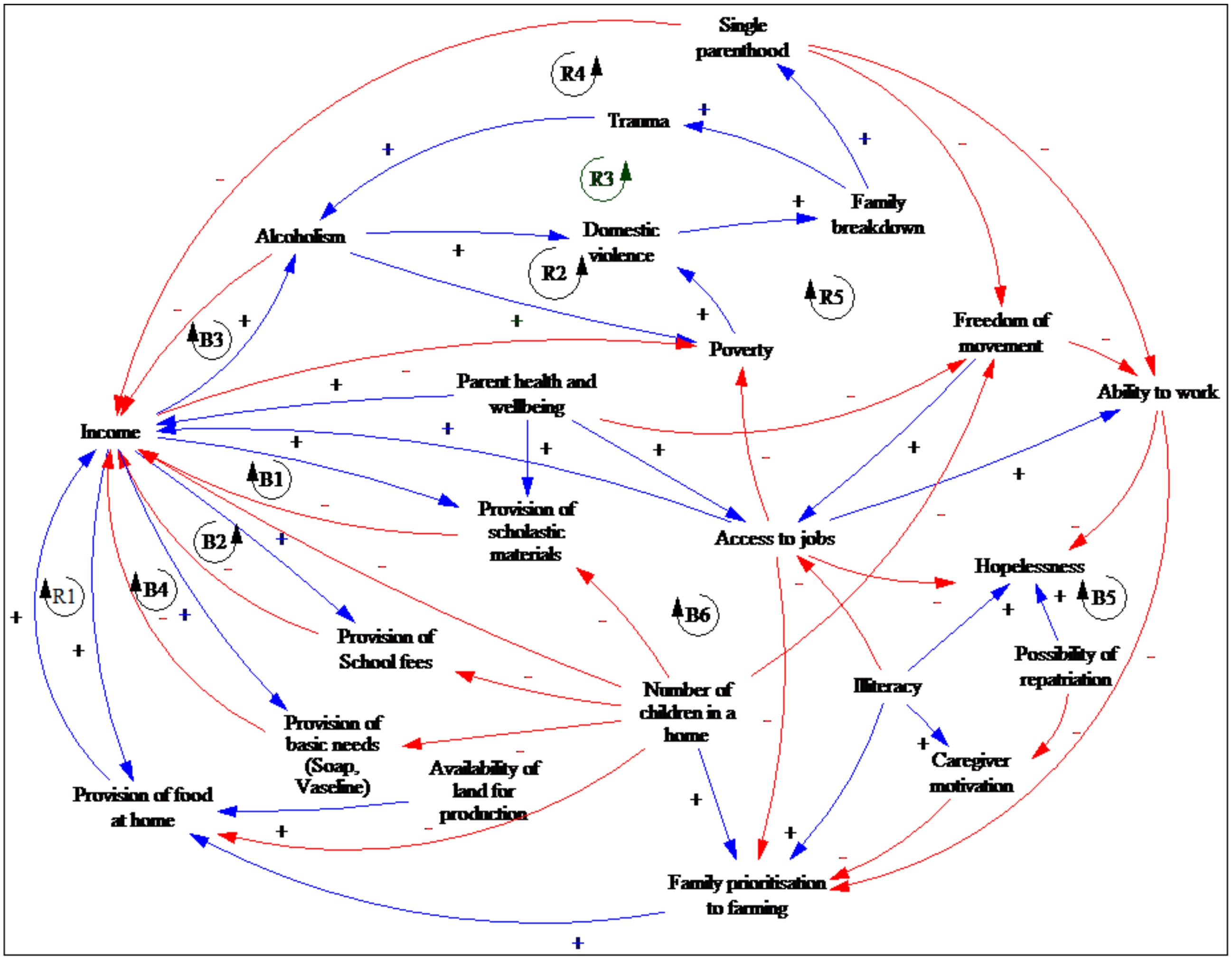

Figure 10. Settlement 2 school context

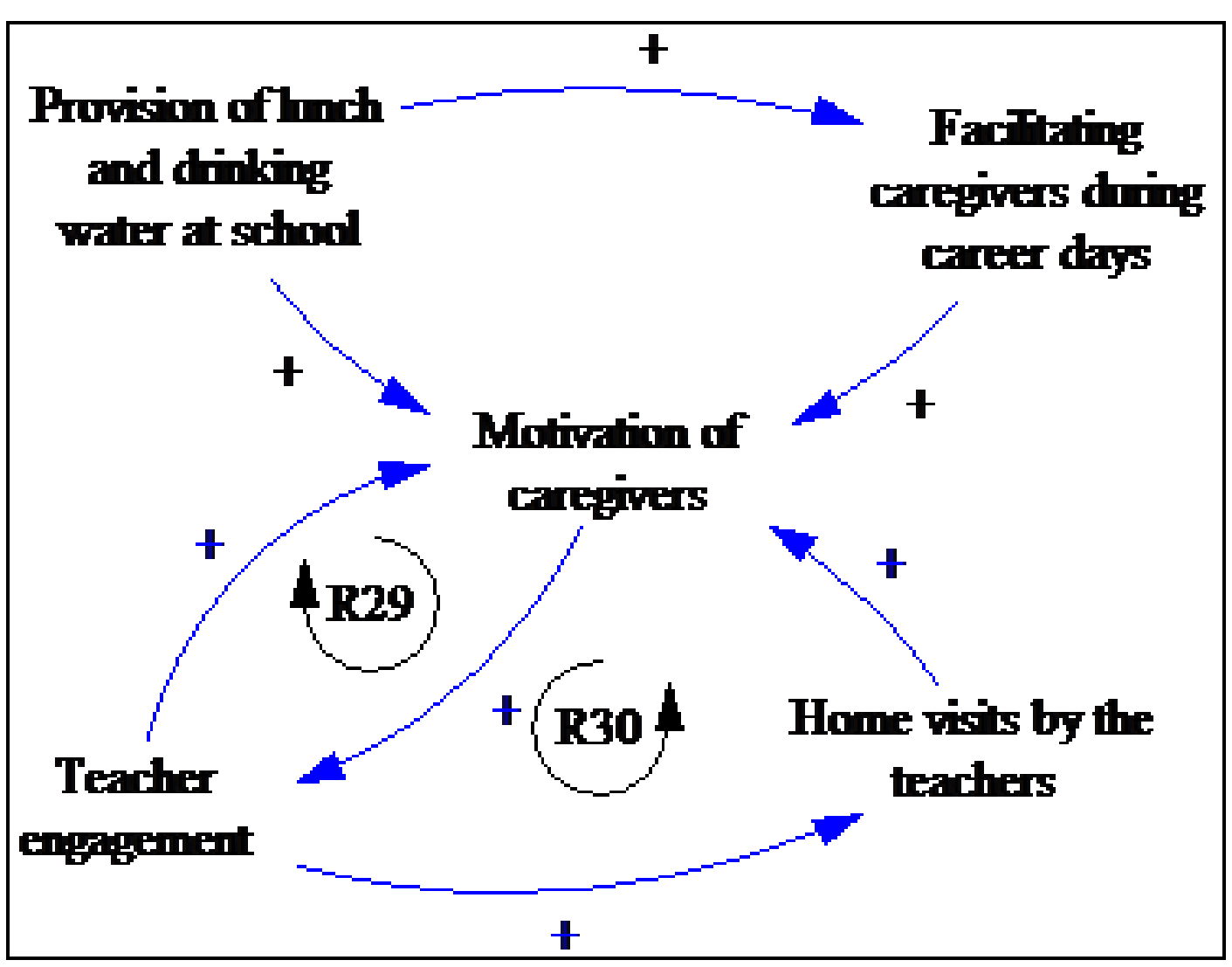

Settlement 3

Figure 11. Settlement 3 knowledge and attitudes

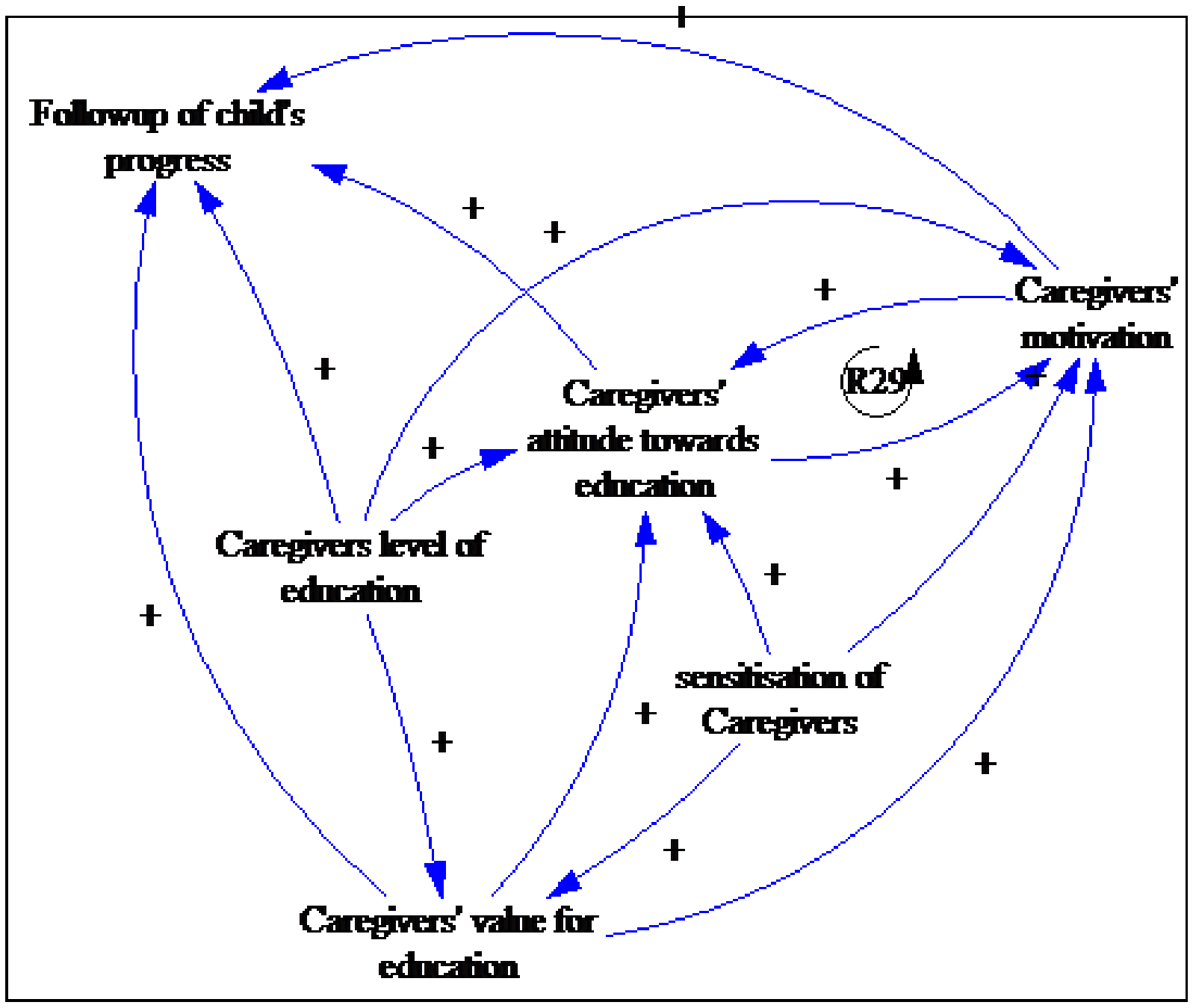

Figure 12. Settlement 3 communication

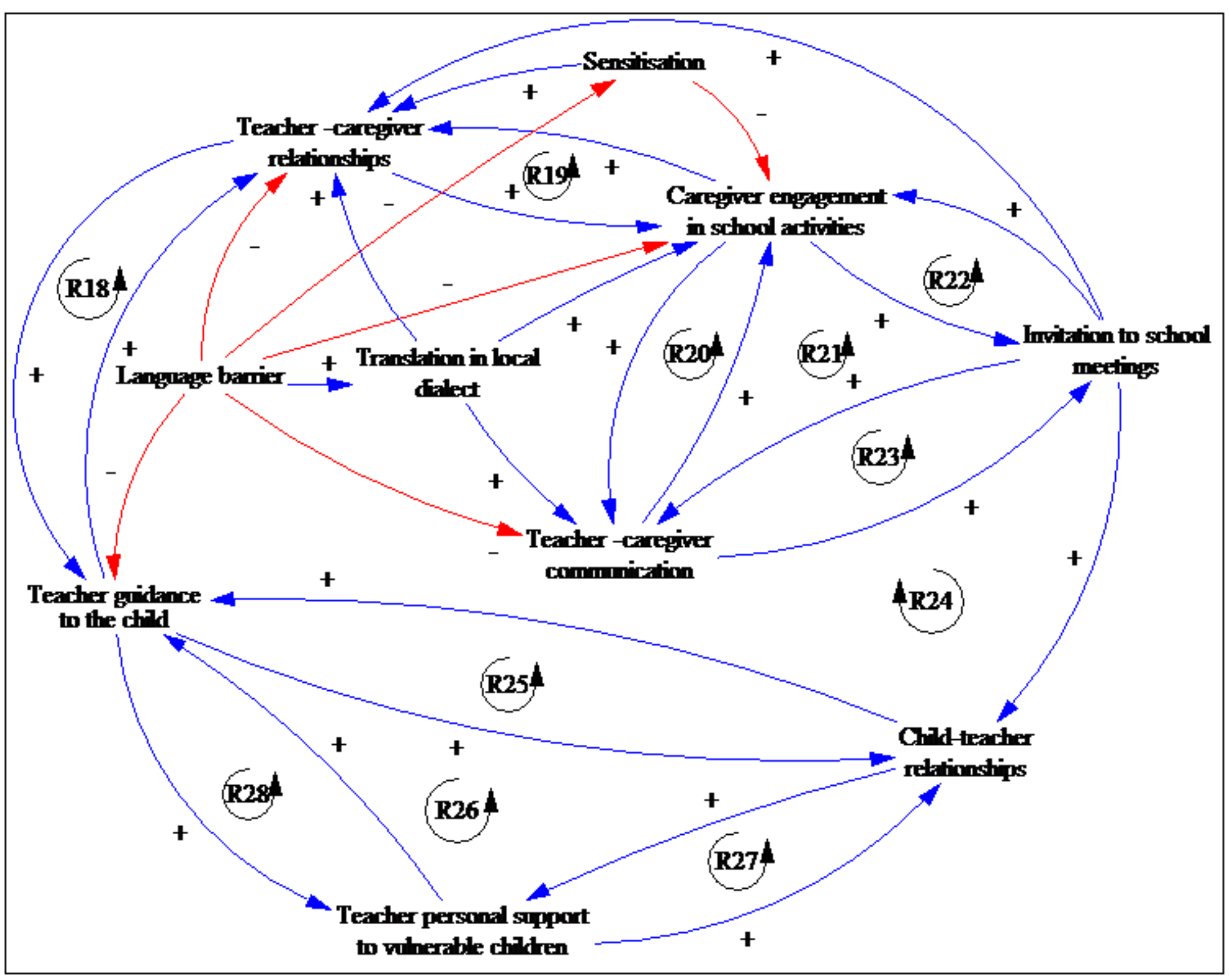

Figure 13. Settlement 3 child context

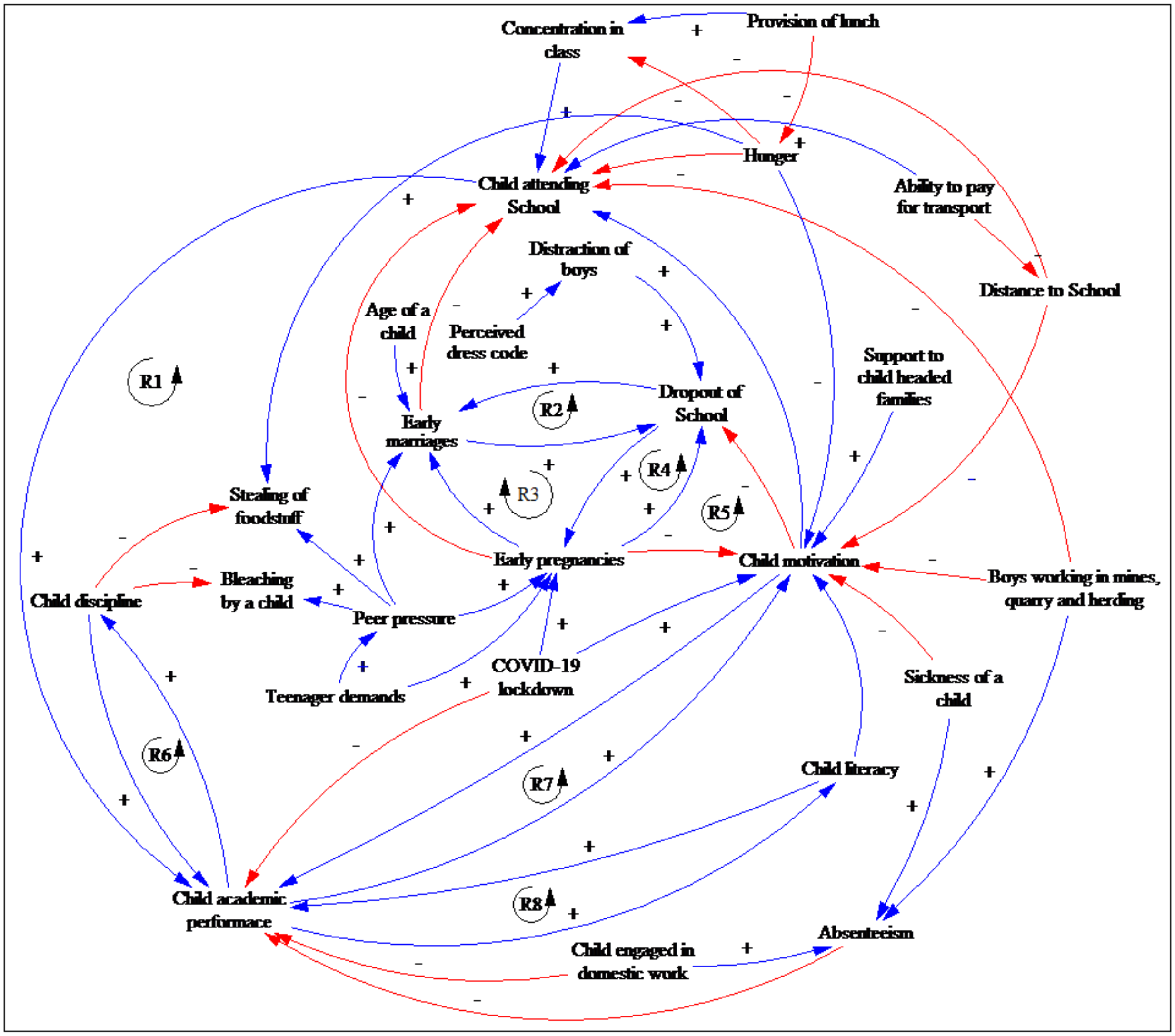

Figure 14. Settlement 3 caregiver

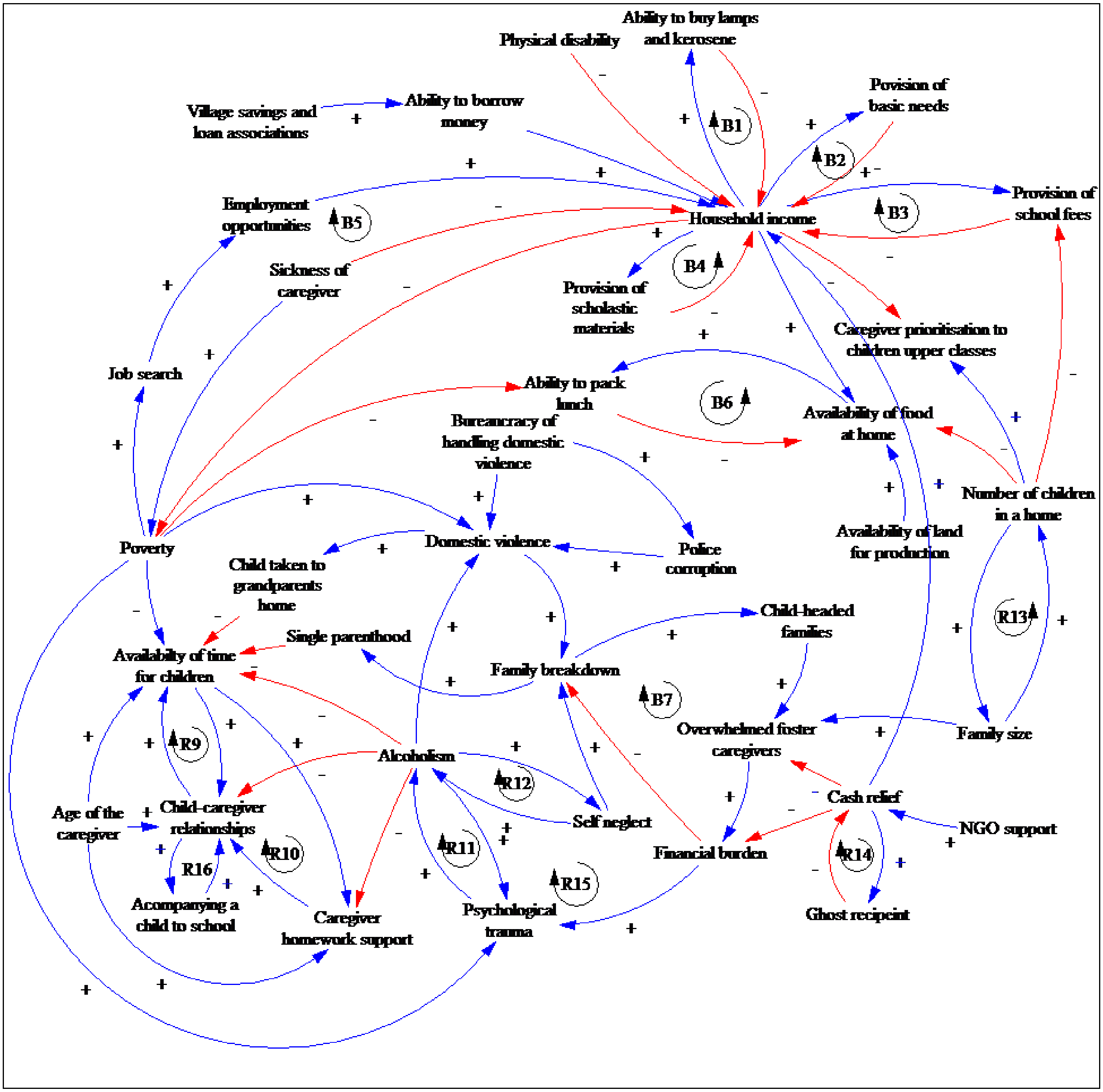

Figure 15. Settlement 3 knowledge and attitudes

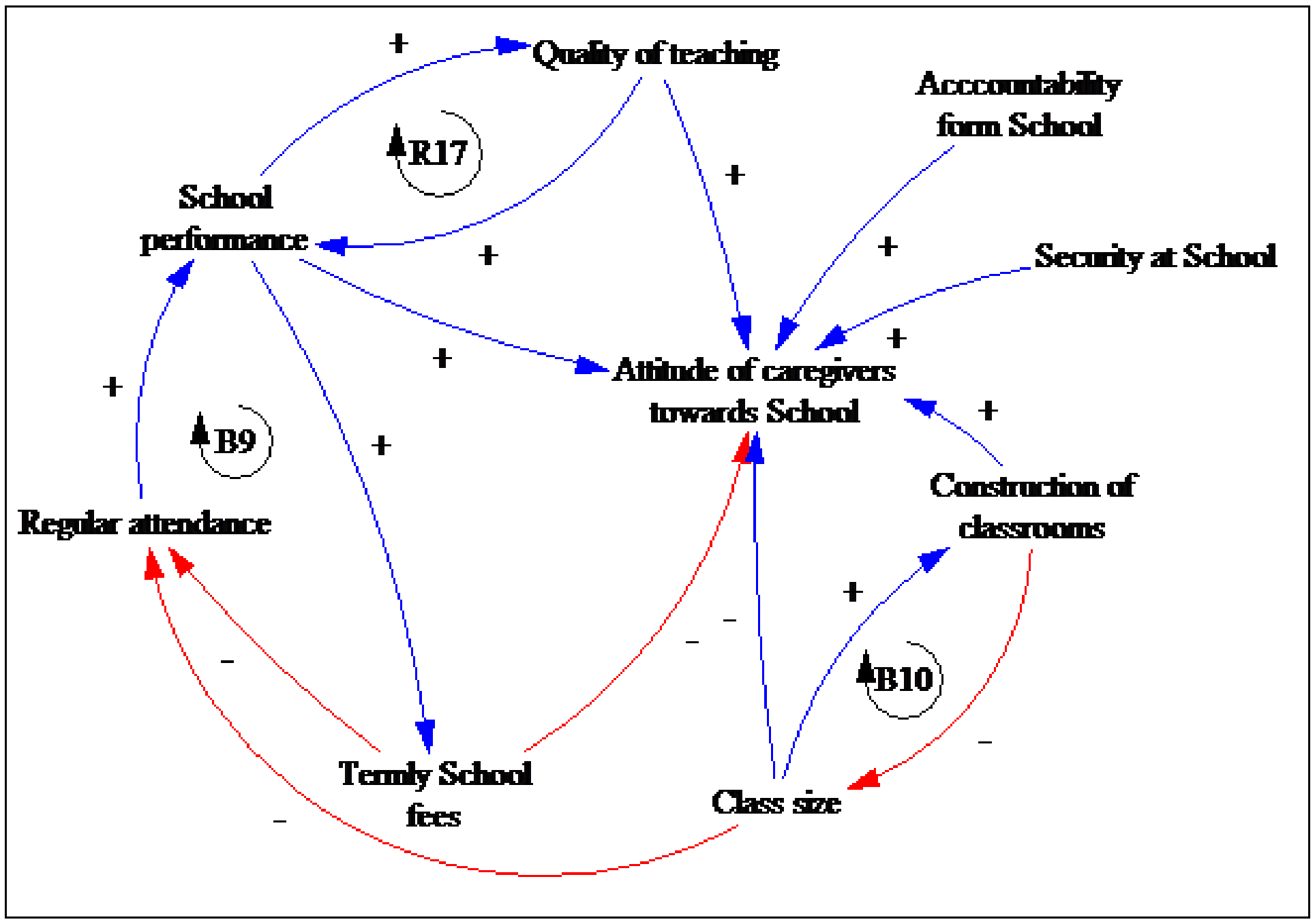

Settlement 4

Figure 16. Settlement 4 knowledge and attitudes

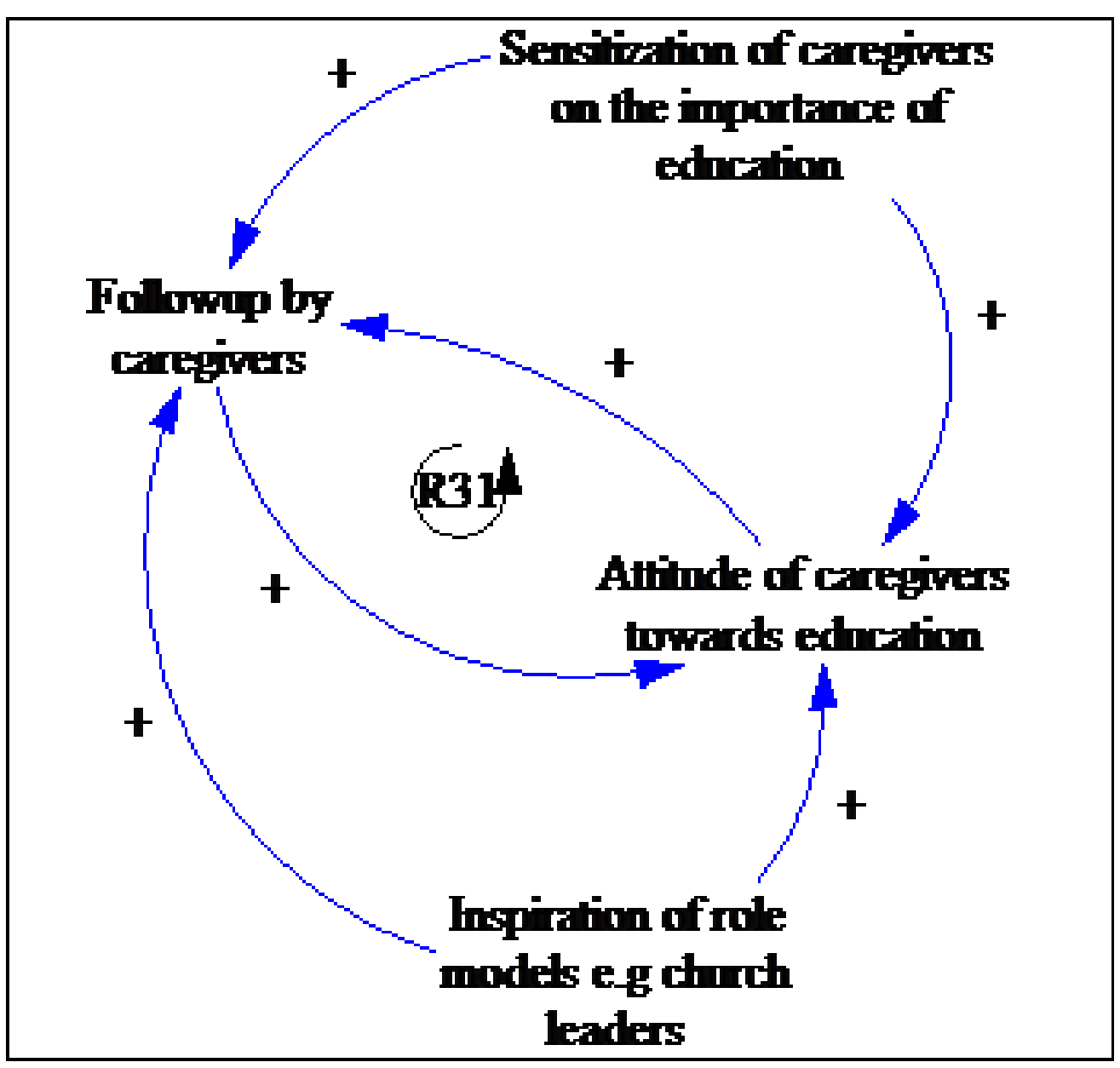

Figure 17. Settlement 4 communication

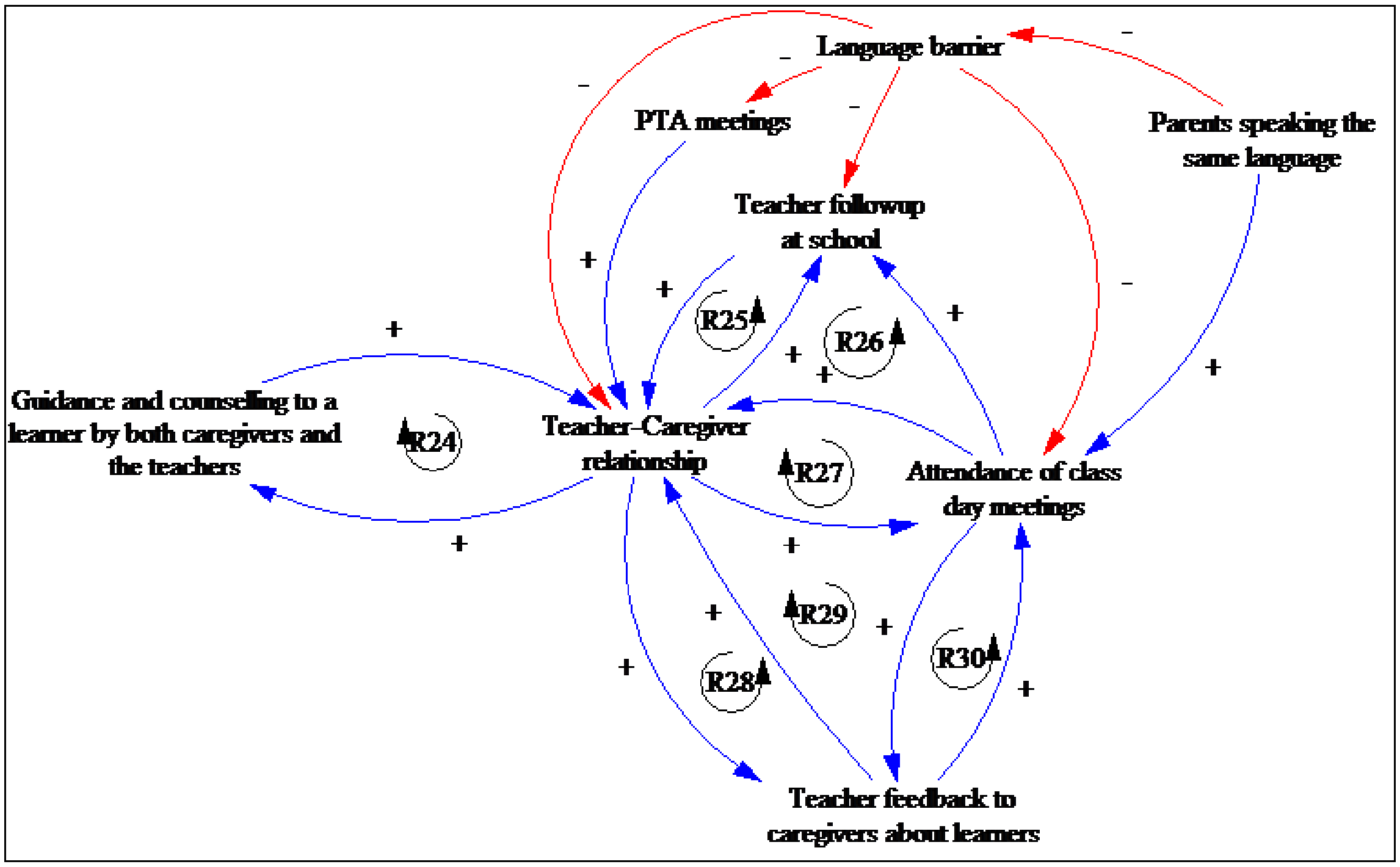

Language barrier
PTA meetings
Parents speaking the same language
Teacher followup at school
R25
R26
Guidance and counselling to a learner by both caregivers and the teachers
R24
Teacher-Caregiver relationship
R27
Attendance of class day meetings
R29
R28
R30
Teacher feedback to caregivers about learners

Figure 18. Settlement 4 child context

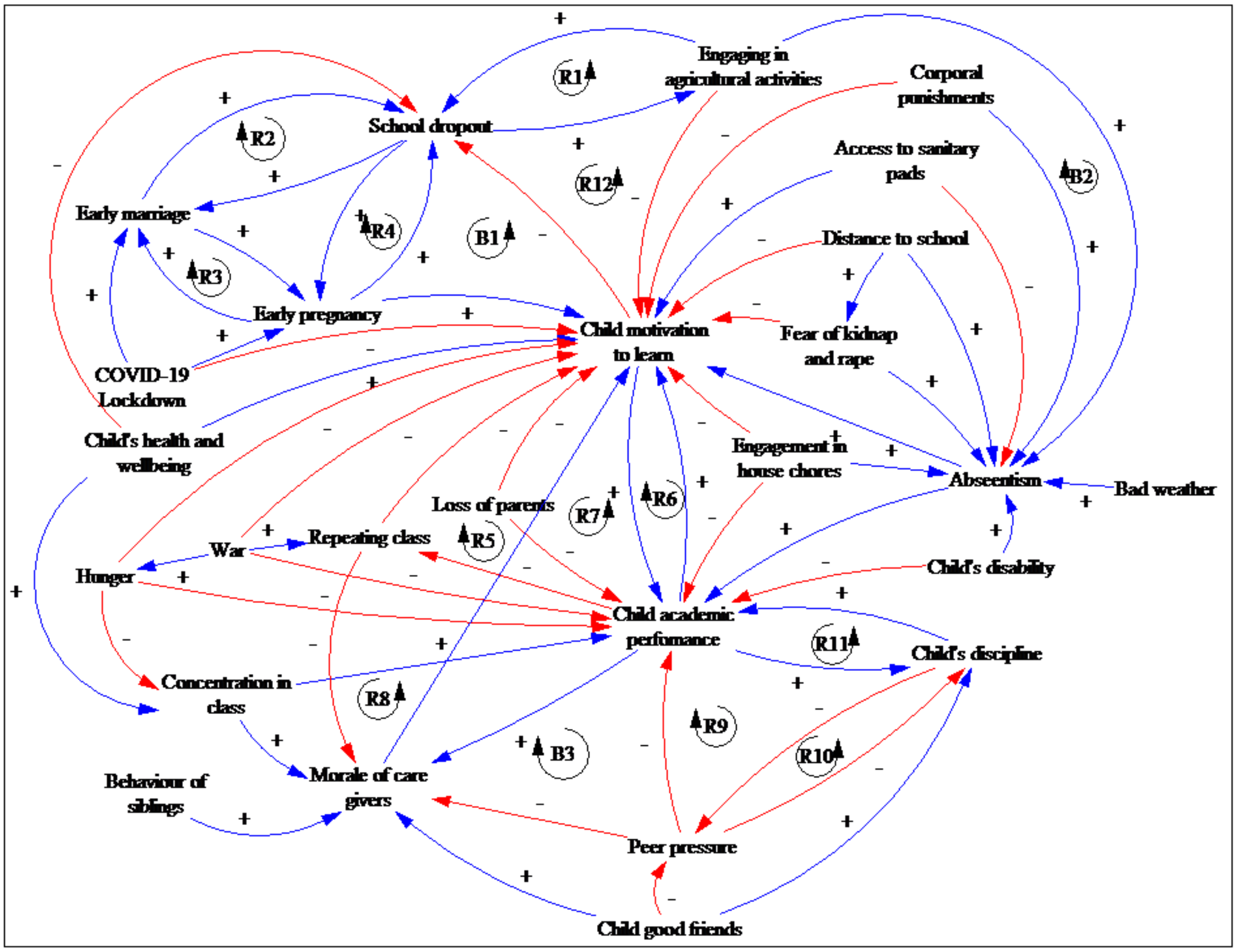

Figure 19. Settlement 4 school context

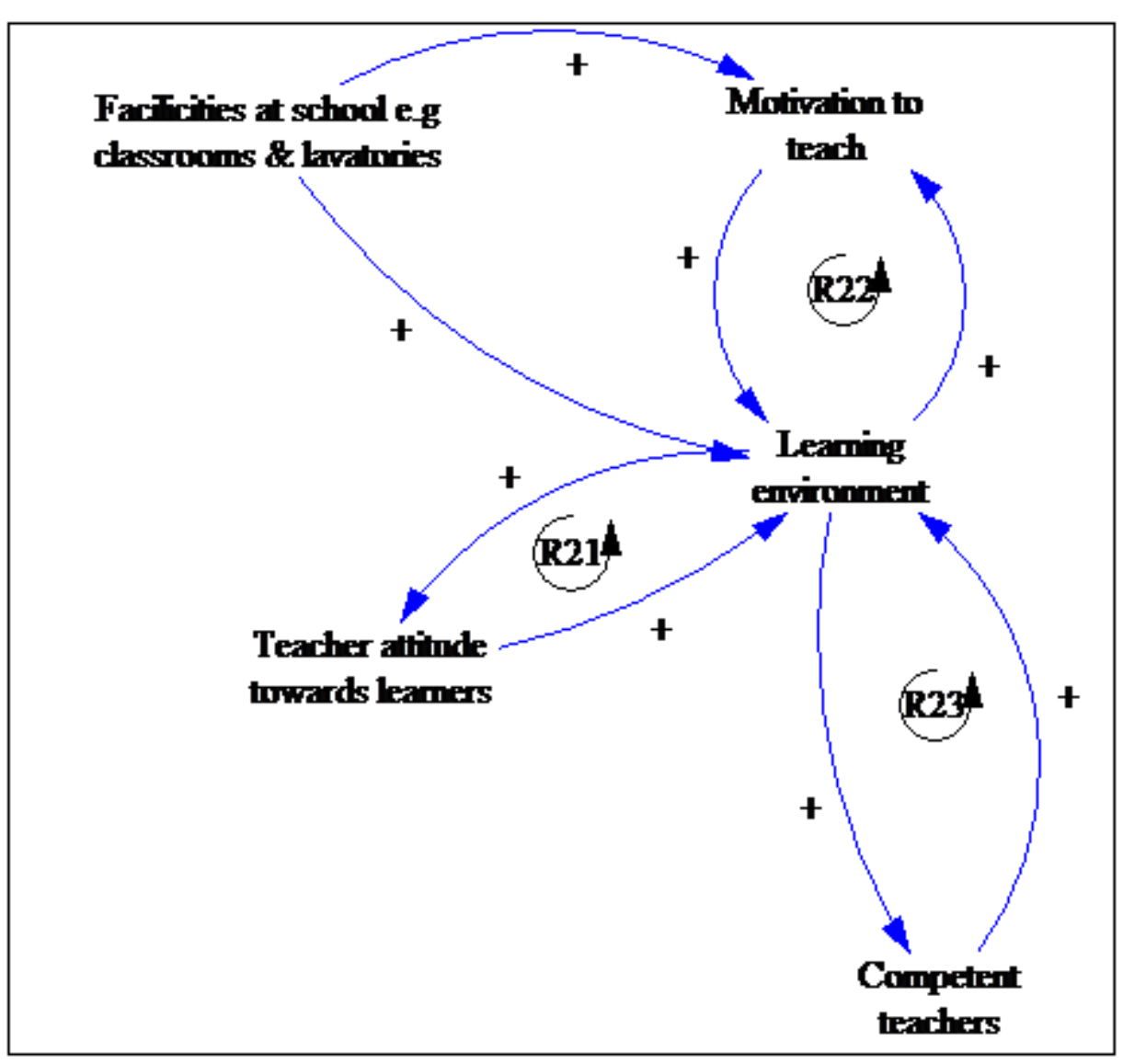

Figure 20. Settlement 4 caregiver context

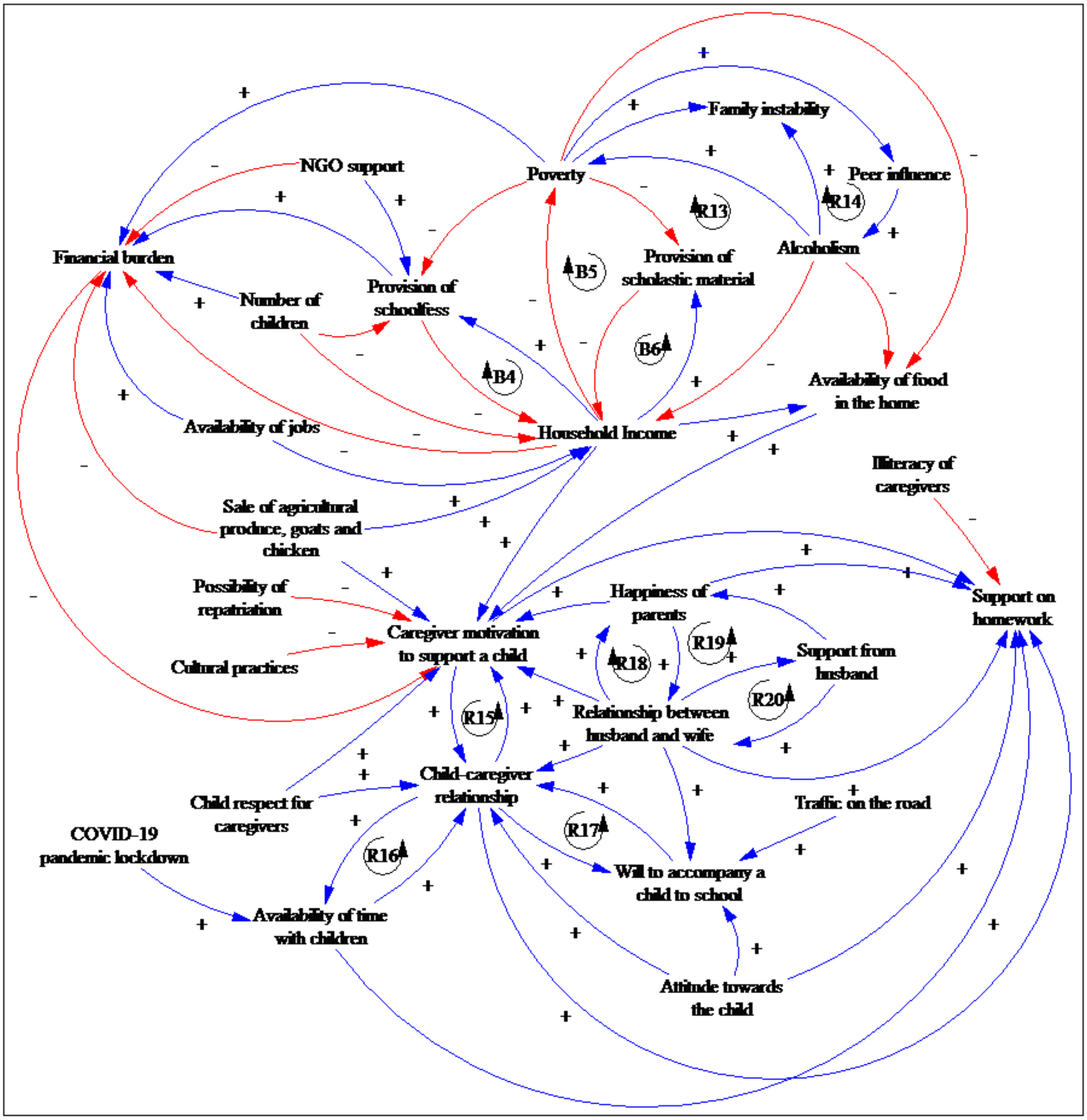

Supplement: Supplementary file 3 [file pone.0357215.s003.pdf]
