# Supplementary material for: Increasing school attendance and retention in refugee settings: Participatory development of the SchoolLinks intervention
Source: PLoS One. 2026 Aug 31;21(8):e0357215. doi: 10.1371/journal.pone.0357215 (PMC13528952; doi:10.1371/journal.pone.0357215)
Supplement: Supplementary file 4 [file pone.0357215.s004.pdf]

Supplementary materials: Action ideas from group model building workshops

Contents

Settlement 1 1
Caregiver 1
Children 2
Communication 2
Knowledge and attitudes 2
School 2
Settlement 2 2
Caregiver 2
Child 3
Communication 3
Knowledge and attitudes 3
School 3
Settlement 3 4
Child 4
Caregiver 4
School 4
Communication 4
Knowledge and attitudes 4
Settlement 4 5
Caregiver 5
Child 5
School 6
Knowledge and attitudes 6
Communication 6

Settlement 1

Caregiver

- Caregivers suggested that they should be provided with jobs as office cleaners to be able to buy soap to wash the children's uniform and be able to bathe.
- Providing food at home to avoid the adverse effect of hunger, which ranges from children not attending school, low motivation, and in the worst-case scenario, theft. Also, providing lunch boxes for school will play a lot in the child's motivation to learn so that they can bring food to school.

- NGOs should extend cash handouts for caregivers to engage in income-generating activities such as growing vegetables to finance household needs. Also, NGOs should provide uniforms and other scholastic materials.
- Caregivers should consider reducing child labour as a way of uplifting the child's motivation to learn and have time to do homework.
- Participants implored the concerned parties to plant trees used for timber in the communities as a way of creating employment to improve their household income to take care of their children's needs.
- Extending cash relief to caregivers to enable them to buy scholastic materials, clothes and pay school fees.
- Caregivers should provide allocated time for the learners especially in homework and other school related activities.
- Caregivers should be disciplinarians to curb the bad peer influence of their children to help them focus on class.

Children

- Food rations should also be increased, because what is currently being supplied is not enough relative to family size.
- NGOs should be involved in the construction of schools in the refugee settlements to help curb the problem of long travel distance for students and the large class sizes.
- Providing solar systems to help with the problem of darkness at night. Darkness hinders children from doing homework. Solar lamps can help bridge that gap.
- Government should provide adequate security for children in schools
- NGOs should ensure that the school materials they are providing children are equally distributed, as participants felt like not all students are receiving materials equally.

Communication

- Caregivers advocated for open dialogue with the teachers through invitations to school to be able to get feedback about their children's performance.
- Communication between the teacher and caregivers should be deliberately worked on to improve the relationship between them.
- Attending meetings at school to help with the child follow-up and build a strong collaboration with the teachers.

Knowledge and attitudes

- Conduct awareness campaigns in the communities to increase the value caregivers have for education.

School

- Improving the quality of education in order to improve performance
- Teachers should follow up with the children both at school and home more often
- Teachers should carry out continuous assessments to engage students and improve their performance. This in the end will motivate caregivers to support their children's education.
- Enforce education bylaws: defined as contextually specific rules established by the community or school to uphold education standards and expectations within the district.

Settlement 2

Caregiver

- NGO financial support to enable caregivers to buy animals like goats and chickens that they could raise and then sell to provide school fees, scholastic materials, and other basic needs to the children.
- Participants further urged NGO to extend support by providing scholastic materials and paying school fees for the children.

- Teachers suggested that the caregivers should appreciate the importance of spending quality time with the children to improve children's discipline and academic performance. A timetable can help create accountability and consistency.
- Government and other law enforcing agencies should better handle cases of (sexual) assault to allow the perpetrators to be held accountable. This will motivate the caregivers to continue supporting their children even after such situations occur.
- Government should offer soft loans (i.e., non-interest loans, where the amount is determined assessed on the person's ability to pay it back) to caregivers as a way of boosting their financial status.
- Free education for all refugees to lessen the financial burden of the caregivers. This will enable the caregivers to concentrate on providing other basic needs.
- Career guidance and counselling for the children and caregivers who have been traumatized because of early pregnancies/marriages, domestic violence, and alcoholism. This will build their self-esteem and motivation, especially for the children to stay in school, and for the caregivers to continue supporting their children.
- Provision of different education options like vocational training institutes to give practical skills to the children, especially those who are academically weak, disabled, or had dropped out of school.
- Caregivers were advised to equitably distribute children's time between doing domestic chores and schoolwork to enable their children's academic performance to improve. They further advised the caregivers to be actively involved in the children's homework and reminding them to read.
- Caregivers advised teachers to cater to individual differences of children, especially those with disabilities.
- Caregivers requested to be guided on how they should improve their relationship with their children. They confessed the lack of knowledge on how to achieve this, hence the need for help.
- Stopping domestic violence among families to help build synergy between the caregivers and subsequently support the children in unison.

Child

- Caregivers advocated having boarding sections, which potentially reduces the risks of having to travel long distances, but also it can substantially improve the performance of a child.
- Support should be given to child-headed families, since they have no adult caregivers to provide them with support.
- Provide lunch to students, so that they are not hungry, and can concentrate in class.

Communication

- The government should extend their support by paying the parent teacher association (PTA) fees to motivate caregivers to engage fully in the education of their children.
- Translators should support communications between teachers and caregivers when language barriers exist.

Knowledge and attitudes

- Create awareness campaigns on the value of education, especially on the side of the caregivers. This will change their attitude and become more involved in their children's education.
- Having termly meetings to get feedback about their children. This creates an avenue to strengthen the relationship between the teachers and the caregivers and helps in following up on the child's performance.

School

- Government involvement by improving the quality of the teachers to enable good performance of the students. This can be achieved through refresher training courses.
- Construction of classrooms will create more space/larger class sizes for children to attend school.
- Enforce education bylaws: defined as contextually specific rules established by the community or school to uphold education standards and expectations within the district.

Settlement 3

Child

- Talk to children about value of education
- Children to be put in boarding section to curb the risks associated with moving long distances
- Construction of more schools to reduce the distance
- Girls should be given training on how to use reusable pads. More support for girls with sanitary products
- Dropouts should be enrolled in vocational training institutes to gain some practical skills. This motivates the caregiver to support the other child knowing that there is an option in case of dropout.
- Community meetings should be organized to discuss how to handle the children who behave poorly.
- Teachers must encourage the children to come to school
- Teachers, caregivers, and NGO should offer guidance and counselling to the children on sex education.

Caregiver

- Caregivers should provide food for the children
- Alcoholism should be stopped to reduce on unnecessary expenditure
- Children must be stopped from moving at night to go to nightclubs
- NGO support with payment of school fees, timely provision of scholastic materials.
- Caregivers should provide shelter for their child
- Caregivers can buy pens for the child (not a huge cost)
- Both the Caregivers and teachers should love the child
- Caregivers need support like radios, to have access to educational messages
- Caregivers should ask their children about homework, and encourage caregivers to do the same – caregivers could act as role models to other

School

- Creating a conducive environment for the learners, such as reducing corporal punishments through engaging the caregivers in proper disciplining of a child.
- Inclusive education, which involves equal treatment of learners regardless of religion, tribe, colour, and physical appearance.
- Home visits by teachers
- Enhance teacher capabilities / refresher trainings by supporting partners, i.e. NGOs.
- Better facilities at school (like classrooms and lavatories) would increase motivation to teach and learning environment.

Communication

- Caregivers could be taught English.
- The school should speak the languages of the community.
- The need to have translators at school to help deal with the problem of language barriers.
- Community members could be hired as translators.
- Regular PTA meetings to for teachers to discuss with caregivers how to support their children's education.

Knowledge and attitudes

- School meetings need to be organized to tell caregivers how to support their children's education.
- Trainings and workshops to increase the caregivers' value of education.

Settlement 4

Caregiver

- Family members should engage in family meetings to encourage their children to study. The participants believe that this strategy will be easy to implement and will have a low impact since the responsibility of studying rests solely on the child. The caregivers' role is to guide and provide the necessary support a child needs such as school fees, scholastic materials among others.
- Caregivers should escort their children and perhaps their neighbours' children to school. This will allow caregivers to be updated on their children's grade and enrolment status, as well as allows the school to collect and update contact details of the caregiver, increasing communication between school and home. This communication will also clarify whether the child should be enrolled in AEP or mainstream.
- NGOs should help with fencing the schools to deal with the problem of insecurity. Caregivers can provide the poles.
- Stopping alcoholism among caregivers by informing about the dangers of alcohol. Elders and church leaders can do this.
- To reduce family breakdown, having counsellors talk to husbands and wives to help resolve marital problems amicably.
- To curb poverty and increase household financial spending, caregivers should be encouraged to engage in agriculture, small business ventures, and training in poultry keeping. For agriculture, food can be grown for both subsistence and commercial to deal with the food insecurity problem.
- Forming associations of disabled caregivers so that they are equipped with practical skills like tailoring, shoe making among others. This can be done by the NGOs, government, and political leaders.
- Caregivers (husband and wife) should discuss and jointly make decisions to live harmoniously in order to balance engagement of children in agriculture and home responsibilities, as well as school fees and basic needs.
- Provision of scholastic materials by the caregivers and NGOs.
- Ensuring that children stay at home to avoid joining bad peer groups.
- Support in drilling boreholes to provide water to wash uniforms.
- Caregivers check that their child is doing homework during the evenings, even if the caregiver is illiterate.
- Caregivers can start by asking one question per day, like what their child studied in school that day.
- Caregivers organize children into study groups within the community, so that children can ask each other questions about their homework and learning.
- Parents should escort the child for registration in school every year, so that they know which class the child is enrolling in. It is also an opportunity for the school to collect and update the contact details of the parent, which would help communication between the school and home. It will also address the issue of children flitting between AEP and mainstream, which the teacher reported as an issue – children enrol themselves in AEP as it's only 3 years, but then find the content too hard/fast-paced and return to mainstream. However, they will have missed the relevant academic content and therefore fail their exams, which leads to them repeating the year, or dropping out.
- Encourage monthly payment of exam fees by parents through the school management committee (SMC). Explain to parents how this money is used, to increase transparency.
- Parents can help with slashing the bushy areas around the school, to support the learning environment for their children, and increase security (e.g. from snakes)

Child

- Free education for all the refugees. This support can be through NGOs and government agencies since most refugees are vulnerable and lack the resources to provide for their children. They believe that this strategy is hard to implement because it involves many financial resources. Once implemented, it will create a high impact among the refugees and the community in which they are living.

- Policies to support young girls' education. Most young girls are exposed to many risks such as rape and defilement, which ruins their chances of further studies. This strategy is hard to implement as all the stakeholders must be involved and might require putting in place some policies to be implemented. The participants believe that once implemented, it will keep young girls in school and have a high impact on the community.
- Provide psychosocial support to children with mental issues. Increasing psychosocial support to children with mental problems is an area that requires intervention. The participants agreed that this support is difficult to implement as it requires specialists to be hired to handle the cases. Therefore, it requires high effort but can impact highly on the children with mental issues.
- Family planning practices should be introduced in the camps to deal with the problem of early pregnancies and reducing on family size.
- Caregivers and teachers should provide guidance and counselling to the children.
- Caregivers should actively discipline their children to reduce on moral decay.

School

- Construction of more classrooms to support large class sizes. In most settlements, classrooms are few and accommodate a big number of learners. There is a need to decongest learners within classrooms, especially during the COVID-19 pandemic where social distancing is observed. The strategy is hard to implement because many resources are required and it is a long-term plan, which may not be resolved sooner. If the strategy can be implemented, it will create a high impact for the learners and other stakeholders.
- Recruitment of teachers to address teacher-to-learner ratio. There is a need to employ more teachers so that teachers can pay attention to learners' performance in class. The intervention is hard to implement, as it will require an appropriation of the national budget to cater for such recruitment exercises.
- Caregivers and community members volunteer to support teachers in classrooms, rotating on a weekly basis so as not to significantly impact the individual's income-generating activities and other responsibilities. This could be further discussed at the PTA meetings.
- Construction of secondary schools with boarding sections. The participants mentioned that most of the schools are primary schools and are located far from settlement camps making it difficult for their children to commute to and from school. Such long distances to school demotivate the learners, especially during bad weather. It becomes very difficult after completing form seven when joining secondary school. They still emphasize that secondary education is very expensive for them to afford. Their view is that such undertaking is hard to implement but can create a high impact because it will provide more opportunities for the learners to obtain high-school education.
- Construction of more classrooms especially in lower primary, that is primary one to primary four.
- Recruiting more teachers in the lower sections.
- Inclusive education especially for children with disabilities and chronic sickness. This motivates the caregiver to support a child if equal opportunity is given.
- Teachers' salaries should be paid on time.
- Specific days should be organized to celebrate teachers.
- Absent students should receive follow up in a systematic way (potentially through the PTA or caregiver volunteers).

Knowledge and attitudes

- Adult education programs should be offered to caregivers so that they can better realize the value of education. Support and advocacy for the programs can be offered by NGOs, local councils, or church leaders.

Communication

- Teachers and NGOs should engage caregivers in how they can best guide and support their children
- Schools should be transparent in their communication of exams fees to encourage caregivers to pay them.
