# Supplementary material for: Increasing school attendance and retention in refugee settings: Participatory development of the SchoolLinks intervention
Source: PLoS One. 2026 Aug 31;21(8):e0357215. doi: 10.1371/journal.pone.0357215 (PMC13528952; doi:10.1371/journal.pone.0357215)
Supplement: Supplementary file 5 [file pone.0357215.s005.pdf]

SchoolLinks strategies
Validation workshops and discussions

Contents

Validation workshop 1: [Settlement 1] 3

1. Rewards for high attendance 3
2. Homework support group 4
3. Caregivers accompanying for enrolment 5
4. Volunteer support to teachers 6
5. Caregivers/older children escort children to school 6
6. Income-generating activity 7
7. Hiring volunteer as translation 8
8. Topics for discussion in school 9

Validation meeting – [settlement 2] 11

1. Attendance rewards & recognition 11
2. Homework support 12
3. Caregiver enrolling children 13
4. Classroom assistants 14
5. Escorting children to school 15
6. Income-generating activities 15
7. Volunteer translators 16
8. Topics to discuss in school 17

Validation workshop: [settlement 3] 20

1. Attendance rewards & recognition 20
2. Homework support groups 21
3. Caregivers enrolling children 23
4. Classroom support 24
5. Escorting children to school 25
6. Income-generating activity 26
7. Translation support for parent-teacher meetings 27
8. School meeting topics 28

Validation workshop: [settlement 4] 31
1. Attendance rewards & recognition 31
2. Homework support groups 32
3. Caregivers enrolling children 33
4. Classroom assistants 35
5. Escorting children to school 36
6. Income-generating activity 37
7. Translators for parent-teacher meetings 38
8. Agenda topics for parent-teacher meetings 38

Validation workshop 1: [Settlement 1]

22nd February 2022

Should add to causal map:

- Distance to school
- Child disability as reason for low attendance
- NTS: Within Teacher-Caregiver communication, add ‘Feedback from caregivers to school’
- Cultural beliefs related to early marriage – selling young children while they’re virgins

Feedback on causal map:

- From Caregiver: He has a low education but supports the education of his children. However, the facilities at the school are not good but he is unable to express himself to the school management due to the language barrier between him and the school staff

Note: strategies 1-3 were discussed as a group and then separated into caregivers and partners/district actors

1. Rewards for high attendance

- One person felt the products are not relevant.
- It will be difficult for sustainability
- Instead of Strategy 1: Teacher should enquire to children and caregivers why each specific child had low attendance, or gets to school late
- Instead of Strategy 1: Termly recognition and praise for attendance – will encourage

How will we choose which children are rewarded?

- Children with special needs and orphans should be prioritized for rewards
- Should take into account the categorization of learners, i.e. fast, middle and slow learners

<u>Teachers</u>

Easy to implement and high impact

- Would have impact on children’s motivation, also boost self-esteem
- Create competition amongst learners and increase morale
- Sanitary products would help girls a lot. They would have to be sensitised on how to use them.
- Boys need specific things too, e.g. underpants
- Soap is good, would improve hygiene
- Notebooks – should be attractive ones
- Should reward the 1st to 3rd child and the lowest child
- Children with disabilities should be rewarded
- Extra criteria: punctuality and active participation
- Do extra rewards for those who increase a lot

- Could cause complaints from children about not getting rewarded
- Needs to be sustainable (process and funding)
- Should be termly and accompanied by sensitisation

Recognition: Easy to implement and medium impact

- Would work; one teacher did this with handwriting in her class and the class showed improvement

2. Homework support group

<u>Partner/district feedback:</u>

- [participating organisation name] did something similar and it worked
- Add as outcome: social benefits between children, and between adults too
- [participating agency] very positive about this idea
- Concern: location for the group, as it could be too many for one home

<u>Caregivers</u>

- Good idea though same concern about venue
- Issue: timing – schools stop at different times (note: children in a community may go to different schools)
- Some schools don't give homework so not applicable
- Would need alignment between the home and the school
- Risk: Caregivers may want to be paid

Q: How often should the groups be organised?

- Daily (on the assumption the HW is daily)

<u>Teachers</u>

High impact, difficult to implement

- Very good idea
- Would require facilitation to caregivers
- Good but challenges, like low caregiver education – this will be a big difficulty
- Volunteers who are educated should be selected
- Idea is good, caregivers need sensitisation
- Will be more effect if someone is assigned
- Homework:
    - P5-7, the frequency depends on teacher. In most cases, it's given on a Friday. Catch up time from class (?)
- Two caregivers could do it together if one is not educated
- Needs deep sensitisation (mentioned by many) to follow children's progress
- Should be caregivers' agreement

- Teachers should guide at the beginning so that it would be more sustainable, e.g. on how to form the group and how children should do homework
- Caregivers may fear missing children
- Could rotate between caregivers
- Competing priorities after school so would be difficult to engage a group at the same time
- At holidays or at the weekend would be better. Provide materials.
- Call the caregivers of children who don't do their homework
- Would be high impact because it would be felt and seen by caregivers

3. Caregivers accompanying for enrolment

<u>Partner/district feedback:</u>

High impact, high feasibility if teachers. Low if volunteer.

- Good idea but difficult if volunteers are not paid.
- The Refugee Welfare Committee (RWC) and Secretary for Education should be included.
- [participating organisation name] – this is already happening but it should be made mandatory
- Teachers register the children for enrolment – <u>use teachers</u>
- It will initiate communication between community and school

NTS: Instead of buying a laptop/desktop, could use work laptop and print sheet for school, and update termly

<u>Caregiver feedback</u>

High impact, easy to implement

- I can give my contact details etc. but then leave for South sudan or to places far from West Nile, without a phone – what then?
- [research coordinator] explained that they can give the contact details of another caregiver who will remain in the household
- Concern: what about for the children who are not their biological children?
- Volunteers could be anyone educated in the community, but it would need to be facilitated.
- Really liked the idea

<u>Teachers</u>

Easy to implement and high impact

- Good – teachers can register the children
- Registration is hectic, a lot of parents come
- The parents would need to be empowered (? Meaning facilitated?)
- Name issue – different names at home vs. those on registration ID
- Caregivers need to give the child's name to correspond between card and school register

- Changing a child's name on the card is really difficult but very important for verification – use this rationale for sensitisation
- Really good idea – children who come alone promote themselves. Teacher and caregiver should decide together, and this would strengthen the teacher and caregiver relationship
- Best action idea if done in November and December would increase the school's knowledge of child's needs
- Enrolment is done termly – could also catch dropout
- Easy if teachers are trained in advance

4. Volunteer support to teachers

Partner/district feedback:

High impact, difficult to implement

- Won't work if they are not paid
- 'classroom assistants' are individuals currently used, and they also help with translation
- There could be an issue of language – many languages spoken in the class, the volunteer may not speak them all

Caregiver feedback

- Like the idea of someone from the community but would need to be facilitated

High impact, difficult to implement

Teachers

Easy to implement and high impact

- Very good idea (said by many)
- They already encourage parents to come and see the classes – see learning, participate
- Volunteers – form a teacher learning circle where they support each other
- Good, parents would do it – need to be educated, at least have finished S4
- Teaching assistant role – support controlling the class and do translation
- Need to be facilitated (as teaching assistants, not as teachers)
- If highly motivate/highly upgrade (?) and volunteers to support extra TAs

5. Caregivers/older children escort children to school

Partner/district feedback:

- Good idea
- Must consider the age of the children – for P1 & P2 it should be done
- Should also do this for children who are vulnerable
- Girls and boys generally go separately – if you use older children, must identify one of each gender to be responsible

- They can go in groups, no need for a caregiver

Add as outcome: increase punctuality

Caregiver feedback

- A lot of discussion, very different perspectives
- If it's caregivers escorting children to school, it's easy to do but there's no motivation to do that so implementation is difficult
- If caregivers are motivated, it will be easy to implement
- Primary school children need breakfast before leaving but older children take porridge at school. Therefore if the older ones wait for the younger ones, they'll be late for class which will affect their performance.
- In the community, some children are attending different schools so neighbouring children may not go to the same school. So the caregiver may need to do some kind of mobilization, and/or take their child to another household. This will also delay the other child from going to school.
- Young children are slow which makes the older children late at school – the older child is held accountable, which could affect his/her motivation to go to school as they're getting in trouble for another child
- Some children have bad manners and are stubborn so you may not want to trust your child with them

High impact but difficult to implement.

Teachers

High impact, difficult to implement

- Good but grouping would be difficult as some children go early and don't want to delay
- Will improve on punctuality of learners
- Different times for classes so grouping should be done by class and location
- Establish 'collection points'
- Implementation will be hard
- Caregivers might find it tiresome
- Good but caregivers need to be sensitised of objectives – "from the root to the leaves"
- To be effective, needs to coordinated by community leaders
- It should be led by implementing organisation until it is smooth – a transition

6. Income-generating activity

Partner/district feedback:

- Good idea but not realistic/possible to implement
- Would need multiple types of income-generating activities for it to work

- [participating agency] calculated the amount of money it would generate per month – 4800 UGX, which is what each woman (in a group of 12) would take home yearly. Uniforms are at least 15000 UGX. Not financially feasible at all.

<u>Caregiver feedback</u>

Easy to implement and high impact

- E.g. burn charcoal and cut timber, small business, sell food ratio,
- Very much like the idea

<u>Teachers</u>

High impact, difficult to implement

- Very good idea (said by many)
- The activity needs to be contextualised
- Women's group – investment and interest
- NGO needs to ask for interests first
- Collective resources is powerful
- A private school organised this to generate 1000 UGX for children most in need
  - Needs to be attached to school

7. Hiring volunteer as translation

<u>Partner/district feedback:</u>

- Would have high impact but a challenge if they're not paid.
- Classroom assistants could do this
- [participating agency]: Is a context-specific issue, depending on where the teachers are hired from

<u>Caregiver feedback</u>

- Would need to be facilitated
- Why doesn't the translator be a member of the PTA during PTA meetings. Currently PTA members are given sodas and facilitated for transport.

Easy to implement and high impact (if facilitated).

Translator fee: 10-20,000.

<u>Teachers</u>

Easy to implement and high impact

- Already done by teaching assistants and community members, very available
- Very important – makes the caregivers feel involved
- NGOs often get translators from the school (they facilitate them)

- One person with many languages
- Call class meetings – TA helped with translation
- 3 parties: English, Arabic and both
- Timing is a challenge as it extends meetings

8. Topics for discussion in school

Partner/district feedback

- All good talking points
- UNHCR, OPM, partners and PTA discuss what is needed vs affordable and decide school fees
- Should also share information on the assets of the school (e.g. desks, benches, facilities) and the number of children, for total transparency
- Additional topic: discipline in the school, of children and teachers

Caregiver feedback

- All great topics
- Especially want to know about the financial expenditure
- Role models – doesn't favour illiterate parents, concerns for how it would make them feel. But it would inspire the children
- Help to monitor how fees are being utilised
- Currently, the OPM, UNHCR, partners and PTA decide together on the fees. The school shares their needs. The caregivers (especially refugees) do not see it as their responsibility to pay.

Easy to implement and high impact

Teachers

Easy to implement and high impact

- Good ideas (mentioned by many)
- Need school accountability otherwise caregivers want to transfer children to another school
- Increase caregivers' knowledge
- Point 2 – will make caregivers feel engaged (mentioned by many), ideas used and participation

Add:

- How can we feed children at the school? This would be really good for attendance.
- What topics are taught at school

Additional action ideas:

Partners

- Provide or sensitise on reporting lines for teenage pregnancy

- Increase the knowledge of children and caregivers on children's rights, child safeguarding and child protection ('tribe club'?)
- From [participating organisation]: Address menstrual hygiene
- School feeding programme
    - Parent-led, high impact
    - Parent gives food in-kind, calculated based on the number of children. Some of the grain is sold for other ingredients. The Partner pays for the cook.
    - But families are provided with 8-9 kg of maize for one month -> would not leave enough food for the household

<u>Teachers</u>

- Teacher welfare is key
- Regular salary is the motivation to follow up children
- Teachers in crisis zones – packages for support, e.g. 13th month and welfare support

Validation meeting – [settlement 2]

1. Attendance rewards & recognition

Partner/district feedback

- Depends on age of children
- Girls, especially teenagers, face challenges – they can be disturbed by men and lack of sanitary products
- Good idea because there are decreasing funds from partners – move towards self-reliance. Though caregivers may take the products – the home-school relationship is important to prevent this. Need to explain to caregivers about the reason for rewards.
- Especially needed for more vulnerable, e.g. orphans
- Suggestion: provide to all children
- Should prioritise based on needs, e.g. children with disabilities – reward based on needs
- Would increase motivation of children and caregivers.
- Need community structures to oversee, and involve child protection and refugee welfare structures.
- Needs long-term commitment and sustainability

High impact and easy to implement but difficult to sustain

Caregiver feedback

Easy to implement, high impact

- But the teachers should give rewards
- Concerns over how many rewards
- Should be tied to other things like smartness (dress code) and timely coming to school

Teachers

High impact, easy to do (recognition)

- This is already done at the end of the school year, though doesn't have much impact – perhaps not done frequently enough
- More frequent would have more effect
- Requires commitment
- Would increase motivation and address barriers to attendance
- If some start to improve, many others will too. Books motivate children.
- Sanitary products for girls would increase morale and reduce dropout due to shame
- Soap for boys too
- Would reduce dropout (said by many)
- Children come without books
- Should be done termly and weekly
- Would create positive competitions

- Keep the materials at school

2. Homework support

Partner/district feedback

- Good idea, peer-to-peer is a good way to learn
- Home are next to each other, e.g. people of concern are grouped together
- Influential members of the community should be used
- Also could improve communication between neighbours
- Some caregivers may feel that they don’t need to monitor children, and uneducated caregivers may not be able to help
- Should be done one-to-one to make a strong relationship between child and caregiver
- Can include literacy games
- Should be done on a regular basis
- Some caregivers don’t value education
- Seeing children being supported motivates the caregivers
- Need to avoid creating conflict based on child selection
- Children should not become bosses
- Older children could support caregivers with low/no education
- Use local language, though language barrier between groups could be an issue
- Do it daily or every couple of days
- Competing priorities, e.g. collecting water, caring for goats etc.

High impact, difficult to implement

Caregiver feedback

Easy to implement and high impact

- They would identify which home in the community has shade (e.g. big tree)
- It would build friendships
- It would encourage the slow learners to catch up

Teachers

Low impact, difficult to do

- Caregivers who have gone to school will do this but uneducated won’t
- Children will benefit
- Depends of the relationship between community members – if issues, idea will not work – example of radios
- Idea is ok but community feels that education is the school’s responsibility
- Educated parents will support
- This is a new initiative. Traditionally older children help the children
- Good idea – cooperative learning

- Will increase self-esteem
- Normally give class work in groups – slow learners learn from their friend
- Would increase active participation, especially for the ones who are quiet in class. Would increase socialisation and teamwork
- Would build friendship and shared experiences
- Homework is given every Friday
- Should be rotation between caregivers
- Caregivers can also help children to understand
- It will take time out of other responsibilities
- Depends on time during the day – everyone has different schedules

3. Caregiver enrolling children

Partner/district feedback

- Good idea because the caregiver get to know teachers and the school\very good – get to know the school and risk factors for the route to school and the school environment and security
- Very good, especially for children with disabilities – caregivers can inform teachers/school on how to handle children and of their psychological state, and effects of certain behaviours (e.g. being shouted at)
- Able to addresses challenges from the school
- Good for security, child protection and child safeguarding
- Helps integration of the child in the school, the child (and caregiver) can be shown around and introduced to the head teacher and other staff
- Classroom assistants could help
- Need careful selection of teachers, i.e. not one who has conflict with other caregivers
- Would improve discipline of child as they know their parents can be contacted

High impact, easy to implement

Caregiver feedback

Easy to implement and high impact

- The teachers are sometimes few in the school so they may need a volunteer to assist with enrolment
- This volunteer should come from the community
- The volunteer should be rewarded with soap/good in kind or a PTA member (could be 10-20,000)

Teachers

High impact, easy to do

- Good idea, very important for caregivers to do this
- Children are often unaccompanied, even when just 5 or 6 years old

- Registration is a challenge, often don't come
- Moving between AEP and mainstream is a very big issue
- Is an opportunity to learn about the children, for example disabilities, orphaned, chronic illness (mentioned by many)
- The parents also need to be present to pay the fees
- Could address the issues of conduct and absenteeism
- Should be done at the beginning of the school year
- Registration is done in the deputy headteacher's office. Would be easier with a laptop. Registration data always needs to be handed over to OPM – digitising would save a lot of time and prevent the records from being lost.

4. Classroom assistants

Partner/district feedback

- Would help with fighting, a big help for classroom management
- Improve teacher-caregiver relationship
- Need for interpretation for sign language for children
- Can increase inclusion, if considered during the recruitment of volunteers
- ECW is doing something similar
- Would need to be facilitated and someone who is considered a good example and someone educated
- Facilitators – check for CWTL who these people are
- Training exists for classroom assistants – could recruit from that programme
- Even difficult to recruit individuals (? Don't understand this)

High impact, difficult to implement

Caregiver feedback

High impact, difficult to do

- The volunteer requires payment and a certain education level that may not be available in the community
- The volunteer should speak the language spoken by people in the community

Teachers

High impact, easy to do

- This is one of the biggest challenges
- PTA paid teachers (who finished S4)
- Still need to be trained/oriented
- Especially needed for P1 and P2
- Will help – there are so many learners so having another adult to just help control the class will have a big effect

- This has been done elsewhere and works
- P4 – an especially difficult class because it's when it switches to English, translation would benefit
- P5 and higher, translation not needed
- Need clear role and responsibilities
    - Behaviour control, support with games and extra-curricular activities
    - Support with marking
- Possible to find in the community but needs to be paid. E.g. 300 000 UGX

5. Escorting children to school

Partner/district feedback

High impact, easy to implement

- Perfect – caregivers should do this
- Or hire a boda, a trusted one
- Children aged 3-6 should always be escorted
- Group is better than older children
- Challenge is returning home

Caregiver feedback

High impact but difficult to implement if it's the parents taking children to school every day

- Difficult to do on a daily basis as parents are engaged in other activities for the family, e.g. farming, especially in the morning (before the sun gets really hot)
- Elder children would be easily able to do this but parents should follow up their children at school once per week (i.e. go to the school)

Teacher

High impact and easy to do if grouped but difficult to do if accompanied by caregivers

- Children come at different intervals
- Would work if grouped by village
- Used to do this and it worked
- Nursery children are accompanied
- Caregivers should escort lower classes, this will increase attendance
- Will also reduce accidents on the way to school if they are accompanied

6. Income-generating activities

Caregivers

High impact, difficult to implement

- would require training on income-generating activities and they do not have start-up resources
- would not sell their food items to get capital because prices are very low

Partners/district feedback

Strategy was not discussed due to time pressure

Teachers

Low impact, difficult to do

- Not enough maize if given to do this activity
- PTA = 2000 UGX per term

7. Volunteer translators

Caregivers

High impact, easy to implement

- the person needs a token of appreciation/facilitation
- the person should come from the community

Partner/district feedback

High impact, easy to implement

- Good idea
- Volunteers need to be able to speak multiple language and must be good at communication and speaking to large groups
- Could be same person as the classroom facilitator
- Can ask at the meeting, the community will know who's good and capable
- Should be rotated
- Should be two translators, to share the task and do quality control
- Should be from local community and would a position of pride
- People who could play this role are already identified and represent the community in other meetings
- Would increase mobilisation and parent attendance, as long as the volunteer is liked

Teachers

High impact, easy to do

- Ok idea, will have high impact
- At the moment, teachers translate
- Pastor appoints people for translation from the community
- Adults are critical about errors in translation
- In PTA meetings, the community does translation
- Is time-consuming
- Need to be careful and it is tiring

8. Topics to discuss in school

<u>Caregivers</u>

High impact, easy to implement

Exam fees

- wonderful topic because they really want to know this information
  - if there is some money missing, this would allow parents to know and they could support with closing the gap
- if there were an excess of money, they could be involved in deciding what the money is spent on
- want to see the outcomes of what the money is spent

School development and needs:

- Also a good topic and parents would be willing to come in and give their contribution in kind

Information on how caregivers can support on children's learning

- Some parents don't even know that it's important to give their children time to do homework
- Some do not even know how to help or engage with their children because they're illiterate
  Gave example: they don't know if their children are taught or not - just see ticks in a child's book and they don't know what it means

Role model give inspiration talks

- Would inspire the children to be like the role models
- Difficult because the children don't have any role models in the community - only have teachers as inspiration
- Unless they're sick, they don't really see a nurse or doctor
- They should see people in other professions

<u>Partner/district feedback</u>

High impact, easy to implement

- Improves school accountability
- Fees not accounted for causes parents not to come
- Members of the community who have gone to university could be role models
- Role models is a great idea – could create healthy competition between villages
- These are the exact topics that caregivers want to discuss at meetings with the school

Topics to add:

- Girl child's life, senior woman teachers
- Update on child performance

- Future education opportunities, e.g. vocational
- Child's rights and child protection
- Updates on change to the system, e.g. curriculum
- Referral processes, also for domestic violence
- Roles and responsibilities of the school and caregivers
- How to be supportive of innovation and strengthen the skills of children
- GBV in school and bullying – how to deal with these (joint response)

Other feedback:

- Children are demotivated because at the end of P7 there is no secondary school – the neighbouring school is kms away – they're afraid to move there/travel there – so there is nothing to work towards, no inspiration because there is no continuity, no assurance.
- For the best performing children at P1, is there a possibility of sponsoring a child for secondary school. This would inspire other children to come to school.
- There is need for more replacement of transferred teachers. For example, 2 teachers were transferred from Eden and have not been replaced. So children who are supposed to go to school all day stay home after lunch because there are no teachers. They also see other schools where children study all day, and are demotivated because they don't have teachers.

<u>Teachers</u>

High impact, easy to do

Exam fees

- Good idea, for example to explain the exam support fees – transport, marking, brining teachers together each term, P7 monthly exams.
- The school compromised – exam support fees are not compulsory
- Monthly exams really help but require the fees

School developments and needs

- The PTA gives development funds to the SMC. Was 1000 UGX per child, now increased to 2000 UGX. Covers repairs and maintenance of facilities and teachers' home repairs. This had to be explained to the caregivers.

How to support children's learning, e.g. giving time for homework

- One teacher did this and it worked; every child in the class passed their exams

Role models

- Role model should talk about behaviour. Some children are scared of teachers, so would help with this, e.g. chairman
- Used to invite RWC (refugee welfare committee) 1 & 2 to talk to P7 towards their exams

- Female role models have also been invited to speak during assembly, and they really inspired the girls

Topics to add

- Guidance and counselling – to align the school and the home on behaviour and learning, so that both sides are saying the same thing
- Bylaws – are made by the RWC in consultation with the school. For example, caning children if they go to disco or to funerals (at night, dancing etc)
- Feeding children should be discussed. Parents could send water containers and pancakes. Issues of water – it only came in the afternoon.

Validation workshop: [settlement 3]

1st March 2022

1. Attendance rewards & recognition

<u>Caregivers</u>

High impact, easy to do

- Very good idea
- Would motivate the children – said by many
- Would reduce attendance barrier due to lack of items
- Increase more effort of sending children to school, and of children to go to school. Especially for girls because of sanitary pads
- Difficult if requires schools to pay but government and partners can do it together if well planned
- These items have been promised for a long time in government schools but never materialised
- Recognition instead of rewards:
    - Very possible, would still have good impact
    - Do it during assembly
    - Not as influential as rewards – said by many

<u>Partner/district feedback</u>

- Recognition for high attendance is a key factor in motivating and improving attendance
- The rewards are not sustainable when the when project ends and this will make it hard to replicate
- Boys should be rewarded with underpants/boxers
- Rewards can also include mathematical sets as many children skip classes when there are lessons that require use of these instruments.
- Some children have been provided with these rewards and still failed to attend school.
- Recognition should be in class/ stream and then overall done on assemblies
- Attendance lists showing children with high attendance should be pinned in classes
- Children of lower classes can be rewarded with stars (stars placed against their names on lists pinned in class)
- Parents whose children have high attendance should be recognized during PTA meetings (can be rewarded with a bar of soap)
- There are also teachers who are rude and rough with learners causing them to avoid coming to school.
    - This is easy to implement when rewards are not gifts but rather recognition and the impact would be high.
    - If rewards are to be given, it would be difficult to implement though impact would be high

Teachers

- Learners who are absent due to lack of sanitary products will be supported to attend school
- FCA provides sanitary products to girls but they are very few and don't cover all adolescent girls
- Providing the material rewards will reduce the burden on parents
- The rewards like soap, sanitary products will improve hygiene of these children
- The rewards are gender sided and the boys are not catered for. So, rewards like boxers or underpants can also be included for the boys in addition to knickers for girls
- The rewards can be given monthly or termly
- Recognition for high attendance should be done per stream and class and overall per class on assemblies as this will increase the motivation of learners to attend school
- The Ministry of Education burned holding of assemblies in schools due to covid-19, the assemblies being held at the moment are illegal (against the MoE directive)
- For classes where attendance is very high, the teachers can decide on who to reward based on discipline, response to bells in school/time management
    - This strategy would be easy to implement and it would be of high impact

2. Homework support groups

Caregivers

High impact, easy to do

- This will work – children learning from different levels of ability from different schools in the community, children will help each other
- High impact, will enable children learn from each other, celebrate each other's achievements as a group, increase bonding and motivation
- Easy to do and will improve education
- Issue is to bring children from far distance
- Many children have issues for exams when there is no one to support them
- Frequency: twice per week (two supported this)//daily//once a week//monthly

Partner/district feedback

- Liaise with the teachers to give homework as 'group work' rather than individual work.
- The group should not exceed 10 pupils if they are to learn and benefit from each other
- The teachers can be essential in formulating these groups so that the slow learners in class benefit from this initiative
- This will increase confidence of some learners
- This will increase friendships among children as they will learn the benefits of cooperation at a young age
- It is not possible to rotate homework groups as some caregivers are not willing to host these children

- There are households in the community that hosted the learning sessions during lockdown, they can still be used for this purpose
- The teachers should be able to follow up/supervise these groups at least once a month
- The group work can only be done on weekends as it will be difficult to implement week days due to competing priorities of households when children return from school
- This will help children to check up on each other/group motivation and they're able to follow up their colleagues that don't attend regularly
- The caregivers need to be sensitized about the importance of group work or allowing their children to participate in the group work on weekends if this is to work
- Sensitization should not be left to one partner but all partners and the community structure including religious leaders, VECs, PTA, SMC, RWCs etc should be involved
- Caregivers can be mostly targeted in churches
- The PTA, SMC members can be essential in creating awareness among caregivers
    - This will be difficult to implement and have a moderate impact

<u>Teachers</u>

- The caregivers need to be sensitized about this strategy first so that they're able to support the learners in their groups; and also give these children time to meet and do the homework
- This would reduce redundancy amongst children who loiter in villages over weekends due to lack of what to do
- The children will be able to learn due to peer influence
- There's increased group motivation to learn as weaker children are supported by the strong ones as children interact freely
- The children will be able to express themselves in the local languages in groups and this will make understanding of concepts much easy for them
- Caregivers will be able to recognize scholastic materials their children are missing and provide them
- Children without some materials like mathematical sets will be able to share with those that have and feel supported in getting their homework done
- In case the children fail to answer certain questions, they can be able to consult the caregiver of teacher as this foster increased urge to learn among children
- Children in lower classes are not given homework due to the class sizes. But with this strategy, group homework can be organized and given one a week (Friday), for children to do over the weekend
- Upper classes are given homework daily
- The groups can be organized to meet twice or once a week to do this homework
- The children can be organized in groups of 5 to 10 if they're to benefit from each other
- The teachers would need to monitor these groups at least twice a month to ensure that they are achieving the intended purpose
    - This strategy would be moderate to implement and have a high impact on attendance

3. Caregivers enrolling children

Caregivers

High impact, easy to do

- Very good idea to give contact details to school
- Great idea – high impact and easy to do
- Good to give correct details and would increase children's wellbeing support (from school and home)
- Volunteers can do it or selected people from the community can support, as teachers have lots to do. Unemployed people, not those who work for other partners.
- Need to provide motivation/transport
- Teachers can do it
- Needs to be someone (paid) from the community who knows where caregivers live
- Difficult if you have many children
    - Solution suggested: older children can register younger children

Partner/district feedback

Low feasibility, high impact

- It is government policy that all caregivers accompany their children to school for enrolment at the beginning of each school year. This needs to be emphasized during implementation.
- Parents can come to school in a shift system to ease on the numbers. E.g., P1-P3 on Monday, P4-P5 on Tuesday and P.5-P.7 on Wednesday. This will also manage congestion.
- This will also help parents to know the characters of their children when at school as children behave different when at school and at home
- The volunteers to ease registration can be teaching assistants
- This will be easy to implement but have a low impact on children's attendance.
- The schools should also have other career days other than enrolment days where details of caregivers can always be updated
    - This is easy to implement and high impact

Teachers

High feasibility, high impact

- The teachers noted that this is one of the best strategy to implement
- Suggested a registration week before school opening to give the teachers enough time to do the registration, have one on one interactions with the caregivers and without being worried about conducting lessons
- This will help teachers understand the background of the chid, whether they have any form of disability, orphaned, etc
- Registration is done termly and can be organized per class

- Teachers at times fail to manage learners as they do not know their caregivers. The children misbehave because they know the teachers don't know anyone to report them too
- The teachers will also get a chance to know the behaviours of the caregivers and sensitize them about ways of supporting their children
- If a volunteer is recruited for registration, some stubborn parents might not give the correct details as compared to when the teacher is making the registration
- For some caregivers who know how to read and write, a registration form can be designed and the caregivers fill in the necessary details which can later be entered in the computer
- This will increase the interest of teachers as they have the contact details of the caregivers and can contact them in case they need to reach out to them
- Teachers will also be able to follow up on absent children as they have contacts of the caregivers
- This will improve the teacher-caregiver-child relationship
    - This would be east to implement and it would be of high impact

4. Classroom support

Caregivers

High impact, easy to do

- Like the idea (said by many), is very necessary, would be high impact
- Would help children to concentrate
- Easy to do – done in some schools already with some facilitation
- Hard to do if voluntary – volunteers have other responsibilities so would need to be paid
- Some community members are qualified by jobless – can recruit them

Partner/district feedback

- Instead of volunteers, teaching assistants can be hired as the current numbers are insufficient.
- These will greatly support the teachers with classroom management
- Even volunteers need a form of facilitation.
- There will challenges with recruitments and determining who the volunteers will be
- This will be difficult to implement due to the cost implications and sustainability but would have a very high impact

Teachers

- Assistant teachers should be the volunteers
- These should come from the community and know at least one or two of the local languages
- They will be able to help in translation for the learners who don't understand English in class and also during the parents-teacher meetings
- They will also support teachers in making learning aids and capturing attention of learners in class
- [participating organisation] is currently responsible for hiring assistant teachers, so [organisation] would need to liaise with them on how to implement this strategy

- These assistant teachers would need to be trained on basic teacher skills so that they're able to support the teachers well and participate in many school activities
- These assistant teachers can also support with registration of learners in strategy 3 above
- Assistant teachers coming from the community will reduce on the drop out of learners as they may fear them contacting their parents/ following up for missing school
- These can also be role models for these children
- They can also help with translations during parents- teacher meetings
    - This would be easy to implement but [organisation] would need to work with [participating organisation] to harmonise recruitment processes and avoid duplication
    - This would have a high impact

5. Escorting children to school

Caregivers

High impact, easy to do

- Great, high impact. One subgroup said hard to implement, the other said easy.
- Possible to do with caregivers and in groups, all options possible
- Easy and would have a large, positive impact on security
- Difficulty – language barrier between children, children could fight each other
- Caregiver can't take one child but they can go in a group
- Risk: some caregivers may pressure the child to be fast/beat the child because they have other priorities to get to, so groups is a better
- Should group children accrding to:
    - Neighbourhood
    - Language
    - Not same age, otherwise children will just play. Need some older children.
- Caregivers can sit and plan a meeting point and time and agree together
- Concern – children would fight between themselves, even if caregivers agree on the grouping

Partner/district feedback

- This will help the children with time management at school
- It will also create a culture of accountability among the pupils as they are able to understand why their friends miss school and know how to support/encourage them attend
- Children will report who will be absent with reasons when teachers are taking attendance
- Young children would need to be prepared early so that they don't make children in older classes late
- Caregivers would need to first be sensitized about this strategy as some people in the community might not want their children associating with children of neighbours (especially if the caregivers aren't on good terms)
    - This is easy to implement and would have a high impact

Teachers

- This will increase caregiver interaction with the child and improve attendance
- This will increase interest/motivation of the child to attend school
- This will increase team work and sharing of resources as children look out for each other
- Children can be grouped based on location (with children of neighbouring households)
- The caregiver also gets a chance to know other school programs as they continue interacting with the school
- The children in lower classes are able to consult children in higher classes for support with some hard questions
- The caregivers will identify challenges that the children experience on their way to school
- The children will also feel loved and cared for when the caregivers escort them to school
- The children will not be able to play between shifts
    - This would be between moderate to difficult to implement
    - This would have a high impact

6. Income-generating activity

Caregivers

High impact, easy to do

- Very good, will help all children to go to school. Can balance when and how much to contribute, e.g. Exam time
- Easy to do, hight impact
- Increase caregiver-caregiver relationship
- Mothers – great idea, very high impact and easy, for nationals and refugees
- Men - very hard and low impact because they have no land as community members, they can start and run it but draw on NGO expertise where and when needed
- One mother said there would be an issue of language

Partner/district feedback

- The youth who are out of school should also be included in this strategy
- The house holds who are most vulnerable and in need of urgent support would need to first be mapped out
- Parents who have a negative attitude towards education (don't attend school meetings are buy scholastic materials) would still not benefit from this strategy
- One of the challenges as to why children don't attend school is because of poverty, they lack beddings and the huts are extremely cold from 4am till morning. Some have leaking roofs and it becomes hard for a child that didn't have a good night sleep to wake up early for school. These are harsh conditions and there are a good number of households in this category in the community

- This initiative would require one time support in terms of resources and continuous supervision for it to have an impact.
    - This would be difficult to implement as it requires mapping out vulnerable households, sensitization and money for start-up. It would have a high impact in as far as encouraging learners in these harsh conditions attend school

Teachers

- The parents can be organized in groups and decide on what activity they want to do
- The parents will be more interested in supporting their children
- Caregivers would support their children with the school feeding
- Currently P.6 and P.7 children have porridge at lunch at school
- Parents can then decide on what issues to prioritize and address with their income
- Caregivers would need start up capital and training on the income generating activities
    - This would be moderate to implement and have a high impact

7. Translation support for parent-teacher meetings

Caregivers

High impact, easy to do if paid. If not paid, it will be difficult

- Highly supportive
- In [school name], teachers and caregivers speak different languages (Nguzu and Swahili respectively), which makes it hard for children to learn
    - Needed for caregivers to teachers and teachers to children
- High impact. Easy/difficult depends on whether they are paid.
- Can be from the community but needs to be facilitated
- In church, someone volunteers
- Volunteer can be from parents – those who know languages and in the same way as in church
- Consensus is difficult if not motivated

Partner/district feedback

- Volunteers are freely available in the community and don’t need to be hired
- These would need to be first mapped out so that they are known when they would be needed
- Some might need a token of appreciation when they translate while others might be willing to work for free
    - This would be easy to implement and be of high impact

<u>Teachers</u>

    - Volunteers should not be hired but instead use assistant teachers who should be coming from the communities and know the local languages
    - If a volunteer is to be hired, this might delay some meetings yet they might be urgent
    - Caregivers who understand both English and the local languages can also support in translation
    - Having an assistant teacher as a translator will improve the confidence of the caregivers knowing that their children understand what they're taught (no language barrier)
    - Churches usually use multiple languages as you find the pastor speaks more than 2 local languages and so the caregivers/congregation is able to understand
- This will be easy to implement and would be of high impact

8. School meeting topics

<u>Caregivers</u>

High impact, easy to do

- Good topics
- Discussion of finances – difficult to do as school may want to hide details/fraud
- Really important for the community to be involved in decision-making
- Need follow up, check implementation
- In Uganda the teacher changes a lot – need to stick with one teacher
- S4 students become teachers despite being unqualified – parents need to have more of a say in this
- Need to get agreement from the school that parents and teachers put topics on the meeting agenda

Add:

- Inspirational messages and stories
- Criteria for promoting children post-covid (children were promoted without having the appropriate level)
- Teacher dress code, especially female teachers
- Provision of refreshments at meetings

<u>Partner/district feedback</u>

- The caregivers should also be involved in the planning and priority setting on how the money will be spent; and priorities stated at the beginning should be addressed when giving accountability
- Transparency issues are fostered by caregivers who do not attend PTA meetings and spread false information

- Caregivers always think all the fees are supposed to be paid by the partners and that is not in any way their responsibility
- PTA fees vary across schools. these are usually set by the caregivers themselves. The partners [participating organisation] are in touch with OPM and UNHCR to harmonise these fees across the schools
- The PTA committee is tasked with the mandate of collecting fees from caregivers
- The SMC is responsible for implementing/spending the funds collected by PTA
- Role models for the children can be picked form the community to inspire the learners. These can be invited to school to give talks whenever the school deems it fit
    - This is easy to implement and would have a moderate to impact

Other topics to include

- How to empower schools on resource mobilization/accountability and sustainability
- Good parenting practices and the importance of education
- Inadequate numbers of teachers
- Health and hygiene of children
- Teachers' methods of teaching could have an impact on attendance. children who were in P2 learning in the local language were promoted to P4 where learning is in English. This is a challenge for children to comprehend the instructions and might affect attendance

<u>Teachers:</u>

- It is the role of the headteacher to present the budget to caregivers in the PTA meetings
- The caregivers will also get to know the accountability of how their funds are spent (remove doubt in parents)
- This will create awareness of school activities among caregivers
- Parents who miss school meetings are the ones that get frustrated with school fees payments which are usually discussed during these meetings
- Inviting role models will motivate the learners and also help them identify these important people in their communities
- This would be easy to implement and would have a high impact

Other topics to include

- How to handle and support children with special needs
- How to support children from child headed house holds

Other strategies

- Tracking children with low attendance and establishing reasons/underlying causes of poor attendance. the double shift system being implemented to addresses challenges of space in schools is having a negative impact on attendance as children who go to gardens in the morning find it a challenge to go to school in the afternoon.

Who should be involved in deciding which strategies need to be implemented?

- The PTA, SMC, Teachers. Head teacher, Partners and WCH team

How do the participants see the implementation of the strategies?

- Implementation would be gradual as every strategy can be given its own timeline

What would be WCH's role in the implementation of these strategies?

- Support financially with strategies that have a monetary implication, sensitization of caregivers,
- Organize trainings for volunteers and assistant teachers
- Supervise the overall implementation of these strategies

Participants choosing 3 strategies to implement according to school needs

	Strategy 1	Strategy 2	Strategy 3
Participant 1	8	3	1
Participant 2	2	8	1
Participant 3	4	8	1
Participant 4	1	4	8

Validation workshop: [settlement 4]

3rd March 2022

1. Attendance rewards & recognition

<u>Partner/district feedback</u>

- To increase retention, it should be surprise distribution
- There's an issue of sustainability but rewards work
- Attendance only increases around giving rewards
- There's a need for tracking attendance
- Don't give timelines or deadlines
- Scholastic materials are sold
- Some don't come for valid reasons and need these items
- Need sensitisation from teachers to children, and continuous sensitisation to caregivers
- Question from participant: are caregivers attaching value to these items?

Recognition

- Give certificates
- Could be effective
- Could give marks weekly and a monthly recognition
- Do it at the class level as well as assembly
- Create on 'leader' per group, e.g. for a class of 80 split them into 4, as this will create healthy competition and children will look out for each other
- Teachers will feel an added responsibility and need facilitation/motivation

<u>Caregivers</u>

- Giving reward will motivate children with poor attendance to attend so that they also receive rewards
- Majority of children don't attend school regularly due to the domestic work given to them by caregivers. So caregivers need to be sensitized on the importance of education for their children
- There's a challenge of sustainability as the school cannot be able to provide these rewards
- Rewarding children by reading their names in class and assembly would motivate the children but not to a great extent as material rewards
    - This would be easy to implement if proper planning is made and the rewards are provided to the school to implement
    - This would have a high impact on attendance

<u>Teachers</u>

- Good but difficult to afford
- Termly is better
- 343 children in P3 – 100 are absent per day – a lot to track, so weekly is better

- Very few have sanitary products so would really reduce absenteeism of girls
- Would also increase discipline

Recognition:

- The refugees want something tangible
- Learners do not have scholastic materials
- Just a thank you wont have affect
- Verbal recognition will cause a possible 10% increase
- Reward for positions 1 to 5

2. Homework support groups

Partner/district feedback

- Good idea, brings learners together and had other benefits, e.g. social and teamwork
- Improves caregiver-child relationship and feedback to the school
- Will improve attendance and performance
- Good to put different abilities together to support each other
- Time efficient
- Challenge during school time so suggestion is on Saturdays/at the weekend, although caregivers are busy at the weekend
- Would generate a change of mindset – creating time for the child, if the attitude is positive
- Added outcome – social cohesion, plus between caregiver and child
- The caregiver would be able to translate
- Move responsibility into the home
- One lead caregiver could drive the group but it can be rotated within the group to create a shared responsibility
- Needs to be well-planned

Caregivers

- This will create friendships among children and enable them complete homework. This will in turn improve performance
- There's a gap between the refugees and host communities in [school name]. So, parents might not want their children grouped in classes and neighbouring households but rather as refugees and hosts
- Caregivers will need to be first sensitized about the importance of letting their children work in groups/supporting each other regardless of whether they are hosts or refugees.
- Village education committees can be helpful in sensitizing the caregivers; though they will request for a token of appreciation
- The children should not be more than 10 in a group if they're to benefit from each other
    - This will be difficult to implement as caregivers are not sensitized and there are segregations in communities by nationalities

- This would have a high impact if implemented

Teachers

- Could be implemented every evening and would increase performance
- Parents and caregivers usually add on homework
- Would especially reduce dropout
- Good idea – using the family language will increase understanding
- Would be more effective if combined with sensitisation of caregivers on importance of homework
  - Could be 1-to-1 or with groups
  - They will need facilitation otherwise they will not come
  - Need a change of mindset amongst caregivers
  - Caregivers can be guided by teachers; teachers can monitor and caregivers can report back to administration
  - Would increase the motivation of children
- Daily homework, so do daily groups
- Should rotate between different caregivers, the children will want to impress them
- Role of the school would be to mobilise the community for implementation
- It would make additional work for the school

3. Caregivers enrolling children

Partner/district feedback

High impact, mixed review of feasibility

- This should be a must, as it is the responsibility of the caregiver to accompany the child to school – gives a sense of responsibility
- Great idea, also for child protection
- Names of the child need to be updated so that the family can be traced
- P7 – important to have correct names for access to services
- Need sensitisation, also to fathers – the engagement will differ between the two
- Enables teachers to follow up with caregivers for poor attendance
- Will increase enrolment within the correct class and correct school
- Will be difficult to do (disagreement within group – some feel it will be easy) – will require a huge group effort, e.g. OPM, schools, partners etc
- School leaders should be involved
- Change of mindset is the most important
- Law enforcement plays a role

<u>Caregivers</u>

- Caregivers can accompany their children to school and this is very easy to do
- The volunteers can be hired from the community and paid a token of appreciation/facilitation at the end of the day
- Teachers would also volunteer to register the children but would require computers where this information is captured as the school doesn't have any computers
- Through parents' general meetings, parents agreed that children from P.4 to P.7 attend classes on Saturdays and teachers would be paid with remedial. This was agreed upon to improve performance of learners
- Through this meeting also, it was agreed that P.1 -P.2 pupils who usually left at lunch time leave at 4:00pm
- Then P.3 – P.7 pupils leave school at 5:00pm
- Parents also agreed to pay 1000 shillings for every set of exams their children do and agreed on 4 sets of exams for the children per term. This is geared towards improving the performance of learners and the school in general
    - This is easy to do and would be of high impact

<u>Teachers</u>

High impact, low feasibility if meant to be done by teachers during school holidays

- Good, giving teachers skills will help to get accurate information from caregivers. Would breach the gap between teacher and learners, and address issues of discipline and disability
    - Would help caregivers and teachers identify areas of weakness and difficulty and come up with possible solutions
- Would help teacher to get to know caregivers and exact caregiver of each child, which is important from when there are accidents or issues
- Creates relationship between caregiver and teacher, especially for when dealing with children of different nationalities and cultures to their own
- Help with children with disabilities, e.g. can make sure that a child with hearing difficulties can sit at the front
- Caregivers don't want their children to be mixed with others
- Needs sensitisation in advance, cannot be the teachers as they go home during the school holidays
- Registration is the beginning of each term, needs to be one week before. Registration is all done in one day.
- Excel skills would increase sustainability - training aspect is important.
- HT: 50-50 on impact; will definitely reduce the communication gap between teachers and caregivers but the biggest cause of absenteeism is home factors and not the child's decision. Need increased follow up and increased discipline

4. Classroom assistants

Partner/district feedback

- Good idea but needs good planning
- Needs to have clear roles, responsibilities and communication
- Assistant teachers' role could be behaviour management and translators
- Should be hired from community
- Paid positions but could have incentives; UGX 250,000 per month (~64 EUR)

Caregivers

- This strategy is great as the PTA and SMC have implemented it with the assistant teachers who support learners and keep them attentive. The children actually concentrate more rather than playing like they do at home
- Children get motivated to attend school as they feel listened and attended too.
- Children will be able to see the difference between home and classroom environments hence pay more attention
- Teachers would get to know the children's characters more and how best they can support them
- Teachers can also easily identify children who don't attend regularly
- The volunteers can be either a trusted caregiver or community member but they would require an incentive as a motivator
- Some respondents said the volunteers can come from outside the community so that the community/children respect them. This would also reduce personal conflicts between caregivers and teachers
    - This would be difficult to implement as it requires conducting interviews/recruitment for these people, payment of their salaries and organizing for them accommodation facilities near schools. Funds would be needed for this to be implemented as the school cannot manage its implementation.
    - This would have a very high impact on attendance

Teachers

- Good idea.
- Children have short attention span (30 mins)
- Volunteers can be trained teachers from the community
- Can't rely on volunteers. HT is involved in programme with [organisation], where they hire graduates and school leavers. Only 2 out of 10 are trained. They don't want to mark books, come late and don't support – not a good experience.
- Available from community. Can be paid UGX 300,000 pcm
- Need volunteer who knows local language

- Would reduce caregivers' negative attitudes towards education quality

5. Escorting children to school

Partner/district feedback

- Older children escorting children is a better idea
- Would give sense of motivation to younger children
- It's more sustainable to group children, and is safer
- Increase concentration on children from caregivers
- Neighbouring households should go together as they go to the same school
- Sustainability will be a challenge
- Low impact
- Different classes go at different times
- Easier if go according to age (but this clashes with above point – respondent could have meant class)
- Need to have caregivers involved
- Neighbours may have issues between them as there is a lot of diversity within the settlement and language/cultural issues

Caregivers

- This is a good strategy as children who get lonely on the way to school feel safe and reach school
- Parents are also assured that children have reached school and will return safely
- Parents can also ensure that once in a while, they pick their children from school and this will affirm to them that their children are indeed in school
- Parents who fear older children or children of their neighbours escorting their children to school should do it themselves
- This will motivate parents and children to always get ready early for school so that their friends don't leave them behind
- There's a lot of commitment needed from the caregivers especially those that have bad attitude towards education. They need to be sensitized on the importance of this strategy for it to work well
- Caregivers can agree to meet and decide on how to group their children, places where these children can always meet and time for them to set off for school
    - This would be difficult to implement due to the huge division between host and refugee communities. Caregivers might not want their children grouped together or might want groups by nationalities which might not be possible
    - This would have a high impact on attendance

Teachers

- Good but escorting by caregivers is better
- Grouping could work – children could tell teacher which children don't come

- Grouping is better, with a group leader
- Group by village. Establish a meeting point and time. Need to be at school at 7.45am
- High impact – children like being in groups
- Difficulty would be children coming from different locations – some might be left behind
- Easy because it starts with children and reduces caregiver burden

6. Income-generating activity

Partner/district feedback

- Refugees sell maize before even planting it
- If this is linked to activities already being implemented, it's possible, however if it's an innovation it will be difficult
- NRC – gives start-up funds but not yet in Isingiro to a group
- Could do this through partnerships
- Should group caregivers based on their interests and skills – need to be supported
- Risk: increased domestic violence from men wanting to buy alcohol. Still need to involve mothers and fathers though.
- The group should give money directly to school, so as to avoid the above and to impact the intended outcomes
- Paying for fees themselves will increase follow up as they will have invested in their children's education
- Needs to be contextualised
- Dependent on mindset

Caregivers

- Caregivers who can afford scholastic materials for their children still refuse to pay children's fees or cater for their needs as they think this is the role of government, OPM, UNHCR or partners
- Some respondents said income generating activities can be set up for teachers instead to enhance their salaries and motivate them to teach their children
- Some caregivers believe every parent should find a way of supporting their children through school
    - This would be difficult to do as it requires a lot of funds to set up
    - This would have a low impact on attendance as some parent's attitudes towards education are the problem and not lack of money.

Teachers

- Good but need sensitisation first on advantages of group activity
- Good but not sure if beneficial to caregivers, better to do with learners, and caregivers would supervise (- child labour concern)
- Sensitise parents on saving and financial management. Would need follow up and advantages
- Use learners to inform parents

7. Translators for parent-teacher meetings

Partner/district feedback

- Can be volunteers
- Issue with the diversity in the settlement – many different nationalities and languages
- Assistant teachers could play this role

Caregivers

- Translators are many in the communities but always want to be paid for their services or given a token of appreciation after translation
- Volunteers are not paid a salary but a token of appreciation
- An example was given that there are usually 4 translators per meeting, translating into Runyankore, Kinyarwanda, Swahili and Somali
- In churches, there are no issues of language barriers as they gather based on languages they speak; so nationals alone and refugees alone
    - This would be moderate to implement as the translators always want to be paid
    - This would have a high impact

Teachers

- Good if they are hired
- Need to translate for multiple languages
- Would increase action from caregivers
- Teaching is in English – translation is needed in the classroom too
- Churches are made for one language – done in shifts.
- Interpreters are selected from community leaders and are paid in village meeting
- Would increase communication as teachers are from elsewhere
- Volunteers always need to be paid

8. Agenda topics for parent-teacher meetings

Partner/district feedback

- Good idea – discussions are needed, and need parents' input
- Parents should agree on payment and accountability of school
- Involvement of parents in need of school – would be more supportive

Additional topics:

- Child protection, gender, labour, child safeguarding, relationship with host community, early marriage

Implementation model:

- Should be gradual. Needs to be decided by the community and the school, and raised at PTA meeting and SMC.
- Need to bring this to a general meeting with parents

Priority strategies per actor

[agency 1]	[participating organisation 1]	[agency 2]	[participating organisation 2]	[participating organisation 3]
6	7, 8, 1 (sustained with 6)	6, 1	1	8, 6, 7

<u>Caregivers</u>

- These topics are good at informing caregivers about school developments
- Role models would inspire the children and caregivers to focus on studies and achieve their goals, but these should be selected from the community
- Parents who miss these meetings are usually the ones that spread wrong information in the community. Also due to language barrier during meetings, some parents take wrong information to those that miss the meetings
- Volunteers who can help translate during these meetings should at least speak 2 local languages that the caregivers understand to ease communication
- Some teachers who usually fail to update attendance registers discourage the PTA & SMC chairpersons from checking these registers. They speak to them rudely to bar them from supervising their work
- The PTA & SMC committees are essential in sensitizing communities as they were essential in the last lockdown when distributing home learning materials to learners
- The school set up beginning of term exams for children so that they all report within the first week of school
    - This would be very easy to implement and would have a high impact

Other strategies

- Utilize local leaders in the communities to sensitize caregivers about the importance of education
- Ask children who continuously miss school to bring their parents and meet the headteacher/teachers for discussions
- In PTA meetings, sensitize caregivers on how to support children with disabilities
- Other topics to discuss in these meetings include teenage pregnancies, family hygiene, nutrition and balanced diet for children, opportunities for caregivers to volunteer/support at school

Rating of strategies in terms of implementation (There were disagreements when it came to what strategy would be rated what number, so each caregiver rated the strategies independently)

**Strategy rank**	**caregiver 1**	**caregiver 2**	**caregiver 3**	**caregiver 4**	**caregiver 5**	**caregiver 6**	**caregiver 7**
1	2	2	1	2	1	1	1
2	4	7	7	3	5	2	4
3	3	1	2	1	2	6	2
4	1	3	3	4	4	3	3
5	7	6	5	7	3	8	8
6	8	8	6	8	7	4	7
7	5	5	8	6	8	7	6
8	6	4	4	5	6	5	5

Teachers

- Will be high impact if all stakeholders are involved, incl. parent leaders, implementing partners
- Will be difficult to do though because it takes time to conclude discussions
- Will be easy (said by many)
- The partners will generate more impact

Exam fees

- School fees is an important topic – caregivers go to the OPM and complain about them
    - Once negotiated, e.g. paying in instalments, caregivers agree
- Parents call the meetings.
- Costs can seem very high when there are many children in the family
- PTA and SMC decide school fees then call a general meeting with caregivers and vote on what fees are spent on.
- What about unaccompanied learners? About 100+ are unaccompanied at the school.

Supporting learning at home

- Yes, advise them for slow learners

Role models

- Most learners' ambitions are just to finish primary schools – tell caregivers that there are other levels of education
- Role models should be community members
- Change mindset – some have bachelors degrees
- Role models should come and speak to learners – more important than to caregivers, as they need aspirations and motivation

- Need to change the mentality from being refugees, getting married and having children. They often get married straight after P7, some earlier.

Topics to add

- Feedback on children's academic performance – though there's the issue of confidentiality
- Encourage parents to come to school and ask about their children's academic performance – they should also be holding the teacher accountable
- Personal hygiene

Additional ideas:

- School feeding programme
- Water source and water capture

Overall comment– difficult, partners keep adding more responsibilities to teachers
