# Supplementary material for: Increasing school attendance and retention in refugee settings: Participatory development of the SchoolLinks intervention
Source: PLoS One. 2026 Aug 31;21(8):e0357215. doi: 10.1371/journal.pone.0357215 (PMC13528952; doi:10.1371/journal.pone.0357215)
Supplement: Supplementary file 6 [file pone.0357215.s006.pdf]

Compiled prototyping field notes

Contents

Strategy 1: Meeting with Headteacher and Deputy Headteacher 2
Strategy 1: Meeting with teachers 4
Strategy 1: Meeting with community leaders 6
Strategy 2: Meeting with translators 9
Strategy 1 & 2: Meeting 1 - P3 stream A 10
Strategy 1 & 2: Meeting 1 - P3 stream B 13
Strategy 1 & 2: Meeting 2 - P3 stream A 17
Strategy 1 & 2: Meeting 2 - P3 stream B and AEP1 19
Strategy 3: Enrolment week 21
Strategy 4: Attendance monitoring (week 1) 23
Strategy 4: Rewards celebration 24
Strategy 5: Homework clubs 26
Strategies 5 & 6: Homework clubs and Safety to School 27

<u>Strategy 1: Meeting with Headteacher and Deputy Headteacher</u>

Activity: Inception meeting with the Head Teacher and Deputy Head Teacher held on the 12/7/2022.

Venue: [location]

Participants

No	Name	Position
1	[name]	Research coordinator
2	[name]	Research officer
3	[name]	Headteacher
4	[name]	Deputy headteacher

Points discussed: below were the points discussed by the Research team

- Introductory remarks from the organization
- Status update of the research from inception up to current status
- Next steps for the research where details of the pilot study were shared.
- Explanation of what a pilot study is and why it was being done with only P.3 class
- Sharing the details of the different strategies below and how each would be piloted with P.3 class

The strategies included:

1. Entry meetings
2. Translation
3. Enrolment
4. Attendance recognition and reward
5. Homework support groups
6. Accompaniment

Reactions from the head teacher and deputy head teacher

After a thorough explanation of the various strategies that make up the caregiver engagement intervention, below are the reactions from the discussion

- On the venue for the meeting with the teachers, the school administrators advised that we could have the meeting from the staff room on an even they decided since classes end at 4:00pm and teachers would grant us 30min of their time to have a meeting with them
- On the venue for the meeting with the caregivers, the school administrators advised that we could have the meeting from one of the class rooms since most of the caregivers have more than one child in the class and the number may not be too big. They said the class room will accommodate all.
- When the administrators were asked on how these caregivers would be invited, they said they send the children to communicate to their parents/guardians verbally, telling them to school for a meeting. This information is normally passed on earlier and the teachers keep reminding the children throughout the week until the day of the meeting.
- It was then agreed basing on the advice from the head teacher that, invitation letters would be sent to the caregivers through the children, inviting them for the meeting. The headteacher drafted the invitation which was later shared with the children to take to the parents.
- It was left to the discretion of the P.3 teachers and class teachers to determine which stream would participate first.
- When asked on how many teachers teach in Primary three, the school administration said are four (4).

- On translation, the head teacher said they have been supported by assistant teachers when they have meetings at school with caregivers. However, she noted that there have been challenges with these assistant teachers because they do not comprehend English very well, so they end up adding their own words to what the speaker says, or end up distorting the meaning of what the speaker intended to say by adding their own words.
- She then advised that we could get a translator from the community to work with one of the staff members who is good at translation and very fluent in both English and Bari.
- When asked on how translations are done in places of worship in the community, it was noted that there are a number of churches and people attend based on the language of the church leader or language spoken in church.
- After establishing that there are two major languages spoken by majority of people in the community, it was agreed to have two translators one for Bari and the other for Juba Arabic.
- It was also noted that the glass noticeboards proposed to be used for strategy 4 would be broken by the pupils in matter of hours as they are extremely rough. It was advised that we get portable notice boards probably made out of soft wood. These can be moved to class for children to see and after taken back to the storage area.

Next steps to be taken

- Coordinate the translators for an inception/preparatory meeting the next day
- To organize an inception meeting with teachers the following day
- To collect the primary 3 enrolment list
- To draft invitation letters for the caregivers
- Coordinate with P.3 class teacher and DOS to choose the stream to for the meeting.

Strategy 1: Meeting with teachers

Activity: Meeting with the Teachers held on 13/7/2022

Venue: [school name]

Introduction: introduction session by all members present

Opening remarks: from the head teacher

- The opening remark by the school head teacher was brief. She welcomed everyone for the meeting and again appreciated [ORGANISATION] for their tireless efforts in supporting [SCHOOL NAME] and especially fixing the solar system that had been spoilt. She also thanked the staff members for turning up for the meeting in large numbers.

Remarks by the RC:

- These included a status update of the research from inception up to current phase.
- Explanation of what a pilot study is and why P.3 would be the class participating in this pilot
- Presentation of the strategies which included the following
  1. Entry meeting
  2. Translation
  3. Enrolment
  4. Attendance recognition and reward
  5. Homework support groups
  6. Accompaniment to school
- The phased implementation model was also shared in detail for the staff members to understand.

Reactions from the teachers

- One of the participants wanted to know whether [ORGANISATION] was providing new registers for this particular activity and if these registers would be different from what they currently use. This was ably answered by the RC as the teachers would use the existing registers to continue monitoring children's attendance.
- The teachers also shared the below information which they would like to be captured on the enrolment register.
  - Date of registration
  - Name of child
  - Date of birth/Age
  - Individual number (caregiver)
  - Individual number (child)
  - Household number
  - Old pupil/Transfer in (If transfer in, from where?)
  - Name of male caregiver
  - Name of female caregiver
  - Parental status
  - Special needs/Health condition eg hearing impairment, sight impairment etc
  - Location data eg, District, County, sub-country, parish, zone/block

- One participant inquired whether it would be possible to capture the photographs of the learners during registration. The RC noted that this would not be possible as it would slow down the registration process

including the tablets. The pictures of these children are included on their attestation cards and this information is available at OPM incase the schools need it.

- One of the participants who participated in the GMBs and the Validation workshop was excited to hear the various strategies and appreciated [ORGANISATION] for piloting the exact things that were discussed in all those meetings.
- Majority of the participants pledged their support for the pilot study and requested that the strategies also be implemented in the whole school after learnings from the pilot.
- One participant also wondered whether the teachers would also be rewarded during implementation of strategy 4. The RC noted that they were not going to be rewarded.

Closing remarks by the Headteacher

- Appreciation remarks to [ORGANISATION] for their endless support in the learning of children and to [SCHOOL NAME] specifically.
- She thanked [ORGANISATION] for choosing [SCHOOL NAME]to be the pilot school and pledged her support for the study.
- She again thanked [ORGANISATION] for repairing the Solar system that had broken down and was positive that children would again resume learning on the tablets.
- She encouraged the teachers to support these programs so that the school and the entire community can benefit from them.

Strategy 1: Meeting with community leaders

Activity: 1st Entry meeting with community leaders

Venue: [CHURCH NAME]

Date: 12/07/2022

Introduction

- Introductions were made by the research team followed by the community leaders. Everyone got to know the other before the star if the meeting
- The research team assessed the commonest language majority of the participants spoke/understood and an agreement was reached that the meeting would be done in Kakwa since all the leaders could understand it.
- Introductory remark were given by the presenter highlighting why it is important to involve the community leaders in the caregiver engagement intervention for the learning of the out of school children in the communities.
- A brief background on the development of SchoolLinks was given and then the strategies below presented with the help of visuals that were already hanged in the room.

1- Entry meeting between the school and care givers
2- Translations
3- Enrolment

Participants were then asked why it's important for caregivers to enroll their children

- Taking children to school for enrolment by parents is important because one will be able to give the school the correct name of the child. One participant gave an example that there was a program by one of the organizations giving assistance in the settlement, since her daughter did not register with the correct name that is on the attestation card, she missed that opportunity of receiving the assistance that was being given. She therefore encourages enrolment of children by parents to be taken seriously.
- It is important to take the child for enrolment because the caregiver can be able to give the correct age and class of the child to the teachers so that this child can be known by the school rightful and attends the right class.
- Taking a child for enrolment will make the parent to clearly explain to the teachers the challenges of this child. For instance, if the child has any type of disabilities, this will be explained to the teachers for child can get the necessary help he/she needs.
- Taking the child for enrolment will make this child feel that his/her parents are interested in his/her education and this will give the child morale to concentrate on his/her studies.
- This will create unity among the caregivers and the school since the teachers will be able to know the parents as they come with their children for enrolment.

Presentation of the strategies continued.

4- Attendance recognition and reward

5- Homework support groups

6-Accompaniment to school

Discussion by the leaders on the presentations

- It was noted that it is important to educate a child because once a child is educated, this brings light to the whole family. The family will be respected by other community members.
- This educated child can help in community wellbeing for instances in issues to do with sensitization on community hygiene, sanitation and general health.
- When the child is educated, he/she can help take up some of the family responsibilities such helping siblings with homework, paying fees if he/she has a job, encouraging the rest of the children to study hard since he/she would have known the importance of education.
- One of the participants noted that the homework support group strategy should always be emphasized in the parents meeting so that caregivers can fully participate in the program. They all pledged to support the children by supervising the HSGs.
- Participants also noted that the homework support groups will help to bring unity among the different families since caregivers will be looking at these children as one family. They pledged to encourage parents with OOSC to bring them for enrolment so that they benefit from these wonderful engagements.
- The HSGs will make the weak children learn from the bright ones since they will be freely sharing among themselves.
- Participants also appreciated strategy 6 and noted that if children move as a group and some are not in school uniform, those not in uniform will force their parents buy for them uniforms so that they all look smart.
- Moving to school as a group by the children will help reduce/prevent accidents amongst the children. The children should be told to hold their hands while crossing the road so that speedy cars or motorcycles can stop or reduce speed for them to cross.
- Some caregivers in the community are naturally hostile and don't like children coming to their homes. Such caregivers won't allow homework support groups in their homes. Sensitization will be done so that they understand the importance of children learning from each other. The presenter explained that caregivers will be requested to voluntarily offer their homes for this strategy and caregivers who refuse their children from participating will be not be penalized though it will be a disservice to the children.

Questions from the participants

1. Participants asked what age and level of drop out (class the child dropped out) would be considered while selecting these out of school children?

- The participants were informed that all children of school going age who are currently out of school would be involved in the study as we would like to link them back to school.

2. A participant was also concerned as to why [ORGANISATION] waited from beginning of term one and was now bringing such a wonderful program in 3rd term?

- Participants were informed that the grant which was secured for this program happened in March when Term one had already started. Then the research team within the organization underwent preparatory phases to ensure things would run smoothly including seeking ethical approvals with the different government bodies; all which take time. So, implementation couldn't begin until the necessary approvals were granted. These occurred in July and August hence commencing the study immediately.

3. One of the participants shared a painful experience they're going through with one of his children who has refused to attend school. That this child adamantly refused to attend school without any reason, stole the mother's money and ran away to the uncle's place in [LOCATION]. Upon reaching there, she was heard saying that she could not continue with education because she feels she is too big for the class she was currently in and is no longer interested in primary.

- The caregiver was advised to convince this girl to come for the workshop that would be happening on 16th [LOCATION] so that she could be talked to understand any other issues that could be surrounding her refusal for education. The caregiver promised to encourage the child to come to the workshop as he expressed that the discussion had not only torched his heart but also pierced his liver and would love to see his child back in school.

4. Another participant also shared another experience that was aching him. He said the daughter failed P7 and he advised her to repeat the class but she told him that the he knows very well that she is very dull (weak) to pass P7, that if he wanted her to go and repeat the class, he should buy for her a new brain to be able to pass and if he cannot do it, let him leave her alone. That the girl disappeared from home and he hears she is in Kampala. He therefore urged the leaders to work hand in hand with organizations that support these kinds of children to see that they return to school children.

Mobilization for the future strategies:

- Participants were requested to identify out of from each block that would participate in the GMB workshop on 16th August. Caregiver consent forms for these children were also shared so that the caregivers of these children would consent for their participation.
- Enrolment week: information that would be collected during this week was shared and the leaders were requested to mobilize all caregivers who currently have children out of school to bring them to school for enrolment. The caregivers should be told to bring children's ID documents (incl. registration card, birth certificate) to ensure consistent name across official documents.
- Recognition and rewards: The rules of the star chart were clearly explained to the participants and informed that the children would also be informed accordingly.
- Homework support groups: The number of groups that would be formed were clearly explained and caregiver involvement in hosting these groups and supervising shared. Caregivers were also asked to voluntarily suggest their names and contacts if they would be willing to host these groups when time for this strategy starts
- School companions: Caregivers will be encouraged to support their children by waking them up and preparing them early so that they leave with the rest of the group when the time for that strategy comes. Caregivers will also be sensitized on the importance of escorting their children to school to show love and support to their children.

Strategy 2: Meeting with translators

Activity: Meeting with translators held on 13/7/2022

Venue: [location]

Participants:

No	Name	Title
1	[NAME]	Research coordinator
2	[NAME]	Research officer
3	[NAME]	Translator 1
4	[NAME]	Translator 2

Points discussed

- Introductory remarks from the organization/ SchoolLinks research
- Status update of the research from inception up to current status
- Next steps for the research where details of the pilot study were shared.
- Explanation of what a pilot study is and why it was being done with only P.3 class
- Sharing the details of the different strategies below and how each would be piloted with P.3 class

The strategies included:

7. Entry meetings
8. Translation
9. Enrolment
10. Attendance recognition and reward
11. Homework support groups
12. Accompaniment

Reactions

- Appreciation of the efforts put by [ORGANISATION] in helping the learners in the field of education.
- The translators pledged to translate during the meetings and felt honored to be doing so.
- One translator noted that the community does not take educational matters seriously and he pledged to continue with mobilization of caregivers for these interventions.
- One noted that the community is made of many people speaking different languages. They include Kuku, Bari, Pojulu, kakwa, and the rest including juba Arabic but all having Bari as the main language.
- It was then agreed that the languages of translation would be Bari and Juba Arabic and the 2 translators each chose the language they were to translate in.
- Bari was to be translated by [Translator 2] and Juba Arabic by [Translator 1].
- The translators were cautioned to speak slowly and clearly when translating
- They were also advised to keep as much as possible to the original wording by the speaker.
- They were also advised not to engage in discussions with participants during translation but rather when an opportunity for Q&A arose.
- They were also advised to keep time for the meeting on Saturday 16th at 9:00am

<u>Strategy 1 & 2: Meeting 1 - P3 stream A</u>

Activity: 1st Entry Meeting with the Parents of Primary 3 stream A

Venue: [SCHOOL NAME]

Date: 16/07/2022

Introductions

- Introductions were made by the research team, translators and teachers present & appreciation of the translators and caregivers for honoring our invitation
- Group agreements regarding translation were made that the languages to be translated into included Bari, Juba Arabic and Aringa
- The presenter highlighted the importance of education and necessity for caregivers' engagement in the learning of their children
- A brief background on the development of SchoolLinks was given and then the strategies below presented with the help of visuals that were already hanged in the class room.

1. Entry meetings between caregivers and School
2. Translation
3. Enrolment

- After presentation of these strategies, the caregivers were given a chance to ask any questions and also check their understanding of the strategies. The questions and reactions included the following.

Questions from participants

1. Are the parents that participated in the GMB workshops the ones that have been invited for the current meeting?

- All the above questions were ably responded too by the research coordinator to the satisfaction of the participants.
- Caregivers were then asked why it is important to bring their children for enrolment and below are some of the reasons they gave.
    1. It makes the caregiver know whether the child is registered in the right class. Some children may fail to be promoted to the next class and if such children come to register alone, they may end up promoting themselves to the next class. But if you bring this child yourself, you will register him in the right class.
    2. It enables the caregiver share the child's challenges/ health conditions if any with the teachers so that this child is helped. Say, if your child has a hearing impairment and you have informed the teacher about it, this child will be helped to sit in front.
    3. It makes the caregiver show love to the child and the child will feel loved by the parent
    4. To join hands with the teachers and the school administrators and show support and love for education of our children

- Presentation of the following strategies continued

4: Attendance recognition and reward

5: Homework support groups

6: Accompaniment

Discussion by the caregivers on the presentation

Below are the remarks and questions from the caregivers

- When it is time to return to South Sudan, we will return with children who are well educated and are able to take up leadership positions of our country, thank you [ORGANISATION] and the partners working together to educate our children.
- As a caregiver, having many uneducated children in a household is painful and so we would love to have all our children educated so as to have a better future
- Parent should wake their children up by 6am and prepare for school because you find some children sleeping up to 8am and not interested in going to school. Parents should monitor them to go to school by 7 as it's a parent's responsibility.
- Even the white people support their kids with homework, we should do the same as caregivers since we have been given that information now. But it's just that parents here think this is not our responsibility. Thank you for this information and we are happy and will be able to support these children when it's time for this strategy.
- Caregivers were very appreciative of the presentation and engagement and thanked [ORGANISATION] for spearheading educational programs for their children. "Thank you so much for this kind of teaching for caregivers, never seen before or been involved in any of its kind"
- It was noted that education is key in the lives of the children because without it, the children will not have any good future. Parents were urged to cooperate and support such kind of programs/ interventions especially the Homework support groups and enrolment
- Happy about strategy 3 and its importance, we urge parents to come and register their children when called upon

Questions:

- There are few tablets in the school and not all children are able to study on tablet. Caregivers requested for the number of tablets to be increased and children in other classes included.
- One caregiver noted that the reason as to why children don't attend regularly is because of dirty uniforms and parents cant afford washing soap. How would caregivers be supported with this challenge?
- One caregiver encouraged the rest to bring children to school since the issue of translation has been handled and they're able to communicate freely in this meeting. When they come to school, they will be able to talk to the teachers of these children since someone will be there to translate for them.
- One caregiver was heard whispering to colleagues that for once, she has attended a meeting where she has heard what they're communicating and the presenters are also able to hear her views.
- One caregiver asked how the children would be grouped for the homework support groups as they live far apart or in different blocks; and this was ably responded too by the RC.
- one caregiver noted that the homework support group were a good initiative because it would bring the children together creating friendships amongst them and making the weak children to learn from those fast learners and the bright ones.
- There was also a concern by some parents who see some children playing on the playground while others in class (during class hours). This was ably responded too by the acting head teacher who was in the meeting.
- No of learners being low on tablets and tablets being few, will the tablets be increased?
- Who will supervise the children during the homework support? This was ably answered by the RC.

Feedback on the Visuals

- One caregiver noted that the children needed to appear in school uniform (same color), dresses for girls and shorts and shirts for boys in strategy 3,4 and 6
- Also, the children characters need to be decently dressed and not in mini dresses or skirts as seen in the pictures

Mobilization for the future strategies:

1. Enrolment week: information that would be collected was shared and caregivers were asked to bring children's ID documents (incl. registration card, birth certificate) to ensure consistent name across official documents.

2. Recognition and rewards: The rules of the star chart were clearly explained to the caregivers

3. Homework support groups: The number of groups that would be formed were clearly explained and caregiver involvement in hosting these groups and supervising shared. Caregivers were also asked to voluntarily suggest their names and contacts if they would be willing to host these groups when time for this strategy starts

4. School companions: Caregivers were asked to support their children by waking them up and preparing them early so that they leave with the rest of the group when the time for that strategy come. Caregivers were also encouraged to escort their children to school to show love and support to their children.

5. The caregivers were informed of the next dialogue meeting which would be during the first month of 3rd term. Their children would inform them of the meeting and the venue would still remain the same.

6. Time management for next meeting was also emphasized

Challenges:

- Late coming of participants which delayed the start of the meeting

<u>Strategy 1 & 2: Meeting 1 - P3 stream B</u>

Activity: 1st Entry Meeting with the Parents of Primary 3 stream B

Venue: [SCHOOL NAME]

Date: 23/07/2022

Introductions

- Introductions were made by the research team, translators and teachers present & appreciation of the translators and caregivers for honoring our invitation
- Group agreements regarding translation were made that the languages to be translated into included Bari, Juba Arabic and Aringa (one caregiver spoke Aringa and was translated too one on one)
- The presenter highlighted the importance of education and necessity for caregivers' engagement in the learning of their children
- A brief background on the development of SchoolLinks was given and then the strategies below presented with the help of visuals that were already hanged in the class room.

4. Entry meetings between caregivers and School
5. Translation
6. Enrolment

- After presentation of these strategies, the caregivers were given a chance to mention why they think it is important to take their children for enrolment themselves and below were their responses:

Responses from participants

1. The caregivers were very thankful to [ORGANISATION] for inviting them for the days meetings and teaching them why its important to be involved in the learning of their children; and the various ways that they have been taught.
2. To explain to the teachers the exact names of the child because you may find that this child is named by the grandparents and the child chooses to use other names other than the family name.
3. To capture real name of the child instead of a nickname e.g., boy, girl that they call themselves
4. To tell the teachers the real age of the child
5. To tell the teacher if the child has a health condition that may affect his or her learning e.g. hearing or sight impairment.
6. Some children don't know the class they belong too, when they are sent to school alone, they decide to hang around their friends and attend classes their friends attend yet they might be the wrong class. A child can easily attend a wrong class for over a month if the caregiver doesn't come to school to register her/him in the correct class. Also some children who might have been advised to repeat classes might refuse and go to next class if the caregiver doesn't come and check that the child is in the right class.
7. Some children share similar names. For example, 2 children in a similar home can have 2 similar names. So a caregiver has to come to school and help the teacher know that these are 2 different children and specify what other initials can be added to differentiate them. Even many children in the community share similar names, so these too can be differentiated when the caregiver helps the teachers.
8. Sometimes caregivers give these children money to pay for PTA and these children eat it. So, if the caregiver brings the child for enrolment, they will know if the child paid the PTA contribution and also know the correct amount to be paid. Also, these children are denied scholastic materials when they don't pay these PTA fees yet the caregiver would keep wondering why their child wasn't given books and pens yet they gave them the money to pay.

9. To tell the behavior of the children at school if it's different from how they behave at home. Some children are usually humble at home and very stubborn at school giving the teachers and their colleagues hard time. But when a caregiver comes to school, they can be informed of their child's behavior and disciplinary measures taken appropriately if any are needed.
10. When caregivers bring their children for enrolment, it builds parent teacher relationships as the two are free to communicate with together for the benefit of the child.
11. When a caregiver accompanies the child to school for enrolment, he/she can deliver the right information about the child to the teachers.
12. When the caregiver accompanies the child, it builds the parent child relationship as the child will feel more loved and cared for. They will know that their caregiver values their education and also put more effort in class
13. It will enable the caregiver to know the exact PTA fees to be paid if the caregiver missed attending the meetings where these fees were being discussed.
14. To track the child's performance and know how they are doing over time.
15. To safeguard these children on their way to school as some children face different challenges on their way to school

- **Presentation of the following strategies continued**

4: Attendance recognition and reward

5: Homework support groups

6: Accompaniment

Discussion by the caregivers on the presentation

Below are the remarks and questions from the caregivers

- Parents should at the beginning of the term their children to school for enrolment instead of sending them with Attestation cards. No child will be registered without the parent. This was emphasized throughout the discussion and parents agreed to
- One caregiver was extremely happy about strategy 6 and noted that children who fear crossing busy roads would be helped by their peers. Also children who don't know how to cross the road or those who carelessly cross the road will be guided by their peers. This is comforting to us as parents knowing that our children will reach school safely and return safely, thank you so much for this strategy as it will reduce on the number of road accidents for the children.
- One of the teachers emphasized the importance of caregivers bringing their children for enrolment and requested that the enrolment program should not only target P3 alone but rather be rolled out to the whole school. This is because they have persistently experienced challenges with children's names and as school are getting a lot of headaches from parents regarding the differences in names. He cited an example of a girl in P7 who wanted to be registered as [name] but the parents wanted her to register as [different name] and that caused a lot of rifts between the school and the parents of the girl since these could not come to the office together to agree.
- The teachers also noted that they previously faced a challenge with one child mother who had disappeared from school and her whereabouts are not known up to now because she was using many different names; and they also don't know who her caregivers are or her home location details

- One caregiver emphasized the importance of caregivers waking up and preparing their children early so that they don't make their colleagues late. Parents should wake their children up by 6am and prepare for school because you find some children sleeping up to 8am and not interested in going to school. Parents should monitor such children so that they go to school by 7 as it's a parent's responsibility.
- One of the caregivers requested that this kind of teaching becomes a routine activity as they would like to be continuously educated on how to support their children since majority never went to school and don't know how to support their children. "Thank you [ORGANISATION] and call us again and again and we shall become better parents to our children".
- One caregiver encouraged others to fully participate in the homework support groups since being present with the children creates a sense of responsibility amongst the children and they would not if the caregiver supervises. He noted that this would also show the children that their parents want them to study hard and be important people in the community.
- One caregiver appreciated strategy 5 and noted that some children who are fast learners can be able to support low learners and improve learning outcomes. He also noted that it creates unity among children and parents as they continue to associate together and grow stronger bonds.
- One caregiver noted that when they bring their children to school, they can negotiate with the school in case they don't have money to pay for PTA and ask for payment plan.

Questions:

1. What will determine the extension of this program to the rest of the classes, this is really something that we would love to be rolled out to the whole school as teachers as it will really improve learning outcomes of the children in addition to caregiver engagement. This was answered by the RC
2. One caregiver wondered why majority of the programs that are always introduced in the school only target primary 3 class alone leaving out others. An example was given for [organization programme names]. The RC gave a satisfactory explanation of this
3. How will parents support the children with the homework support groups yet they themselves are uneducated and can't do much. Even at home caregivers don't have chalkboards for teaching these children, so how will this be done? This was ably answered by the RC.
4. One caregiver noted that some children steal other children's books, you find children who come home with more books or sets grabbed or stolen from their colleagues. This was responded too by the director of studies and P.3 B class teacher who were in attendance and noted that parents have the right to double check what their children bring home from school. Parents should also check the number of books that their children bring to school and take back home. In case a child brings home more than he took, it's the parent's responsibility to question the child where they got the extra materials and bring him/her to school for questioning so that the child doesn't grow the habit of theft.
5. What will be done for sick children who miss classes on some weekdays and have genuine medical reasons for missing class e.g., a medical report? This was ably responded too by the RC

Feedback on the Visuals

- One care giver noted that teachers be made to put on trousers not shorts.
- The children should also be in uniform
- The children (girls) should have short hair like the boys as they don't look like school children in the current pictures. "they should cut off the girls hair as we don't allow growing of hair in primary school".

Mobilization for the future strategies:

1. 1.On school companions: The care givers were encouraged to wake up their children early enough and prepare them so that they leave together as a group to avoid delaying others.
2. On recognition: A very clear explanation was given to the care givers on how this would be done and they were satisfied with the explanation.
3. Homework support groups: The children would be allocated according to where they come from basing on the information given by the care givers during enrolment and parents pledged their support.
4. The caregivers were informed of the next dialogue meeting which would be during the first month of 3rd term. Their children would inform them of the meeting and the venue would still remain the same.
5. Time management for next meeting was also emphasized

Challenges:

- Late coming of participants which delayed the start of the meeting.

This was however much better than the first meeting as the meeting started 30min past 2:00pm compared to the first meeting which started one and half hours later.

<u>Strategy 1 & 2: Meeting 2 - P3 stream A</u>

Activity: Meeting with Caregivers of P3A at [SCHOOL NAME]

Venue: [SCHOOL NAME]

Date: 8/ 10/ 2022

Total participants: 83

Teachers: 13

Introduction:

- The meeting was opened with a word of prayer led by a volunteer from the caregivers. An introduction was done for the research team and the teachers present. The languages used were Juba Arabic, Kakwa and Lugbara.
- Introductory remarks were given by the head teacher who welcomed the research team and caregivers to the meeting.
- More introductory remarks were given by the presenter highlighting why it was important to have the meeting with the caregivers at that time. She appreciated the caregivers for their big turn up and urged to keep up with the spirit.

The 6 strategies were explained to the caregivers to remind them again as:

1. Entry meetings with school and caregivers
2. Translation
3. Enrolment
4. Attendance monitoring
5. Homework support group
6. Accompaniment to school.

- All these were explained in detail to the caregivers including those that were already completed. They were reminded of what has so far been done, how it was done and what was yet to be done and how the caregivers were involved in the different strategies.
- After that brilliant explanation, the caregivers were asked to confirm whether those were the very strategies told to them from the beginning and they all confirmed that they were indeed the ones they had been informed about in the first entry meeting.
- The caregivers were explained on how the enrolment went on. Those who brought their children for the enrolment were appreciated for their efforts. Those who did not were further encouraged to enroll their children at the beginning of every school term as it is a requirement from ministry of education and the school.
- The caregivers were further explained too how attendance monitoring was being done. They were notified that attendance had been monitored for three successful weeks and the fourth week had been interrupted by independence celebrations and other school activities. This was clearly understood by the caregivers and teachers.
- Caregivers were explained too how the stars were awarded and they testified that their children had indeed informed them that they usually put stars against their names every Friday afternoon. They were told that a child who attends for all the 5 days gets 2 stars, the one who attends for 4 days gets 1 star and the one who attends from 3 down words, does not get any star. The star chart was shown to the parents and they were happy for that innovation.
- The criteria for awarding the children at the end of the 4th week were also explained to the caregivers. Saying those with 8 and 7 stars will be rewarded. Those with 6 stars and below, will not get any reward. The caregivers

who knew that their children were attending regularly beamed with prided, others were seen nodding their heads in appreciation for the program.

- Caregivers were then taken through the strategy of Homework support groups. They were informed on the rationale for forming these groups and their aim including the criteria for the formation of the groups (like basing on academic capability, location and distance form one homestead to the other).
- Those caregivers already participating in this strategy the exercise were appreciated for good time management, and organizing the place very well for the children to sit as that was noticed by the research officer while monitoring the activity. More were requested to volunteer and 25 caregivers volunteered to host the homework support groups.

REMARKS AND OBSERVATIONS

- Teachers remarked that the P3and AEP L1 learners are the first to arrive at school and their level of discipline has greatly improved. They said that they had noted that there are groups of some children who are competing to arrive first at school.
- One of the caregivers commended the program saying it has made his daughter to wake up early to come to school as compared to those days when she didn't have any interest in school and had almost dropped out. Thanks to the star charts the daughter has not missed school for a single day since the term began. Other caregivers noted the same for their children as they have seen an attitude change towards education in their children.
- The teachers noted that the learners love for education has improved. This is witnessed in some of the children's statements to their friends saying they want to be coming to school every day even if their parents say they should go to the garden, they will run to school because they want to get all the stars.
- The teachers noted that the attendance and level of concentration in class has tremendously improved since the children can now sit and wait for teachers in class.
- Teachers greatly commended the program as it has made most children to learnt the spellings of their names since they are the ones who physically attach the stars against their names.
- The teachers commended the program and requested that it be rolled out to the whole school so as to improve attendance and behavior of learners.
- Caregivers were also informed that the children who miss school due to sickness are noted as sick in the register rather than missed attendance. They were however informed that they have to come to school and inform the class teachers when their children miss school due to sickness.
- Teachers noted that the caregiver engagement has bridged the communication gap that existed between the caregivers, community and the school, they were very grateful for this program. Also there is mutual understanding between the caregivers and school thanks to the translation during meetings as both parties are able to communicate with each other.
- The Headteacher appreciated the caregivers for their huge turn up and asked them to continue with that spirit. She thanked [ORGANISATION] for bridging the gap that previously existed between the caregivers and the school and making communication easier. The school was able to utilize such meetings to pass on other communication to caregivers such the government program of Education Information management system (EMIS).
- Caregivers who had missed out on the enrolment week requested [ORGANISATION] and the school to allow them register their children and apologized for not taking the intervention serious as they didn't know that it would have such an impact on their children.
- A closing remark was given by the school management committee (SMC) chairperson who appreciated the parents for showing interest in their children learning and encouraged them to keep up the good spirit. He also appreciated them for keeping time for the meeting and turning up in huge numbers.
- The meeting was then closed with a word of prayer by one of the volunteer caregivers.

<u>Strategy 1 & 2: Meeting 2 - P3 stream B and AEP1</u>

Activity: Meeting with Caregivers of P3B and AEP Level 1

Venue: [SCHOOL NAME]

Date: 15/ 10/ 2022

Total participants: 84

Teachers: 19

Introduction:

- The meeting was opened with a word prayer from one volunteer caregiver. This was followed by introduction of the teachers present, translators and the research officer.
- The headteacher of the school was present and gave opening remarks including welcoming the caregivers for the meeting and thanked them for the big turn up and time management. She also urged the caregivers to feel free to ask questions whenever they have not understood so that everything can be made clear for them.
- The research officer welcomed every one present and thanked them for the huge turn up then went straight to explaining why the meeting was called (mobilize for the homework support groups and accompaniment).
- Some caregivers requested to explain the strategies on the visuals banner after the presentation which they gladly did. It was very amazing to hear caregivers explaining every picture step by step, how the activity was done and how the remaining strategies were to be done and how they were to be involved. That was so exciting to both the research officer and the teachers.
- Those caregivers who did not understand the explanation given by their colleagues on the burner explained too by the research officer and then informed that they missed the first meeting. They were cautioned to never miss school meetings for the sake of their children's learning and school growth.
- The session started with the chairperson PTA who appreciated the efforts of those who have wholeheartedly supported the program and urged them to continue for the betterment of the school and their children.
- In a special way, the research officer thanked the parents who brought their children for registration and urged them never to miss enrolling their children themselves at the start of each school term. Those who missed the enrolment were asked why they missed and encouraged never to miss enrolment as it's the first step of a child's learning and motivates the child to keep in school.
- A clear explanation was given on how the stars were awarded to the children and placed on the chart. That is 2 stars for someone who attends from Monday to Friday (capturing both morning and afternoon attendance) and one star for the one who attended 4 out of 5 days of the week. This explanation was given by the P.3 class teachers present.
- A thorough explanation was also given on how the children were awarded since that became a contentious issue as these caregivers arrived for the meeting. They were told that those with 8 and 7 stars were the ones who went home with a reward. Those who had 6 stars and below were not given anything because they did not attend regularly.
- Caregivers were reminded of the 2 strategies running how encouraged to participate in helping supervise the homework support groups and also prepare their children early morning for accompaniment. They promised to participate in the exercise and many more volunteered to support in the homework support groups.

REACTIONS

- Majority of the caregivers were very appreciative to [ORGANISATION] for the innovative interventions of encouraging the children to attend school regularly. They testified that the star chart exercise was able to make children come to school every day and timely.
- Caregivers also testified that these strategies have made the P3 and AEP level 1 learners to become friendly and united. They said the children are seen borrowing books among themselves from home to complete work that was not finished from school, something they had never seen before.
- Caregivers appreciated the homework support groups strategy and said it has helped to settle down children to open books over the weekend something that used not to happen in their village. They pledged to continue supporting the program.
- Teachers noted that this intervention is so wonderful because it has reduced the number of children who used to play outside during class hours. All P.3 and AEP level 1 children enter class and revise their books even when there's no teacher in class, something that the nearby classes of P.4 and P.5 are starting to copy.
- A caregiver asked to know whether those who did not receive up to 8 stars for the 4 weeks would be given another chance to attain the 8 stars. She was informed that the monitoring was a maximum of 4 weeks and would then be resumed for another 4 weeks. She was also informed to encourage her child not to miss attendance for the next round but also keep attending regularly even when there are no rewards.
- One caregiver complained that the daughter said they are 2 in the same stream with the same names and the stars are always given to the other girl leaving her out of the exercise. The class teacher replied her that the daughter had not been registered and the name was not on the star chart board. She said the stars are being put by the rightly owner of that name since she was the one who registered her and was brought for the registration by her mother whom she even knows. When this caregiver was asked as to when she registered the daughter, she said a week ago. Then she was told that the girls name was not in that list of the stars because she was registered late. She would be included in the next round. The research officer then advised the class teachers to request parents to add an initial for such names that are the same to avoid such complains and confusions.
- A caregiver requested to know how those who fall sick and have missed are treated in this regard of star putting. He was advised to inform the class teacher of that child so that that child is marked as sick but not absent. They were however cautioned not to use sickness to stop children from coming to school and instead take them to the garden.
- One caregiver regrated as to why she did not come to register her child to get the soap. She said she would have been relieved from buying soap for some time moreover soap is very expensive and UNHCR does not give them of late. She cautioned the rest of the caregivers who were like her to take their children's learning serious since they never know what ways they will be supported. She urged other caregivers to never miss registration as she was extremely regretting her inability to participate in the exercise and said that this meeting had changed her attitude towards her involvement in the learning of her child.
- A caregiver requested to know what can be done to a child who does not want to move in a group with others for the accompaniment to school. He was explained the importance of children coming to school together and urged to use those benefits to convenience such a child.
- A parent requested to know whether the caregivers supervising the groups formed for homework will also be rewarded. Majority of the caregivers laughed at her question. The School Management Committee chairman who was in attendance requested to answer this question and expressed his disappointment saying there would be no pay for that. He wondered why caregivers were asking for rewards for everything as if the children are for [ORGANISATION]. "These children are yours, and you should take the first initiative to educate them before looking for rewards". He thanked those who have also volunteered to support the home work groups and encouraged all caregivers in with children in other classes to do the same even when the pilot with P.3 and AEP level 1 is complete.
- He also warned caregivers who come for meetings and go back home to mobilize others to de-campaign the school saying they will be delt with accordingly.

- The meeting was closed with a word of prayer by one volunteer caregiver after closing remarks by the headteacher.

<u>**Strategy 3: Enrolment week**</u>

Report on the Enrolment of P.3 and AEP Learners at [SCHOOL NAME]

- The exercise went well for the first day.
- The learners first arrived at school without their caregivers and were sent back home to come with them for enrolment. The turn up was surprisingly high which showed that the caregivers had understood the reason they were invited to school.
- There was no learning as the major activity that took place was registration/enrolment and general school cleaning by both the learners and teachers.
- The head teacher and teachers noted that the first week of the term is always the enrolment week and there's any learning that takes place during that week as majority of the learners don't even turn up for lessons.
- The exercise started well with a big turn up of caregivers and the learners for registration. We had a warm reception for the activity from the school. Four out of six teachers who were trained for the exercise participated in the registration on the first day.
- The total number of children registered on Monday 5th September 2022, was 81.
- The total number of children registered on Tuesday 6th September 2022, was 41.
- The total number of children registered on Wednesday 7th September 2022 was 12.
- One tablet was left at school for the teachers to register the caregivers and children that continued to come for enrolment throughout the week

Observations

- Some caregivers were still coming alone leaving their children behind. They were advised to go back and return with their children. Only caregivers who came with their children were enrolled.
- Equally some children came without their caregivers giving reasons such as caregivers being in the gardens, they were advised to notify them and come for enrolment the next day or in the afternoon.
- Some caregivers were also sending their colleagues to register their children on their behalf. They were advised that only a caregiver of a child has the right to register them and not their neighbors or friends.
- Some of the caregivers who are not primary contacts on the attestation cards wanted details of the primary contact captured instead of themselves as the ones that have brought the children for enrolment. They were explained too that the caregiver being captured in this exercise is the one present that has escorted the child for enrolment and then any other caregiver in the household.
- The school usually conducts registration for payment of PTA fees at the beginning of the term. The school hadn't prepared for this when the enrolment began and the parents requested that these two activities be organized together so that the parent doesn't have to come to school multiple times. The head teacher was able to organize for this to happen at the same time as the enrolment.
- Some of the children were registered in different settlements e.g. [settlement name] but study from [different settlement name]. Their details (individual numbers) were shared by their relatives where they registered from and the caregiver came with this information written on paper though they didn't have the original attestation cards for verification.
- The caregivers were really positive with the exercise even when they were asked to go home at lunch break and return in the afternoon, they insisted in staying with their children and getting the exercise concluded. There resilience was humbling to the research team and the teachers.

- An extra ordinary case was noted where one caregiver who was sick came to enroll her child. She had a canula and the other caregivers noted that she had recently had surgery and they unanimously agreed to let her skip the queue, be attended too first and go back home. This kind gesture by the rest of the caregivers was equally humbling.
- Some caregivers noted that some details captured on the attestation cards were wrong such as dates of birth and insisted on giving the correct dates as they're the biological parents, instead of capturing the wrong information on the attestation cards.
- It was noted that all children didn't know their dates of birth even when they were written on the attestation cards. There were sensitized by the teachers that they should know their dates of birth as its very important for their own good.
- All children were very confident of the streams they belong too which was really great to note.
- The teachers needed continuous guidance on the enrolment not to give in to the caregivers demands but rather stick to the agreed procedure e.g., capturing the details of the caregiver that has come to enroll the child and probe carefully on the health conditions of the child if any.
- The case of [child name 1 and 2] was also verified. The Research Officer discovered that the name [child name 1] was what the learner was using previously, it's the name she likes because it was given to her by her father and was the name known at school by everyone. During registration, the mother came and registered her with the name on the birth certificate [child name 2] which she does not want and wanted it changed back to [child name 1]. The Research Officer explained to her the importance of using the name on her birth certificate for registration and she finally was okay with that.

Challenges

- Some of the teachers capturing the enrolment data were slow which frustrated some caregivers and they lamented on spending longer time with them compared to others.
- Rains disrupted the process especially in the afternoon hours and this made parent to come only in the majorly in the mornings. Turnup was really low in the afternoons due to heavy down pour
- Some caregivers who didn't have children in P.3 were not happy that their children were not captured on the tablets even when they were explained too the purpose of the activity. Their information was captured in the note books as usual.
- One of the children has not been registered by OPM and so didn't have an individual number. Therefore, she was not able to be enrolled via Kobo but in a notebook which she wasn't happy about.

Strategy 4: Attendance monitoring (week 1)

Observation from week one of attendance monitoring at [SCHOOL NAME] (P.3 mainstream and AEP Level one)

- The first week of active learning and marking of the attendance register started on 12th to 16th September 2022.
- Upon consistence attendance monitoring for a week, on the Friday 16th in the afternoon, the P.3 class teachers under guidance of the research officer put up the star charts in the class room which bore only the children's names that were obtained from the enrolment register. This excited the learners when they were explained too the rules of the attendance monitoring (2 stars for 5 days full attendance and 1 star for 4 days attendance, no star for 3 days and below attendance.
- The teacher would read each child's name in the register with followed by the number of days attended and the child would be given a star to put besides their name.
- This was really exciting for the learners as each wanted a star against their name and those who didn't get stars due to their irregular attendance were sad. It was during that moment that the teacher would explain to the child why they did not get the star and encourage them to fully attend nest week if they were to get any starts.
- The learners were very excited to see their names on the star board and were extremely excited to touch and put the stars against their names. One of the learners after putting the stars against her name shouted out in excitement that "I do not want to miss coming to school and I want to be the best attender from our class." It was really an exciting exercise for the learners to participate and to see everything for the first time. One child from primary five who was watching what was taking place from the window ran to the research officer after the exercise and asked when theirs would begin. He was informed by the P.3 class teacher that this was a pilot exercise in P.3 which would be rolled out once there were results from this pilot.
- Some of the learners who were not registered by their caregivers were really sad when their names were not on the noticeboards. They were informed to tell their caregivers to come alongside them for enrolment so that their proper details would be captured. Then their names would be added on the notice boards and their attendance reflected as the rest of the learners.
- One learner from P3 A who had not been enrolled by the caregiver pretended to be the friend whose name was called and he hurried very fast to pick the stars from the teacher to put them on the chart, but the teacher was quick enough to recognize that he was not the true owner of the name. When the teacher asked the children in the class whether that name belonged to this imposter, the class shouted no and the boy was warned to desist from such behavior. The whole class laughed and other children told the teacher that there were many who were planning to do the same. The teacher had to warn them.
- Some learners who were registered late but came to school for the whole week or missed only once and their names were not (printed during the star activity) were explained too, that they would put their stars when the names are brought to school the following week. This was meant not to disappoint them and to keep their spirit up for the exercise.
- After the exercise, the teachers shared their experiences and commended the whole intervention saying it will improve on the attendance of the learners greatly and they pledged to do the work whole heartedly. The teachers noted that the exercise took a longer time (25-30 minutes) because it was the first time and the children had not marked where their names were on the star chart and notice board.
- There were many children in the three streams who were not registered but were attending classes. The teachers mentioned that most caregivers come to register/enroll their children when they see something good at hand. They even mentioned that we should not get surprised to see many come to register even from other schools after giving rewards to the regular attenders.
- Some of the children were still confused of their names since they were not used to the ones on the Attestation cards that were used during enrolment. This was handled by the class teacher who knew exactly who these children were and who their caregivers were. They were advised to know their names by heart;

as those being called which are similar to those on the attestation cards as their official names. They were also encouraged to tell their caregivers and friends to call them by their official names compared to the nicknames.

- Some children who were enrolled at the beginning of the term have never stepped in the classroom for lessons from the beginning of the week. The teachers noted that these could some who register in multiple schools, so came to also register in [SCHOOL NAME] thinking there was going to be an immediate benefit from the school.
- Some of the learners who were supposed to be in steam C as they moved from A and B because of space shortage in the two streams mentioned their former streams (A and B) during registration instead of C and this made their names to feature in those other streams instead of C. These names were taken to stream C where they are studying from for proper monitoring and this was agreed upon by the teachers.
- Most of the learners could not read out and locate their names from the star charts and this caused the exercise to take longer than anticipated. The teachers agreed that they will work together with some of the best performing children in the class to help them read out and locate the names from the star chart so that the exercise does not consume a lot of time next time. These children will stand near the noticeboard and help some identify their names on the star chats.
- There were some spelling errors noted from the names entered by some teachers. The teachers agreed that they should come up with the whole list of those names with errors then present them for an action based on the advice from the coordinator. However, they were able to tell what those names were and how they should have been written.
- One learner had no name but only a number that is [registration number]. This made it very difficult to identify who the child is.
- There were variations in some names, for example, there is a name known by teachers as [child name 1], but it came out to be [child name 2]. The teacher took it to be [child name 1] saying there is no body called [child name 2] in P3. This could be either an error that was made by the teacher during enrolment or the correct name of the child on the attestation card is [child name 2] instead of [child name 1] as commonly known. This particular case needs to be verified.

<u>Strategy 4: Rewards celebration</u>

REPORT ON THE AWARD SSESSION FOR P3 CLASS ATTENDANCE ON 14TH SEPTEMBER 2022 AT [SCHOOL NAME].

The 14th September 2022 was a beautiful day at [SCHOOL NAME] for both the P3 learners and the entire school. As I reached the school in the morning and started offloading the boxes of soap to the Headteachers office, the learners who were sure of getting the reward started jubilating to receive their rewards and yet the session was meant to be in the afternoon.

The morning hours became too long for these learners to wait that some of them came and tipped the research officer to begin the program at lunch time since they were not hungry but rather ready to receive their gifts. They were then told to hurry for food home and come back early for that program.

When time came for us to be in class for the session, those who were to receive could not hold back their joy. They started clapping for the team that carried the soap plus the teachers to receive them into the classroom shouting we are happy! we are happy! we are happy! Till when we entered the classroom.

The research officer reminded them of the criteria of giving out the rewards as those with 8 and 7 stars would be the ones to receive a bar of soap each because they were the ones who came to school regularly. Those with 6 stars and below were not getting because they were not coming to school every day and were encouraged not to miss in the coming month both in the morning and afternoon so that they can also receive the gifts.

Those who were rewarded promised not to miss in the next program so that they can get the reward again.

The class teachers were also very happy and joined those rewarded in the jubilations and encouraged them to continue coming regularly to school not only for the gifts but so that they can improve on their performance.

The session went on very well in all the three streams of A, B and AEP L1. Those who were rewarded were very excited throughout the session and those who did not receive the gifts cried. Out of the 156 learners registered in the first week, 116 got the reward.

The research officer appreciated all those who got rewards and encouraged them to also participate fully in the next session. She thanked the class teachers for their support in the entire program and encouraged them to continue supporting programs that come to school like that.

The Headteacher closed the session by thanking [ORGANISATION] for keeping their promise saying there are many organizations that make promised and do not fulfil them. She encouraging the children not to be looking at rewards for coming to school. She encouraged the learners to be committed in their learning even without these rewards. She appreciated those who came to school without missing and encouraged them to keep up with that spirit even when there are no reward. And encouraged those who did not get to work hard so that they can get next time.

Strategy 5: Homework clubs

Plan for Homework support groups

Upon discussing this strategy with the teachers, below were their suggestions and concerns.

- Teachers suggested and agreed to give an individual homework on Friday 23rd to prepare the learners for the following week.
- The teachers noted with concern that when grouping the learners for the homework support group, all the children in the three streams i.e. A, B, C be put together and then grouped accordingly. They noted that the grouping should not be done based on streams because some of the children come from the same area but are in different streams and this will make them get scattered and far apart from each other when working from home.
- The teachers agreed to find time during the evenings and sit together do the grouping.

HOMEWORK SUPPORT GROUP

The caregivers are impressing the homework support group exercise. There are many who have willing volunteered to support program. They are ready to prepare places for the learners to settle and do their work and to also give the children the necessary support needed to do the work.

The learners are also following up the program very well and do not want to miss the exercise. This was confirmed to the research officer on Saturday by one of the caregivers who testified that the children are serious with the work given to them and they do not want to miss. She said that she had told her daughters to go to the well to wash their clothes but these girls said they were first doing their homework before going to the well since they were told to do that in the morning.

Indeed these children mobilized themselves and the caregiver settled them down to do the work. When the research officer was going round homes to monitor the activity, she confirmed that the children were serious with the homework and being supervised by their caregivers.

OBSERVATIONS.

Some caregivers complained of the time for doing the homework saying it should be in the afternoon hours since the morning hours are for their garden work. They were told that the homework is meant to be done in the afternoon hours but since there was a meeting that afternoon, the exercise was shifted to the morning hours.

Some homes do not have what to sit these children on during the exercise, but the caregivers said they are willing and ready to support the children even if it is to make them sit on small sacks, they will do that because they want to support the program.

Caregivers requested for the number of questions given for the learners for homework to be increased from 7 to 10 so that these learners can sit for a longer time. This concern came up as a result of some caregivers who complained that the children in the village are spending most of their time watching films instead of reading books.

When the teachers were conducted on that, they were willing to increase the number of the questions to 10 as suggested by the caregivers.

Generally, the exercise is being given the attention it deserves by both the parents and the learners.

<u>Strategies 5 & 6: Homework clubs and Safety to School</u>

Progress Report on SchoolLinks protoyping Pilot (Homework Support Groups and Accompaniment).

- The learners of P.3, AEP Level 1 and P7 are the first to reach school with the last person arriving at 7:40am. This has been witnessed by the RO several times
- There is a boy on the wheel chair who has tremendously ensured that he reaches school early with the support of the friends in his homework support group. This was been praised by the teachers as they have observed children supporting each other in all aspects.
- Teachers have commended the P3 and AEP Level 1 children for always coming early to school, and ensuring the cleanliness of the school for the teachers on duty.
- The P.3 and AEP Level 1 learners have also been seen seated in class without playing waiting for teachers after finishing with cleanliness.
- The have also been observed to be clean in the school since they always want to look smart in the photos taken at any chance they are met.
- The P.3 and AEP Level 1 learners have become rare on the playground during class hours especially in the morning hours. Something which has pleased the school management.
- The homework support groups have received tremendous support from both the children and the caregivers. The children now demand for homework from the teachers every Friday so that they participate on weekends.
- The caregivers have noted that homework support groups have helped to settle down children over the weekend and they have pledged to continue supporting the program. They said that some groups even sit and do more than two subjects on that Saturday. Meaning, they are getting to learn the importance of reading from home over the weekend. They caregivers also request that the children be given a sizable amount of homework for the children rather than the few numbers they're given.
- Teachers also noted that much as marking the homework has increased their work load, still it is very good because they now don't see P.3 and AEP Level 1 children loitering a lot in the afternoon in community like they used to.
- On the strategy of accompaniment, the children reach near school early and wait for their friends to then come in a group. The children say their parents wake up early for the garden (6:00am) and they don't prepare them for school. this makes them wake up late and rush through everything to get to school early. They say their parents have not been involved in this strategy like the homework support groups.
- The lead teacher has commended the strategies and said he is going to advice [different organization] to copy some of these strategies especially the one for attendance monitoring to retain AEP and other out of school children in school.
- The number of caregivers reaching to school to report about the learners missing school because of sickness has also improved. Previously caregivers were not involved in coming to notify teachers when children are sick but they have greatly taken up the advice given during the meetings with them and improved on this.
