# Supplementary material for: Increasing school attendance and retention in refugee settings: Participatory development of the SchoolLinks intervention
Source: PLoS One. 2026 Aug 31;21(8):e0357215. doi: 10.1371/journal.pone.0357215 (PMC13528952; doi:10.1371/journal.pone.0357215)
Supplement: Supplementary file 7 [file pone.0357215.s007.pdf]

Inclusivity in global research

PLOS' policy on inclusivity in global research aims to improve transparency in the reporting of research performed outside of researchers' own country or community and ensures that PLOS publications reporting global research adhere to high standards for research ethics and authorship. Authors of relevant research articles may be asked to complete the questionnaire below, which outlines ethical, cultural, and scientific considerations specific to inclusivity in global research. This questionnaire may be requested when researchers have travelled to a different country to conduct research, if research uses samples collected in another country, research with Indigenous populations or their lands, or if research is on cultural artefacts. Researchers travelling to another country solely to use laboratory equipment will not normally be required to complete the questionnaire. However, the questionnaire can be requested at the journal's discretion for any submission – if you have been requested to complete this questionnaire by the PLOS journal you submitted to, please do so.

Please complete the questionnaire below and include this as a Supporting Information file with your manuscript. Note that if your paper is accepted for publication, this checklist will be published with your article in the supporting information files. Please ensure that you reference the checklist in the main body of your manuscript. We suggest adding a subsection 'Inclusivity in global research' to your Methods section and adding the following sentence: "Additional information regarding the ethical, cultural, and scientific considerations specific to inclusivity in global research is included in the Supporting Information (SX Checklist)"

The questions have been designed to be applicable to a wide range of study types, and there are subsections for both human subjects research and non-human subjects research. If any of the questions are not relevant to your research please mark them as "N/A" as appropriate.

Ethical considerations, permits and authorship

This section is applicable to all research types.

Provide details as to who granted permissions and/or consent for the study to take place in the Methods section of your manuscript. This should include the names of **all** ethics boards, governmental organizations, community leaders or other bodies that provided approval for the study. If individuals provided approval refer to these people by their role or title but do not list their name(s).

Reported on page number: 16<br>Permissions were granted by Makerere University's School of Social Sciences' Research Ethics Committee, The AIDS Support Organisation Research Ethics Committee, and Uganda's National Council of Science and Technology. Additional permissions were granted by the Office of the Prime Minister (which coordinates the management of the refugee settlements), community leaders in each of the four settlements, the School Management Committees of each school (which include the school principal), and the Parent-Teacher Associations for each school.

If there were any deviations from the study protocol after approval was obtained please provide details of these changes in the Methods section of your manuscript.

N/A

Did this study involve local collaborators that are residents of the country where the research was conducted or members of the community studied? If you do not have any authors from said communities, please provide an explanation for this below.
Everyone listed as an author should meet PLOS' criteria for authorship and all individuals who meet these criteria should be included in the author byline, rather than the acknowledgements. For further information please see the journal's Authorship Policy.

Yes, Victo Kyobutungi and Hellen Ayozu are residents of Uganda who collaborated on the research, and both are named as co-authors.

Human subjects research (e.g. health research, medical research, cross-cultural psychology)

Did you obtain written informed consent from a representative of the local community or region before the research took place? How did you establish who speaks for the community? Details of written informed consent obtained from study participants should be reported separately in the Methods section of your manuscript.

We obtained written or witnessed informed consent from participants (see p. 11 & 14), but not from community representatives. Instead, Victo Kyobutungi held inception meetings with community leaders and representatives of local government (including Office of the Prime Minister, responsible for the refugee settlement management) to explain the purpose and methods of the research and respond to questions and concerns. Victo was supported in these meetings by research assistants who were recruited from both the refugee settlements and the host communities and provided translation into multiple languages. Community representatives agreed to and supported the research, and formal authorisations were given by the Office of the Prime Minister. Victo was identified as the most appropriate member of the team to facilitate these meetings in her role as research coordinator and, with the support of the research assistants, was familiar with the appropriate customs and languages.

How did members of the local community provide input on the aims of the research investigation, its methodology, and its anticipated outcome(s)?

In addition to the inception meetings described above, information and consent sessions were held by Victo and the research assistants with participants and members of the school communities (including caregivers, teachers, and school leadership). In each phase of the research, participants' input and feedback on the intervention was actively sought; this was a cornerstone of the intervention development process.

When engaging with the local community, how did you ensure that the informed consent documents and other materials could be understood by local stakeholders?

The informed consent documents were translated into the most prominent languages used by participants in different locations (including Runyankole, Swahili, Bari and Juba Arabic). To account for additional language preferences and low levels of literacy, research assistants went through the information and consent forms verbally with groups (formed according to language). To introduce the intervention, we used a visual poster which did not contain text to avoid exclusion based on language or literacy.

Will the findings of the research be made available in an understandable format to stakeholders in the community where the study was conducted (e.g. via a presentation, summary report, copies of publications, etc.)? Please provide details of how this will be achieved.

Yes, we hold gatherings similar to the inception meetings to disseminate the research findings. These follow local customs, such as being opened and closed with prayer and including speeches from government and community representatives. To be inclusive of multi-lingual and low-literate communities, we try to avoid written communications and instead use infographics, pictures and story-telling to maintain inclusivity during dissemination.

Non-human subjects research using specimens/ animals collected as part of the study, or those housed in archival collections. Examples include archaeology, paleontology, botany and zoology.

Did the permission you obtained from a local authority to perform the study include an agreement on access to outputs and benefit sharing? This may include procedures to enable fair distribution of the benefits and resources arising from the research performed. Please include any details of Prior Informed Consent and Benefit Sharing Agreements obtained. These may be required by field-specific regulations, for example the Convention on Biological Diversity (CBD) and the associated Nagoya Protocol.

N/A

If the material used in your study was imported, please A) provide the year it was imported and B) indicate whether permits were obtained to import/export the materials used, C) provide details of any permits obtained. If this information is not available, please indicate this.

N/A

If you used archival specimens, please state how the material used in your study was acquired by the institute it is held in and provide details of any permits obtained for the original excavations/ sample collection. If this information is not available, please indicate this.

N/A

How was the potential cultural significance of the materials collected in your study to local communities considered in your research design? Were Indigenous peoples and/or local researchers and institutions involved with archaeological excavations / collection of specimens? If so, please provide a description of their involvement.

N/A

If your manuscript includes photographs of human remains please indicate whether authors obtained permission from descendants or affiliated cultural communities to do so.

N/A
